# Genomic characterization of avian metapneumovirus subtypes A and B in United States poultry by targeted amplicon sequencing

**DOI:** 10.3389/fcimb.2026.1882507

**Published:** 2026-08-21

**Authors:** Naveen Duhan, Mohamed H. Selim, Parfait Kada, Mohammed Luqman, Gun Temeeyasen, Jagathiswaran Radhakrishnan, Tamer A. Sharafeldin, Sunil Mor

**Affiliations:** 1 Animal Disease Research and Diagnostic Laboratory, Department of Veterinary and Biomedical Sciences, South Dakota State University, Brookings, SD, United States; 2 Department of Veterinary Pathology, Faculty of Veterinary Medicine, Zagazig University, Zagazig, Sharkia, Egypt

**Keywords:** avian metapneumovirus, epidemiology, G protein, phylogenetic analysis, poultry, targeted amplicon sequencing, vaccine-derived strains, whole-genome sequencing

## Abstract

Avian metapneumovirus (aMPV) subtypes A and B emerged in United States poultry in late 2023 and early 2024, prompting genome-scale surveillance from clinical samples. Here, we developed and optimized targeted amplicon sequencing (TAS) assays for both subtypes and applied them to 104 subtype-positive clinical samples collected from chicken and turkey farms across nine US states between early 2024 and early 2026. TAS recovered 91 genomes suitable for comparative analysis, including 44 aMPV-A and 47 aMPV-B sequences, with successful recovery extending to Ct values of 34.6 for aMPV-A and 31.2 for aMPV-B. Recovered genomes showed near-complete breadth and high mapping efficiency. Phylogenetic analyses of both G-gene and whole-genome datasets showed that US aMPV-A field strains formed a distinct monophyletic lineage within group IV and resolved into three closely related clusters. Cluster 2, first recognized in North Carolina and later detected in Ohio, spread across 30 turkey and chicken farms. Cluster 2 genomes were defined by a concentrated G-protein hotspot within residues 209–275, and most North Carolina Cluster 2 genomes carried 10–11 nonsynonymous substitutions in this region, including multiple proline substitutions suggestive of local structural change. Missouri Cluster 3 remained cohesive but distinct from both Cluster 1 and Cluster 2 in the G-gene and whole-genome trees. In contrast, US aMPV-B field strains remained highly homogeneous across hosts and states, with more than 99% nucleotide identity by both G-gene and WGS analyses. We also identified 12 vaccine-derived genomes on both vaccinated and nonvaccinated farms. These included six genomes related to aMPV-A vaccine and six related to aMPV-B vaccines (VCO3/50 and 1062), all of which retained vaccine-defining markers together with additional substitutions consistent with continued circulation after vaccine use in the field. Selection analyses showed that the G gene had the highest gene-wise dN/dS ratio in both subtypes. Additional elevated signal was observed in SH and M2, and candidate positively or episodically selected codons were concentrated in the subtype A Cluster 2 G-gene hotspot. These findings show that TAS supports direct-from-sample aMPV genomic surveillance and provides genomic context for field clusters, vaccine-derived lineages, and continued adaptive change in aMPV in US poultry.

## 1 Introduction

Avian metapneumovirus (aMPV) is a highly contagious respiratory pathogen that causes significant disease in avian species, particularly turkeys, chickens, and ducks (Cook, 2000; Hartmann et al., 2015). Turkeys are generally considered the most susceptible host species, but infection in chickens is also well recognized and can contribute to substantial production losses (Cook, 2000; Hartmann et al., 2015). Clinical signs are predominantly localized to the upper respiratory tract and include infraorbital swelling,sneezing, and swollen head syndrome. While morbidity is characteristically high, mortality remains relatively low unless complicated by secondary respiratory pathogens, which exacerbate clinical severity and elevate mortality rates (Jones, 1996; Cook, 2000; Hartmann et al., 2015). In laying hens and turkey breeders, although respiratory disease is generally less severe, infection can extend to the reproductive tract, resulting in reduced egg production and compromised egg quality (Jones, 1996; Cook et al., 2000).

aMPV is a nonsegmented, enveloped, single-stranded, negative-sense RNA virus belonging to the family *Pneumoviridae*, genus *Metapneumovirus* (Rima et al., 2017). The aMPV genome comprises approximately 13 kilobases of nucleotides organized into eight genes arranged in the order 3^′^-leader-N-P-M-F-M2-SH-G-L-trailer-5^′^, encoding nine proteins, as the M2 gene contains two overlapping open reading frames producing the M2–1 and M2–2 transcripts (Easton et al., 2004). Three of the nine viral proteins, the glycoprotein (G), fusion protein (F), and small hydrophobic protein (SH), are surface-exposed glycoproteins located on the viral envelope and are directly accessible to host immunity. The G protein mediates cell attachment and is the most variable among viral proteins, serving as the primary target for genetic characterization and subtyping (Juhasz and Easton, 1994; Catelli et al., 2010). The F protein facilitates cell fusion and viral entry into host cells (Easton et al., 2004). Although the precise function of the SH protein remains unclear, evidence of immune-associated diversification suggests it also contributes to aMPV evolution (de Graaf et al., 2008).

Based on genetic analysis of the G gene, aMPVs are classified into four main subtypes: A, B, C, and D (Juhasz and Easton, 1994; Bäyon-Auboyer et al., 2000). Subtypes A and B are the predominant poultry-associated subtypes reported worldwide and affect both chickens and turkeys (Cook, 2000; Cecchinato et al., 2016). Subtype C was first identified on turkey farms in the United States and exhibits greater variability in the length and nucleotide sequence of the G gene compared with subtypes A and B (Seal, 1998). Subtype C has also been detected in wild birds (Shin et al., 2002; Jardine et al., 2018; Canuti et al., 2019), and a distinct Eurasian subtype C lineage has been identified in ducks in France (Toquin et al., 2006), Italy, and China (Sun et al., 2014), as well as on chicken farms in China (Wei et al., 2013). Subtype D was initially detected on a turkey farm in France in 1985 and has not been reported since (Bäyon-Auboyer et al., 2000). In the United States, unlike the global epidemiological pattern, historical reports were dominated by subtype C (Seal, 1998). However, in late 2023 and early 2024, both subtypes A and B were confirmed in US poultry, causing major outbreaks and substantial economic losses (Goraichuk et al., 2024; Luqman et al., 2024; Zhang et al., 2025). In response to these losses, the USDA Center for Veterinary Biologics (CVB) granted conditional approval for imported commercial aMPV modified live vaccines (MLVs) based on European strains (USDA-APHIS, 2024). Five MLVs were approved, including one subtype A vaccine, Poulvac TRT (aMPV-A, K strain of turkey origin; Zoetis, Spain). Four subtype B vaccines: Vaxxon SHS (aMPV-B; Vaxxinova, Italy); NEMOVAC (PL21 strain, aMPV-B; Boehringer Ingelheim, France) and RESPIVAC aMPV (1062 strain, aMPV-B; HIPRA, Spain) for use in chickens; and AVIFFA RTI (VCO3/50 strain, aMPV-B; Boehringer Ingelheim, France) for use in turkeys.

The emergence of aMPV subtypes A and B in US poultry has created a need for detailed molecular characterization of circulating strains and close monitoring of their ongoing evolution. Unlike traditional Sanger sequencing (Sanger et al., 1977), next-generation sequencing (NGS) enables rapid, precise, and high-throughput whole-genome analysis that can identify point mutations, insertions, and deletions with high resolution (Dimitrov et al., 2017). Sanger sequencing remains useful for rapid confirmation of short loci and low-throughput follow-up because it is simple to implement and provides high per-base accuracy for limited amplicons. However, when multiple loci or larger sample sets must be compared, targeted amplicon sequencing (TAS) and related NGS approaches provide higher throughput, broader mutation detection, and genome-scale context through multiplexing. In this setting, Sanger is well suited to a small number of targeted confirmations, whereas TAS is more practical for larger sample sets and genome-wide comparisons. Recent reviews of avian diagnostic sequencing have highlighted the growing utility of both targeted and non-targeted NGS workflows for pathogen discovery, genomic surveillance, and outbreak investigation in poultry (Afonso and Afonso, 2023). Recent U.S. outbreak investigations and retrospective subtype B genomic analyses have also documented the rapid spread of both subtypes and the recovery of representative subtype A and subtype B viruses for further characterization (Zhang et al., 2025; Selim et al., 2026a; Alvarez-Narvaez et al., 2025). However, direct sequencing of viral genomes from clinical samples without prior isolation or culture remains technically challenging, as metagenomic approaches often yield insufficient genome coverage and too few viral reads, particularly when host and non-target microbial nucleic acids dominate clinical respiratory samples (Afonso and Afonso, 2023; Cho et al., 2023).

The limited shedding window reduces the opportunity to collect clinical samples with high aMPV viral loads, thereby lowering the likelihood of successfully assembling whole genomes from metagenomic sequencing. TAS method can address this limitation by enabling direct whole-genome sequencing from clinical samples, including those with lower viral loads. This approach has been successfully validated for several important pathogens, including SARS-CoV-2 (Tyson et al., 2020), Zika virus (Quick et al., 2017), and rabies lyssavirus (Gigante et al., 2020). TAS has also been applied to recover aMPV-B coding-complete or near-complete genomes directly from clinical samples, including three subtype B genomes from Korean live-bird-market submissions with complete CDS recovery in samples with Ct values of 25.77 and 28.67 and 98.2% CDS coverage at Ct 30.6 (Cho et al., 2023). More recently, an amplicon-based tiled PCR scheme was optimized for the emerging aMPV subtype B in US poultry farms, enabling sequencing of complete or nearly complete genomes from samples with Ct values below 22 (Alvarez-Narvaez et al., 2026).

In this study, we used aMPV-A- and aMPV-B-positive clinical samples from the Animal Disease Research and Diagnostic Laboratory at South Dakota State University (SDSU-ADRDL) to develop and optimize TAS methods for direct whole-genome sequencing from clinical samples. We then applied this workflow to poultry submissions from multiple US states to define its recovery limits, describe the genomic epidemiology of circulating subtype A and subtype B viruses, characterize field and vaccine-derived lineages, and evaluate gene-wise and site-wise signatures of selection across the assembled genomes.

## 2 Materials and methods

### 2.1 Sample collection

**Figure 1:**
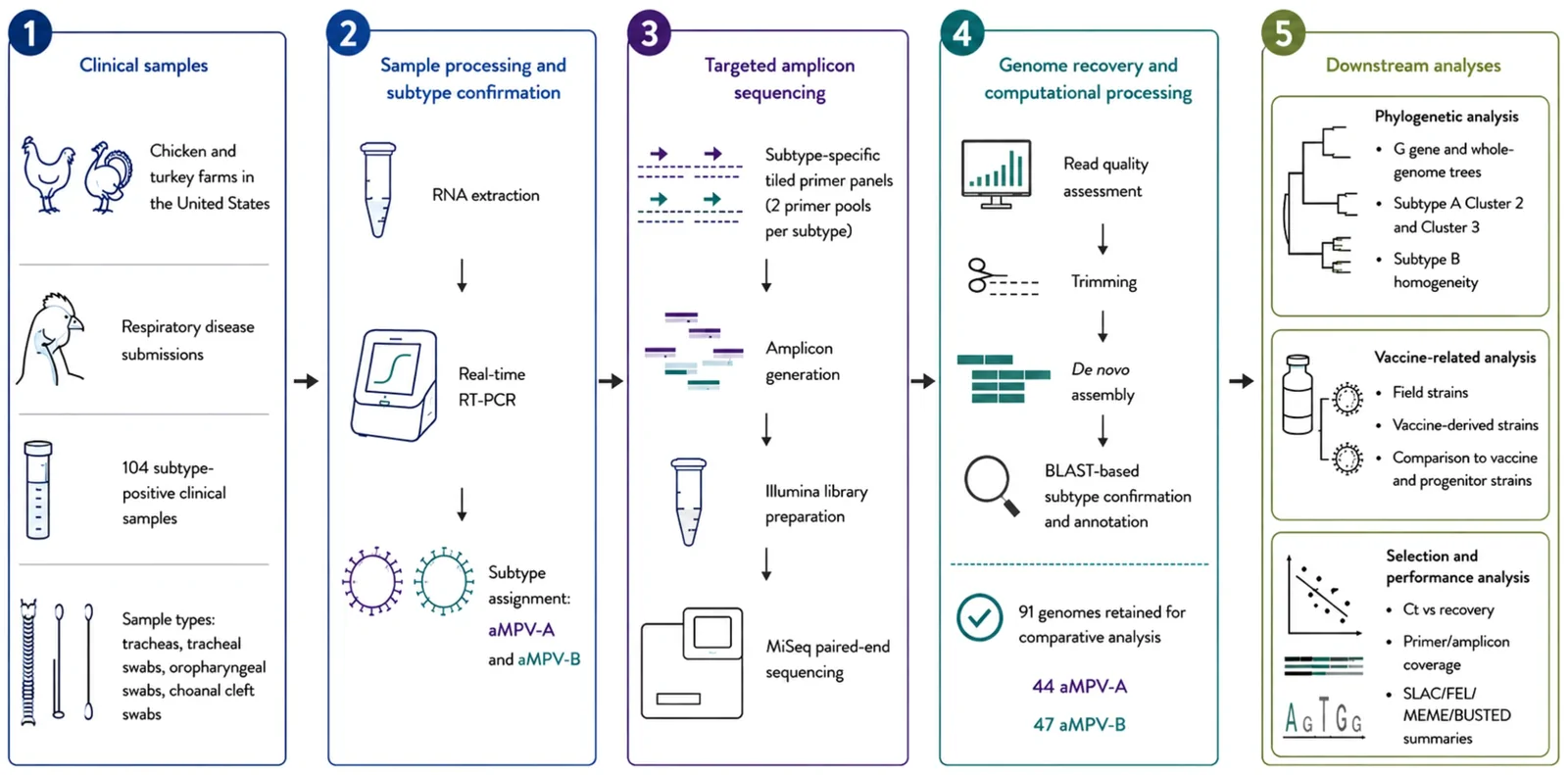
Study design and analytical workflow for direct-from-sample genomic surveillance of aMPV subtypes A and B in US poultry. The workflow begins with pooled clinical respiratory submissions, followed by sample processing, RNA extraction, subtype-specific real-time RT-PCR, tiled amplicon amplification, Illumina sequencing, and downstream comparative analyses including sequencing-performance assessment, phylogenetic reconstruction, and selection analysis.

A total of 104 subtype-positive clinical samples, including tracheas, tracheal swabs, oropharyngeal swabs, and choanal cleft swabs, were analyzed from chicken and turkey farms experiencing respiratory disease and variable mortality rates. The cohort comprised 91 turkey and 13 chicken samples originating from nine US states: North Carolina (n = 81), Missouri (n = 6), Minnesota (n = 4), South Dakota (n = 3), Virginia (n = 3), South Carolina (n = 3), Pennsylvania (n = 2), Ohio (n = 1), and North Dakota (n = 1). Samples were collected over a two-year period from early 2024 to early 2026. All samples tested positive for aMPV-A and/or aMPV-B by real-time RT-PCR, with Ct values ranging from 14.6 to 36.0, and all were carried forward for sequencing and downstream genomic analysis. For recovery-rate summaries, the unit of analysis was the subtype-positive clinical sample; phylogenetic and selection analyses were then performed on the final set of assembled genomes. A schematic overview of the laboratory and analytical workflow used in this study is shown in **Figure 1**.

### 2.2 Sample processing

Two sample-processing methods were employed depending on the sample type. For swabs, the pool of five swabs in virus transport media (VTM) was carefully homogenized, and 1 mL was aspirated and centrifuged at 2,000 × *g* for 10 minutes at 4°C. The clear supernatant was collected and stored at −80°C until further processing. For tissue samples (tracheas), five tracheas were placed in a Whirl-Pak bag containing 5 mL of VTM and homogenized in a stomacher for 1 minute. After homogenization, 1 mL of supernatant was centrifuged at 2,000 × *g* for 10 minutes at 4°C, and the clear supernatant was aspirated and stored at −80°C until further examination.

### 2.3 RNA extraction

Viral RNA was extracted using the EZ1&2 Virus Mini Kit v2.0 (QIAGEN, Germantown, MD, USA) on the EZ1 Advanced XL automated extraction platform. A total volume of 100 *µ*L of each processed sample was used according to the manufacturer’s instructions. Extracted RNA was used for real-time RT-PCR to determine Ct values and for conventional RT-PCR for targeted amplicon amplification.

### 2.4 Real-time RT-PCR for Ct determination

Real-time RT-PCR was performed to confirm the Ct value for each sample. The extracted RNA from all samples was screened using the same commercial subtype-differentiating assay described previously (Luqman et al., 2024): the RealPCR AMPV A/B Multiplex RNA Mix kit (IDEXX, Montpellier, France) for detection of AMPV/A and B RNA (IDEXX-99-56487). The manufacturer-designed primers and probes identify and differentiate AMPV/A and B. TaqMan™ Fast Virus 1-Step Master Mix (Applied Biosystems-4444434, Vilnius, Lithuania) was used for real-time RT-PCR. Each 20-*µ*L reaction contained 5 *µ*L extracted RNA, 5 *µ*L master mix, 5 *µ*L primer and probe kit, and 5 *µ*L RNase-free water. Cycling conditions were as follows: reverse transcription at 50°C for 15 min, initial denaturation at 95°C for 1 min, followed by 40 cycles of 95°C for 15 sec and 60°C for 30 sec. Reactions were performed on a QuantStudio 5 Real-Time PCR System (Thermo Fisher Scientific, Waltham, MA, USA).

### 2.5 Targeted amplicon sequencing

#### 2.5.1 Primer design

Multiplex primer panels were designed using PrimalScheme v3.3.0 (https://primalscheme.com) (Grubaugh et al., 2019). Reference genome sequences for aMPV subtypes A and B were obtained from GenBank (https://www.ncbi.nlm.nih.gov/genbank). Two primer pools (Pool 1 and Pool 2) were generated for each subtype based on the software-derived alignment. The average targeted amplicon length was approximately 1,100 bp for aMPV-A and 600 bp for aMPV-B. Primer sequences were synthesized by Integrated DNA Technologies (IDT, Coralville, IA, USA), and the subtype-specific primer names, pool assignments, coordinates, and sequences are provided in **Supplementary Table 2**.

#### 2.5.2 Amplicon amplification for aMPV subtype A

Primer Pool 1 and Pool 2 for the aMPV-A whole genome were used for amplicon amplification using the SuperScript IV One-Step RT-PCR System without ezDNase (Life Technologies, Carlsbad, CA, USA). Each reaction contained 12.5 *µ*L of 2× Platinum™ SuperFi™ RT-PCR Master Mix, 0.25 *µ*L of SuperScript™ IV RT Mix, 1.5 *µ*L of the respective primer pool, 8.25 *µ*L of UltraPure™ DNase/RNase-Free Distilled Water, and 2.5 *µ*L of template RNA. Amplification was performed on a SimpliAmp™ Thermal Cycler (Life Technologies Holdings Pte Ltd, Singapore) with the following conditions: reverse transcription at 50°C for 10 min, denaturation at 98°C for 2 min, followed by 40 cycles of 98°C for 10 sec (denaturation), 54°C for 10 sec (annealing), and 72°C for 30 sec (extension), with a final extension at 72°C for 5 min. Amplification products from each pool were confirmed by 1% agarose gel electrophoresis (expected size: ∼1,100 bp).

#### 2.5.3 Amplicon amplification for aMPV subtype B

The same reaction mixture and protocol used for aMPV-A were applied to aMPV-B, with a slight modification in the annealing temperature. The following cycling conditions were set: reverse transcription at 50°C for 10 min, denaturation at 98°C for 2 min, followed by 40 cycles of 98°C for 10 sec, 53°C for 10 sec (annealing), and 72°C for 30 sec (extension), with a final extension at 72°C for 5 min. Products from each pool were confirmed by 1% agarose gel electrophoresis (expected size: ∼600 bp).

#### 2.5.4 Amplicon purification and quality control

For both aMPV-A and aMPV-B, the amplified products from Pool 1 and Pool 2 were pooled and purified using AMPure XP Beads (Beckman Coulter, Inc., Brea, CA, USA). DNA was quantified using the Qubit 1× dsDNA HS (High Sensitivity) Assay Kit (Thermo Fisher Scientific, Eugene, OR, USA) on a Qubit 4 Fluorometer. Following library preparation, fragment size distribution and library quality were assessed on an Agilent TapeStation system using the appropriate ScreenTape assay (Agilent Technologies, Santa Clara, CA, USA) before pooling and sequencing.

### 2.6 Library preparation and sequencing

For each sample, purified subtype-specific amplicon pools were carried forward directly to library preparation. Indexed sequencing libraries were prepared with the Illumina DNA Prep (M) Tagmentation kit (Illumina, San Diego, CA, USA) according to the manufacturer’s instructions. Briefly, purified amplicons were subjected to tagmentation, followed by index incorporation and library amplification. Completed libraries were quantified, pooled, and loaded onto the Illumina MiSeq platform. Sequencing was performed using a MiSeq Reagent Kit v2 (300 cycles) to generate paired-end reads.

### 2.7 Computational processing pipeline

Raw sequencing reads were first assessed for quality using FastQC (Andrews, 2010). Adapter removal and quality trimming were then performed with Trim Galore (Krueger, 2026), which wraps Cutadapt (Martin, 2011), after which trimmed reads were assembled *de novo* using MEGAHIT (Li et al., 2015). The resulting contigs were compared against NCBI viral reference sequences using BLAST+ blastn (Camacho et al., 2009) to identify subtype-matched aMPV assemblies and to support gene-level annotation. Downstream processing, sequence extraction, coordinate handling, and figure-ready summary-table generation were performed with custom Python scripts developed for this project. These scripts were also used to integrate assembly metrics with sample metadata and to generate primer- and amplicon-level coverage summaries across the tiled panels. For comparative phylogenetic and selection analyses, assemblies were retained only after post-assembly review showed broad and coherent genome-wide support across the tiled design, consistent subtype assignment, and overall agreement among breadth, depth, mapped-read, and continuity metrics. Because this was the first comparative application of two newly optimized subtype-specific TAS panels, we did not apply a single numeric retention threshold to all assemblies. Instead, assemblies were retained after combined review of breadth, depth, mapped-read support, continuity, and subtype concordance. Positions with insufficient support were retained as ambiguous bases in the consensus sequence, and genomes with excessive unresolved or discontinuous regions were not retained in the final 91-genome comparative set.

### 2.8 Metadata integration and assay-performance analysis

To summarize assay performance beyond simple recovery counts, per-sample sequencing outputs were merged with sample metadata into a master analysis table containing subtype, host species, state, sample type, vaccination history, Ct value, total reads, mapped reads, mapping rate, breadth coverage, and mean genome coverage. From these integrated records, sequencing-performance summaries were generated by subtype, host species, and sample category, and assemblies were reviewed for consistency of genome-wide support before comparative phylogenetic and selection analyses, yielding a final comparative set of 91 genomes. In parallel, detailed primer- and amplicon-level coverage tables were generated for all recovered genomes using the subtype-specific scheme coordinates and custom Python scripts. Coverage fractions were calculated as the proportion of bases covered across each primer or nominal amplicon interval, recognizing that adjacent tiled amplicons overlap and can therefore preserve consensus sequence across neighboring regions even when support is not perfectly uniform at every interval boundary. Aggregate summaries were used to describe interval-level coverage patterns across the tiled panels.

### 2.9 Phylogenetic analysis

Multiple sequence alignments were performed using MAFFT v7.505 (Katoh and Standley, 2013). Maximum likelihood (ML) phylogenetic trees were constructed using IQ-TREE v2.2.0 (Minh et al., 2020) with automatic model selection by ModelFinder (Kalyaanamoorthy et al., 2017) and 1,000 ultrafast bootstrap replicates (Hoang et al., 2018). Trees were visualized and annotated using FigTree v1.4.4 (Rambaut, 2018). Phylogenetic analyses were conducted separately for WGS and G gene sequences for both subtypes A and B. Reference sequences for global aMPV-A and aMPV-B strains, including vaccine strains and their progenitors, were retrieved from GenBank and included in the analyses. Pairwise nucleotide and amino acid identity matrices were computed to assess genetic distances among strains. Exploratory recombination screening was performed on subtype-specific whole-genome alignments using 3Seq (Boni et al., 2007) and HyPhy GARD (Kosakovsky Pond et al., 2006, 2020); candidate signals were reviewed against alignment quality and genome-coordinate context before interpretation.

### 2.10 Selection analysis

Selective pressures were evaluated using HyPhy (Kosakovsky Pond et al., 2020) on cleaned codon alignments and corresponding ML trees. For G-gene site-wise analyses, subtype-specific G-gene alignments were processed by removing terminal stop codons, trimming sequences to codon-complete length, truncating internal stop codons when present, and sanitizing sequence and tree labels to ensure exact matching between alignments and phylogenies. Single-Likelihood Ancestor Counting (SLAC) and Fixed Effects Likelihood (FEL) (Kosakovsky Pond and Frost, 2005), together with Mixed Effects Model of Evolution (MEME) (Murrell et al., 2012), were then run on all branches of the subtype A and subtype BG-gene trees. For gene-wise comparison, aligned WGS datasets were partitioned into N, P, M, F, M2, SH, G, and L coding regions using subtype-specific reference coordinates, and SLAC was used to estimate global dN/dS ratios for each gene. BUSTED (Murrell et al., 2015) was used to test gene-wide episodic diversifying selection. In the site-wise summaries, codons with p values *<* 0.1 in FEL or MEME were considered candidate selected sites, whereas genes with BUSTED p values *<* 0.05 were considered to show evidence of episodic diversifying selection.

### 2.11 Statistical analysis of genome recovery

Subtype-specific genome-recovery analyses were performed from the updated supplementary workbook using Python with pandas v3.0.0, numpy v2.4.2, scipy v1.17.0, and openpyxl v3.1.5. For each subtype, Ct values in recovered and non-recovered samples were compared using the Mann–Whitney U test, which was selected because group sizes were unequal and Ct distributions were not assumed to be normally distributed. Simple subtype-specific logistic models were fit to estimate the relationship between Ct and probability of successful recovery. To provide an empirical summary of assay performance across the observed Ct range, recovery rates were also tabulated within Ct bins of ≤25, 25–30, and 30–35. Among successfully recovered genomes, Spearman correlation was used to assess the relationship of Ct with mean coverage and mapped reads.

## 3 Results

### 3.1 Whole-genome recovery and sequencing performance

Using the TAS workflow optimized in this study, 91 genomes were retained for comparative analyses from the 104 subtype-positive clinical samples, comprising 44 aMPV-A and 47 aMPV-B sequences. The final subtype A set comprised 42 turkey and 2 chicken genomes from North Carolina (n = 35), Missouri (n = 5), South Dakota (n = 2), Minnesota (n = 1), and Ohio (n = 1). The final subtype B set comprised 37 turkey and 10 chicken genomes from North Carolina (n = 35), Virginia (n = 3), Minnesota (n = 2), Pennsylvania (n = 2), South Carolina (n = 2), Missouri (n = 1), South Dakota (n = 1), and North Dakota (n = 1). A summary of the whole-genome sequencing results is provided in **Table 1**.

**Figure 2:**
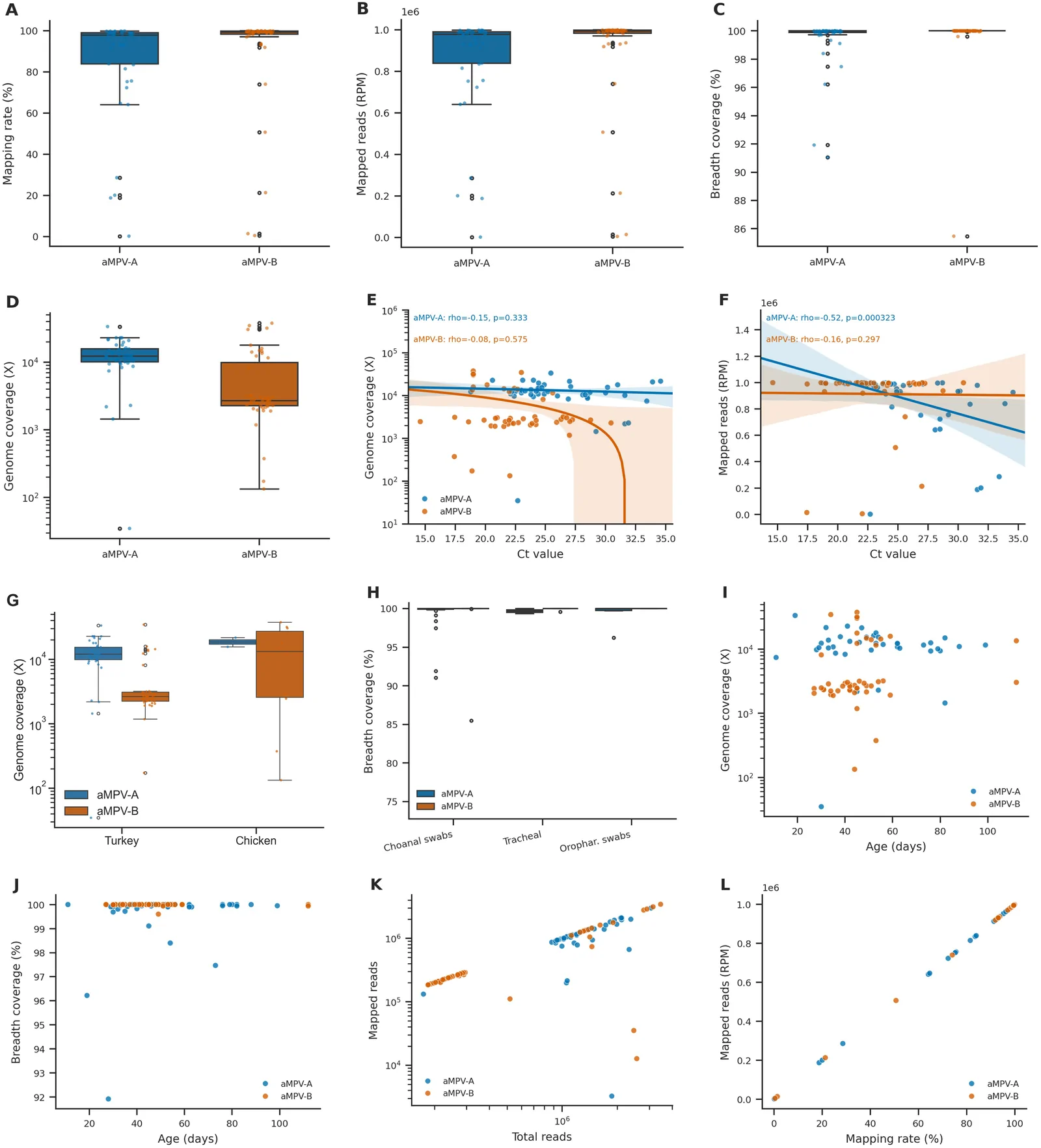
Integrated sequencing-performance summary across the analyzed aMPV genome dataset. **(A)** shows mapping rate by subtype. **(B)** shows RPM-normalized mapped reads by subtype. **(C)** shows breadth coverage by subtype. **(D)** shows mean genome coverage by subtype. **(E)** shows the relationship between Ct value and genome coverage. **(F)** shows the relationship between Ct value and RPM-normalized mapped reads. **(G)** shows genome coverage stratified by host species and subtype. **(H)** shows breadth coverage stratified by sample type and subtype. **(I)** shows the relationship between bird age and genome coverage. **(J)** shows the relationship between bird age and breadth coverage. **(K)** compares total reads and mapped reads. **(L)** shows the relationship between mapping rate and RPM-normalized mapped reads. Colored points indicate individual recovered genomes, and open circles denote boxplot outliers where applicable. These panels summarize TAS performance and highlight the metrics most sensitive to Ct and sample-to-sample variation. **(A)** aMPV subtype A G-gene tree **(B)** aMPV subtype B G-gene tree.

Successful recovery extended to samples with real-time RT-PCR Ct values up to 34.57 for aMPV-A and 31.2 for aMPV-B, demonstrating that the optimized TAS workflow could generate genomes suitable for comparative analysis from relatively low-viral-load clinical specimens. Within the final analysis set, subtype A genomes had a median of 1.11 million mapped reads, a median mapping rate of 97.8%, a median breadth coverage of 99.95%, and a median depth of 12,289.7×. The corresponding subtype B medians were 255,876 mapped reads, 99.4% mapping rate, 100% breadth coverage, and 2,681.5× depth. Thus, both assays routinely achieved broad genome coverage, whereas subtype A libraries generally produced deeper overall coverage. A combined 12-panel overview of mapping rate, normalized mapped reads, breadth, mean depth, and their relationships with Ct and metadata strata is shown in **Figure 2** (Panels A–L; subtype contrasts in Panels A–D, Ct relationships in Panels E–F, host and sample-type stratification in Panels G–H, and age/readout summaries in Panels I–L), and detailed sample-level metrics are provided in **Supplementary Table 1**. Approximately seven clinical submissions were positive for both aMPV-A and aMPV-B during diagnostic screening, with Ct values spanning approximately 19–30. One dual-positive submission was sequenced with both subtype-specific TAS panels, and both aMPV-A and aMPV-B genomes were recovered from the same clinical sample, supporting the ability of the subtype-specific workflow to resolve mixed-subtype submissions when viral loads are sufficient.

When genome recovery success was evaluated across the full 104-sample screening cohort, subtype-specific analysis showed the same overall decline in success as Ct increased. In the subtype A dataset, recovered samples had substantially lower Ct values than non-recovered samples (median 24.9 [IQR 23.68–28.55] versus 32.9 [31.1–34.15]; Mann–Whitney p = 0.00017). The corresponding subtype B comparison showed the same trend (22.17 [20.48–24.8] versus 30.4 [29.85–30.8]; p = 0.00717). Simple subtype-specific logistic models yielded strong discrimination between recovered and non-recovered samples (area under the receiver operating characteristic curve [AUC] 0.923 for aMPV-A and 0.969 for aMPV-B), with model-predicted 50% recovery occurring at Ct 32.9 for subtype A and 30.5 for subtype B. Empirically, all subtype A samples below Ct 30 were recovered, whereas success in the 30–35 range declined to 50.0% (8/16). For subtype B, recovery was 100% (37/37) at Ct ≤25, 90.9% (10/11) at Ct 25–30, and 50.0% (2/4) at Ct 30–35. Among recovered genomes, Ct showed only weak negative correlations with genome depth and breadth in both subtypes, whereas the strongest inverse association was observed between Ct and mapping-derived metrics in subtype A (Spearman *ρ* = −0.517 for both mapping rate and RPM-normalized mapped reads; p = 0.000323). Exploratory pooled penalized logistic models likewise identified Ct as the dominant predictor of recovery (AUC 0.945 with Ct alone), whereas adding subtype provided only a modest improvement (AUC 0.948) and further addition of host or tissue indicators increased AUC only marginally to 0.951. Because all non-recovered samples occurred among turkey swab submissions, these expanded models are best interpreted as descriptive summaries rather than formal inferential models. Recovery was broadly similar between North Carolina and non-North Carolina submissions overall (86.4%, 70/81, versus 91.3%, 21/23). By subtype, recovery was 81.4% (35/43) for North Carolina aMPV-A samples versus 100% (9/9) outside North Carolina, and 92.1% (35/38) for North Carolina aMPV-B samples versus 85.7% (12/14) outside North Carolina; because the non-North Carolina groups were small, these geographic comparisons are descriptive rather than inferential. Subtype-specific Ct-versus-recovery summaries are shown in **Supplementary Figure 1**, and the pooled recovery-model comparison is shown in **Supplementary Figure 2**. The full distributions of mapping rate, normalized mapped reads, breadth coverage, mean depth, and their relationships with Ct, host, sample type, and bird age are summarized in **Figure 2**, including host-species stratification in Panel G and sample-type stratification in Panel H.

Primer- and amplicon-level summaries showed broad representation of the tiled panels across both subtype datasets once genomes were recovered, together with modest and reproducible interval-level variation in coverage support. Because adjacent tiled amplicons overlap, these summaries should be interpreted as measures of local redundancy across nominal intervals rather than as all-or-none indicators of genome recovery. Across both subtypes, all designed amplicons contributed sequence in the recovered dataset, and the principal pattern was that a small subset of interval ends showed less uniform support than the rest of the panel. These effects were local rather than genome wide: adjacent amplicons typically bridged the same regions, so consensus breadth was largely maintained even where strict full-interval coverage varied. The corresponding primer- and amplicon-level summaries are provided in **Supplementary Table 3**.

The integrated performance table also identified a small number of recovered genomes with atypical mapping behavior relative to their Ct values. For example, the subtype A vaccine-derived genome ADRDL-43 was recovered with a Ct of 22.7 but only 3,245 mapped reads, a mapping rate of 0.17%, and a mean depth of 34.85×, whereas several subtype B genomes retained complete breadth despite markedly reduced mapped-read counts, including ADRDL-11 and ADRDL-12. These observations indicate that Ct is the principal determinant of overall recovery success, but residual variation among recovered genomes is shaped more by sample-specific mapping efficiency and local coverage distribution than by Ct alone.

### 3.2 Phylogenetic analysis of aMPV subtype A

**Figure 3:**
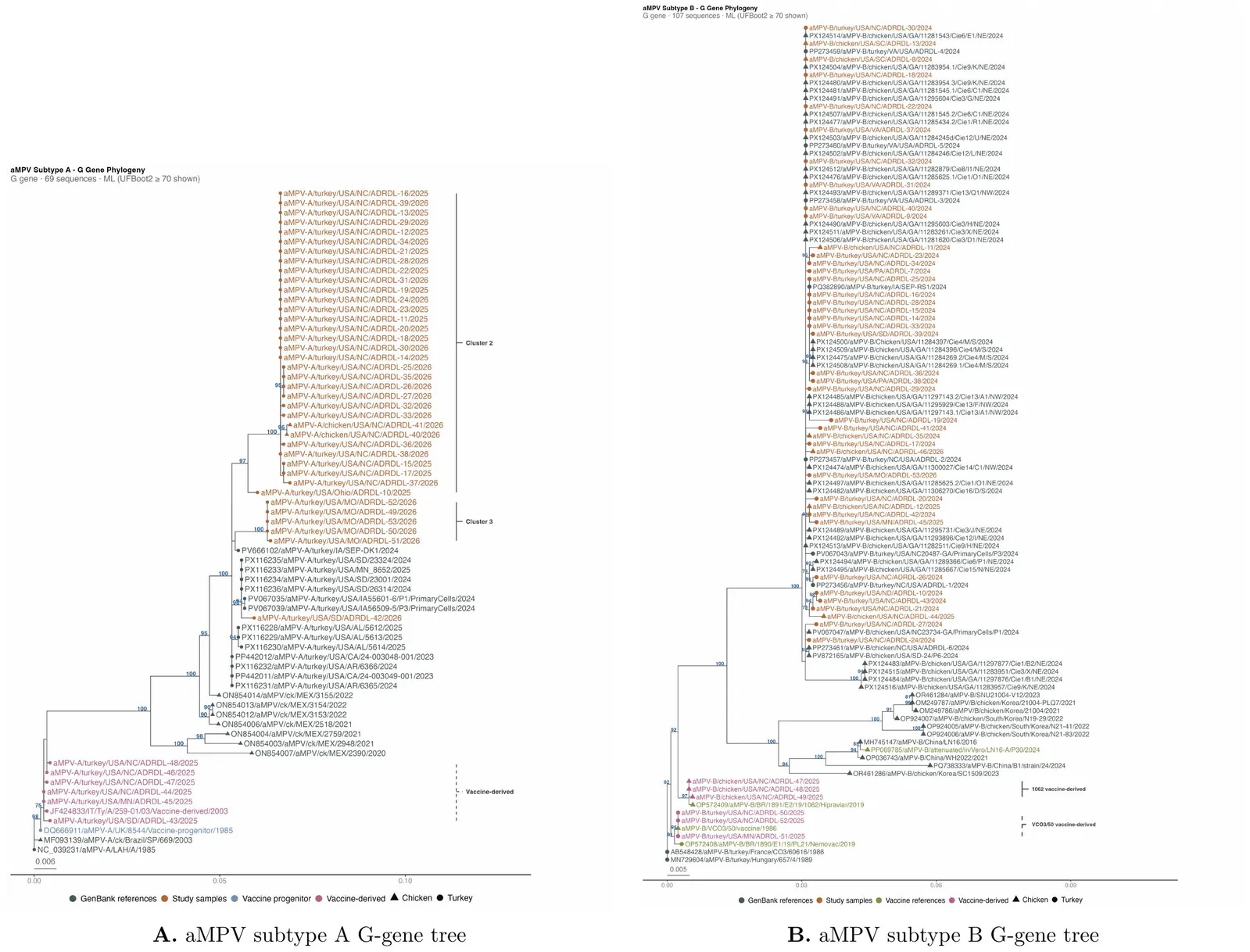
Maximum-likelihood phylogenetic trees based on G-gene sequences of aMPV subtype A and subtype B strains. Both trees show the relationships among US study samples, GenBank references, and vaccine-derived lineages. In the subtype A tree, only Cluster 2 and Cluster 3 are annotated for visual clarity within the broader US lineage, whereas the subtype B tree shows the close relatedness of US field strains together with the separate placement of vaccine-derived lineages. Bootstrap support values greater than 70% are shown at branch nodes. **(A)** aMPV subtype A WGS tree **(B)** aMPV subtype B WGS tree.

**Figure 4:**
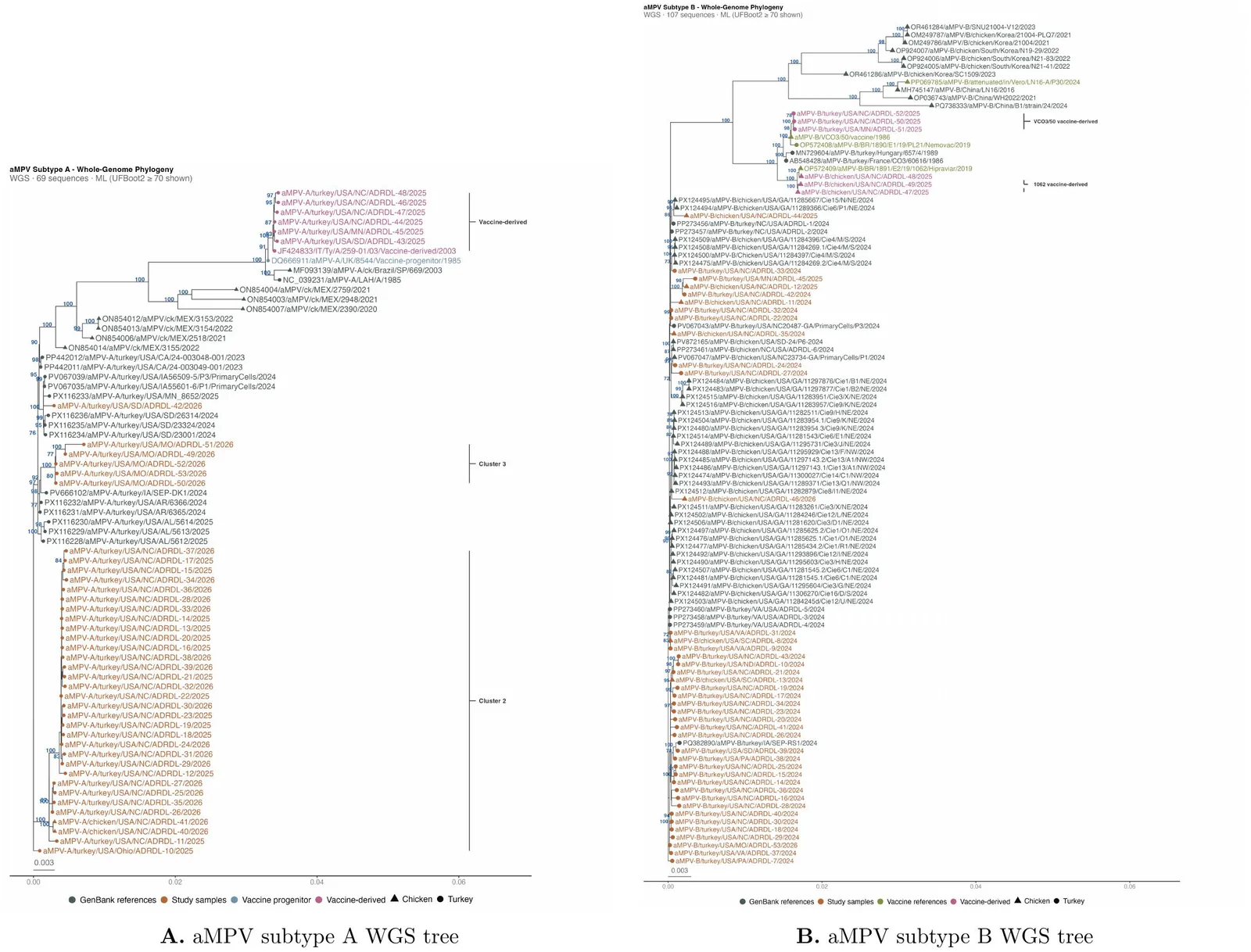
Whole-genome sequence-based phylogenetic trees of aMPV subtype **(A)** and subtype **(B)** strains. The WGS analyses resolve Cluster 2 and Cluster 3 within the broader subtype **(A)** US lineage, confirm the close relatedness of US subtype **(B)** field strains across states and host species, and distinguish field-derived from vaccine-derived lineages using complete-genome sequence context.

Since the introduction of aMPV-A into US poultry, the emerging strains have formed a distinct monophyletic cluster within group IV, closely related to contemporary Mexican isolates. The earliest US strains were detected in California (two strains in late 2023) and Arkansas (two strains in early 2024). These initial isolates were highly similar by WGS analysis, with no detectable mutations in the G gene. Subsequently, through mid-2025, all aMPV-A strains detected in US poultry from Iowa (3 strains), South Dakota (3 strains), Minnesota (1 strain), and Alabama (3 strains) remained highly similar, clustering with more than 99.5% nucleotide identity by both WGS and G-gene analyses and displaying only 1–3 amino acid substitutions in the G protein relative to the earliest California and Arkansas strains (**Figures 3**, **4**). The corresponding aMPV-A phylogenies for the remaining individual coding regions are provided in **Supplementary Figure 3**.

#### 3.2.1 Emergence of aMPV-A cluster 2

In North Carolina, aMPV-B was the dominant circulating subtype until mid-2025, when aMPV-A was first detected. By the end of May 2025, sequencing of the first recovered North Carolina aMPV-A-positive clinical sample from a turkey farm (aMPV-A/ADRDL-11) revealed a striking cluster of nonsynonymous substitutions concentrated within residues 209–275 of the G protein ectodomain, compared with all previously detected US aMPV-A strains. This observation raised concern that a new subtype A group had emerged in North Carolina poultry. Additional North Carolina subtype A submissions accumulated during the following winter outbreak and repeatedly reproduced the same mutational pattern, confirming that the initial genome did not represent an isolated anomaly.

During the winter of 2025–2026, a severe aMPV-A outbreak affected North Carolina turkey farms and prompted submission of approximately 36 additional clinical samples from affected farms. To our knowledge, this represents the first published genomic documentation of aMPV-A detection in North Carolina poultry. Using TAS, we recovered 28 WGSs from these submissions (aMPV-A/ADRDL-12 through ADRDL-39). To assess the breadth of spread within the same production region, we also sequenced two aMPV-A-positive chicken samples from North Carolina (aMPV-A/ADRDL-40 and ADRDL-41), both of which clustered with the same group. The North Carolina turkey and chicken genomes, together with the earlier Ohio genome ADRDL-10, formed a strongly supported distinct subcluster within the broader US group IV lineage by both G-gene and WGS analyses; this group is referred to here as Cluster 2 and shares approximately 98.5% nucleotide identity and 97.1% amino acid identity in the G gene with the earlier US Cluster 1 strains (**Figures 3**, **4**). In total, Cluster 2 was detected on 30 turkey and chicken farms in North Carolina during the study period. The recovery of closely related genomes from both turkeys and chickens in the same production area is consistent with local cross-species circulation where the two host populations are raised in proximity. The affected North Carolina farms had received subtype B vaccination, showing that this subtype A group was detected on farms using heterologous subtype B vaccination; however, the present dataset does not allow vaccine performance or protection to be inferred directly.

The mutational profile of the North Carolina Cluster 2 outbreak genomes was dominated by G-gene change, including 10–11 nonsynonymous amino acid substitutions concentrated within a ∼65 amino acid region of the ectodomain. This group included multiple serine-to-proline and leucine-to-proline substitutions, consistent with a marked local shift in amino acid composition. At the nucleotide level, 15–18 substitutions were observed across North Carolina Cluster 2 G genes, most of them T-to-C transitions, and these yielded a substantially higher fraction of amino acid-changing mutations than was seen in the longer internal genes. The earlier Ohio Cluster 2 genome was less diverged, with 8 nucleotide and 6 amino acid differences in G relative to the first-year Cluster 1 consensus. By contrast, the L gene accumulated 12 nucleotide substitutions but only two amino acid changes despite its much greater length, while only isolated amino acid changes were observed in N, P, F, and SH. Outside North Carolina, additional evidence for ongoing subtype A diversification was seen in South Dakota, where a later strain detected in early 2026 (aMPV-A/ADRDL-42) carried further G-protein substitutions relative to earlier upper Midwest strains despite homologous subtype A vaccination. A condensed amino-acid alignment of this 209–275 ectodomain segment, comparing Cluster 2 and Cluster 3 patterns against the Cluster 1 consensus, is provided in **Supplementary Figure 4**.

#### 3.2.2 Emergence of aMPV-A cluster 3

A second subtype A field group, designated Cluster 3, was also recovered from five Missouri turkey submissions collected in March 2026 (ADRDL-49 through ADRDL-53). These genomes grouped together as a compact Missouri lineage distinct from both Cluster 1 and Cluster 2 by both G-gene and WGS analyses. Within the G gene, Cluster 3 strains remained highly similar to one another but shared approximately 98.9% nucleotide identity and 97.5% amino acid identity with Cluster 1 and 98.1% nucleotide identity and 96.1% amino acid identity with Cluster 2. Relative to the first-year Cluster 1 strains, the Missouri genomes carried nine amino acid substitutions. Five of these were leucine-to-proline or serine-to-proline changes within the same 209–275 ectodomain segment altered in Cluster 2, two substitutions (L248P and L272P) were shared with Cluster 2, and Y275H also mapped within this same region. An additional L-to-P substitution occurred at residue 292, whereas V147I and R345G lay outside this concentrated segment. These findings indicate that subtype A diversification in US poultry is no longer restricted to a single focal outbreak and that the Missouri Cluster 3 lineage has acquired a partially overlapping but distinct G-protein mutational profile.

### 3.3 Phylogenetic analysis of aMPV subtype B

#### 3.3.1 US aMPV-B field strains

Phylogenetic analysis of the final aMPV-B dataset identified 41 field-strain genomes from chickens and turkeys across North Carolina, Virginia, Minnesota, Pennsylvania, North Dakota, South Carolina, South Dakota, and Missouri. These strains, collected over two years from early 2024 to early 2026, aligned closely with previously detected US strains, including our earlier isolates (aMPV-B/ADRDL-1–6), the chicken isolate SD-24/P6, 42 strains from Georgia, two isolates from North Carolina, and one from Iowa. All US aMPV-B field strains exhibited high genetic homogeneity, forming a distinct monophyletic group with >99% nucleotide identity in both WGS and G gene analyses, irrespective of host species (chicken or turkey) or geographic origin (**Figures 3**, **4**). Detailed analysis of the G protein revealed 1–6 amino acid substitutions across all US field strains, with no evidence of extensive mutational divergence. Four divergent strains from Georgia chicken farms (PX124483, PX124484, PX124515, and PX124516) (Alvarez-Narvaez et al., 2025), while harboring 16 T-to-C nucleotide substitutions in the G gene, shared 98.6% nucleotide identity with all other US aMPV-B strains. Most of these substitutions were synonymous, yielding only 5 nonsynonymous changes at the protein level (31.3%), in contrast to the 71.4% nonsynonymous rate observed for aMPV-A Cluster 2. Some of the assembled US-field aMPV-B strains were detected on vaccinated farms at 4–5 weeks post-vaccination. The corresponding aMPV-B phylogenies for the remaining individual coding regions are provided in **Supplementary Figure 5**.

### 3.4 Detection and characterization of vaccine-derived strains

#### 3.4.1 aMPV-A vaccine-derived strains

In parallel with the North Carolina Cluster 2 field lineage, we identified a separate aMPV-A lineage that phylogenetically aligned with the commercial subtype A vaccine strain and its progenitor rather than with the US field-strain clade (**Figure 4**). Six aMPV-A vaccine-derived strains were detected on turkey farms and carried the characteristic vaccine-related signature previously reported for the commercial subtype A vaccine lineage (Selim et al., 2026b). Two of these strains were detected on vaccinated farms in South Dakota and Minnesota, where birds had received the imported subtype A vaccine by hatchery spray and were sampled during respiratory disease episodes with elevated mortality. One of these genomes (ADRDL-43) was detected at 30 days of age with a Ct value of 22.7, whereas ADRDL-45 was detected at 19 days of age with a Ct value of 25.45. The remaining four vaccine-derived strains were detected on nonvaccinated turkey farms in North Carolina that had received only subtype B vaccination; those farms experienced mild to severe respiratory signs with choanal-cleft-swab Ct values ranging from 23.8 to 30, consistent with environmental spread or local circulation of the subtype A vaccine virus in an area where the subtype A vaccine had not been uniformly applied (Catelli et al., 2006; Lupini et al., 2011). Across all six subtype A vaccine-derived genomes, additional mutations were present beyond the vaccine-defining markers, including reversions toward the vaccine progenitor at selected positions across all six genomes, indicating repeated in-field passage rather than simple contamination with unpassaged vaccine. Paper-defined subtype A vaccine-lineage marker positions and vaccine progenitor comparisons for these genomes are summarized in **Supplementary Table 4** and visualized by genome region in **Supplementary Figure 6**.

#### 3.4.2 aMPV-B vaccine-derived strains

Six aMPV-B vaccine-derived strains were identified: three derived from the 1062 vaccine strain (aMPV-B/ADRDL-49–51) and three from the VCO3/50 vaccine strain (aMPV-B/ADRDL-52–54).

The three 1062-derived strains were detected on North Carolina broiler farms at approximately 45 days of age. These birds had been vaccinated at one day of age with a different subtype B vaccine strain (PL21) by hatchery spray together with infectious bronchitis virus and Newcastle disease virus vaccines. The affected flocks showed respiratory signs, low Ct values (19–21), and no evidence of concurrent IBV infection; two of the cases were associated with marked swollen-head syndrome and mortality approaching 10%. Phylogenetic analysis confirmed that these genomes retained the expected 1062 vaccine-like genomic background, showed approximately 99.9% nucleotide identity to that vaccine, and carried additional mutations consistent with onward passage after administration.

The three VCO3/50-derived strains were detected on turkey farms (two in North Carolina and one in Minnesota) that had been vaccinated at one day of age with the homologous VCO3/50 vaccine. These strains also displayed 99.9% nucleotide identity with one another and clustered closely with the VCO3/50 vaccine strain (**Figure 4**). They were detected at 2–3 weeks of age in flocks showing respiratory disease and variable mortality. All three VCO3/50-derived genomes contained the expected VCO3/50 vaccine-like genomic background, including the SH stop codon that truncates the protein at amino acid position 133 (Sugiyama et al., 2010). Shared post-vaccination mutations were observed in P, F, and SH, whereas an additional distinguishing mutation in L was unique to each genome. Lineage-specific differences relative to the subtype B vaccine references are summarized in **Supplementary Table 4**.

All vaccine-derived strains in this study were detected in diseased flocks with respiratory signs and high viral loads (Ct values as low as 19). In addition to the shared vaccine-defining markers, each recovered vaccine-derived genome carried one or more lineage-specific substitutions relative to its corresponding vaccine reference, and some genomes also showed reversions toward the vaccine progenitor at selected positions. These findings are more consistent with repeated in-field passage and divergence after vaccine use than with simple contamination by unpassaged vaccine material.

### 3.5 Selection analysis supports concentrated evolutionary pressure on G, SH, and M2

**Figure 5:**
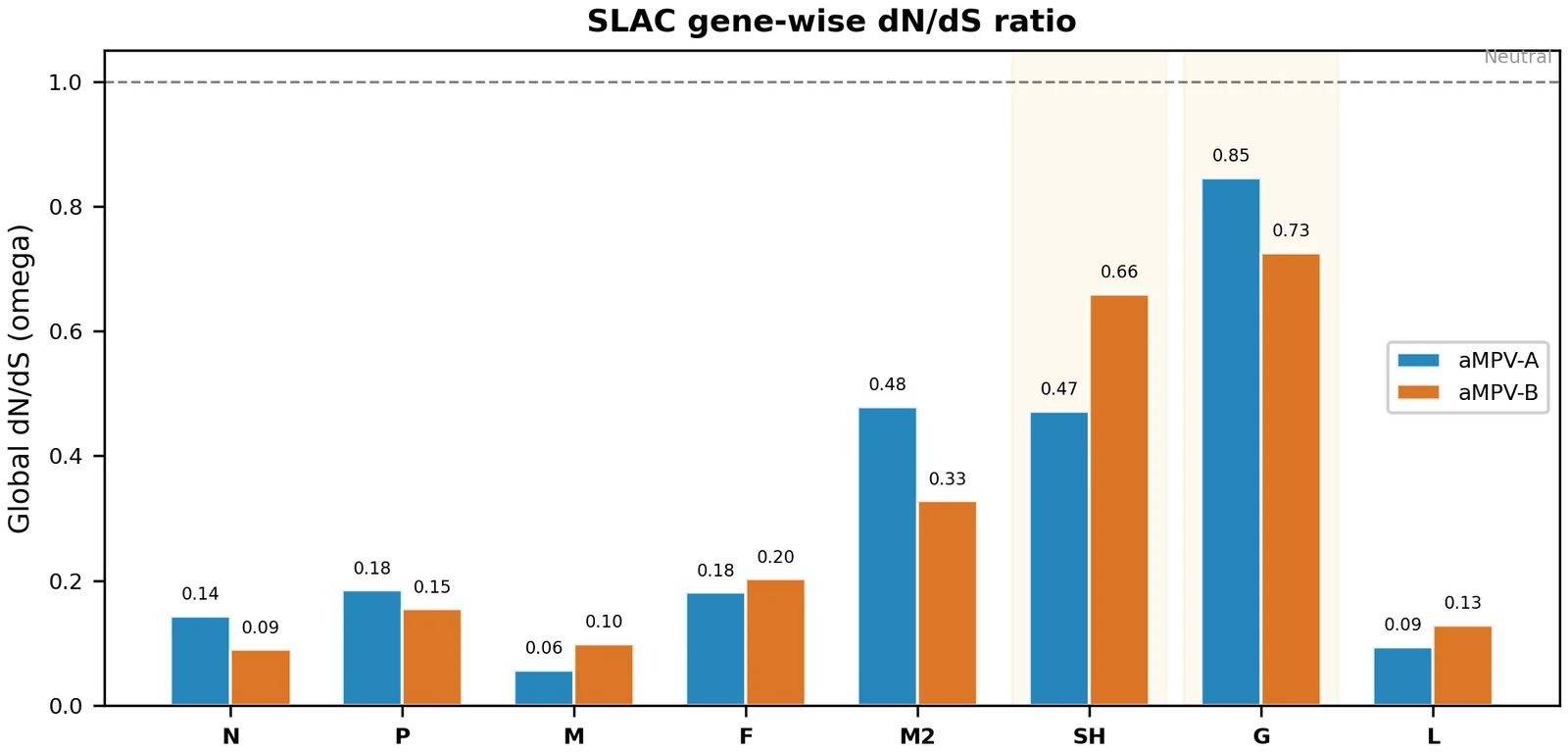
Gene-wise global dN/dS estimates obtained by SLAC for the coding regions of aMPV subtypes A and B. All genes remained below neutrality (dN/dS *<* 1), indicating predominant purifying selection across the genome. However, the highest relative omega values were observed in the G gene in both subtypes, followed by SH and M2, supporting concentration of evolutionary pressure in envelope-associated genes. Additional site-wise FEL and MEME analyses of the G gene and gene-wide BUSTED summaries are provided in **Supplementary Figures 7**-**S9**.

Gene-wise SLAC estimates showed that all coding regions remained below the neutral expectation of dN/dS = 1, indicating that purifying selection dominated overall genome evolution in both subtypes. Nevertheless, the highest relative dN/dS values were observed in the G gene for both subtypes (0.85 in aMPV-A and 0.72 in aMPV-B), followed by SH (0.47 and 0.66, respectively) and M2 (0.48 and 0.33, respectively), whereas the remaining genes showed substantially lower values (**Figure 5**). These results are consistent with disproportionate sequence change in envelope-associated genes relative to internal structural and polymerase genes.

Site-wise G-gene analyses further refined this pattern. FEL identified two positively selected and 15 purifying-selected codons in subtype A and four positively selected and 20 purifying-selected codons in subtype B (p *<* 0.1). Importantly, both positively selected subtype A codons occurred within the 209–275 Cluster 2 hotspot, whereas no comparable hotspot concentration was observed in subtype B. MEME identified six codons under episodic diversifying selection in subtype A and seven in subtype B, with two subtype A codons and one subtype B codon falling within the same hotspot region. At the gene-wide level, BUSTED detected significant episodic diversifying selection in subtype A N, M, F, and G genes and in subtype B M, M2, and G genes. Taken together, these analyses indicate that although purifying selection predominates across the aMPV genome, the G gene, and to a lesser extent SH and M2, shows the clearest evidence of relaxed constraint or episodic diversification. Additional site-wise FEL summaries are shown in **Supplementary Figure 7**, MEME summaries in **Supplementary Figure 8**, and gene-wise BUSTED summaries in **Supplementary Figure 9**.

## 4 Discussion

Following the emergence of aMPV subtypes A and B on US poultry farms in early 2024, infection spread rapidly through major poultry-producing regions and caused substantial economic losses. In response, the USDA Center for Veterinary Biologics (CVB) implemented emergency regulatory measures and issued permits to facilitate access to aMPV vaccines while domestic products were being developed (USDA-APHIS, 2024). In the present study, the optimized TAS workflow retained 91 genomes for comparative phylogenetic and selection analyses, including successful subtype A recovery at Ct values up to 34.57 and subtype B recovery up to 31.2. These empirical limits extend the practical range of direct-from-sample aMPV sequencing and show that broad genome recovery is achievable even from specimens that would be challenging for untargeted metagenomic approaches.

The TAS workflow optimized in this study provides a practical and cost-effective approach for characterizing aMPV epidemiology and genetic diversity. Recent subtype B work showed that an amplicon-based tiled PCR approach can enrich emerging aMPV-B genomes before next-generation sequencing (Alvarez-Narvaez et al., 2026); the present study extends that concept by applying subtype-specific tiled panels to both aMPV-A and aMPV-B clinical submissions and by integrating recovery, coverage, phylogenetic, vaccine-lineage, and selection analyses in a single surveillance framework. This addresses a longstanding limitation in many molecular epidemiological studies of aMPVs, which have historically relied on partial G-gene datasets rather than genome-scale data to investigate viral spread and evolution (Franzo et al., 2020; Mescolini et al., 2021). This reliance on partial sequences is reflected in the relatively limited number of whole-genome aMPV sequences available in GenBank compared with the far more abundant partial G gene sequences, constraining broader understanding of aMPV genomic epidemiology, genetic variability, and evolution.

The integrated assay-performance analyses add practical context to that conclusion. Across recovered genomes, mapping rates and breadth coverage were generally high for both subtypes, indicating that the tiled TAS strategy was robust once amplification succeeded. Coverage summaries further showed that support was distributed broadly across the targeted genomes, with the expected interval-to-interval variability of an overlapping tiled design. Because neighboring amplicons provide redundant support, modest local differences in interval coverage are compatible with stable consensus recovery across the same regions. Thus, the value of the tiled design was not simply that it increased viral sequence yield, but that it provided overlapping local support that allowed genome-wide consensus recovery despite uneven interval-level performance. Framed this way, the coverage analysis complements the high recovery rate by explaining why some otherwise successful genomes differed modestly in mapped-read efficiency or local coverage profile while still preserving broad genomic breadth.

Genetic variability in aMPVs is predominantly concentrated in the G gene, where mutations are predominantly nonsynonymous and frequently affect charged residues or serine/threonine sites associated with O-linked glycosylation, which may shield epitopes and facilitate immune evasion (Catelli et al., 2010; de Graaf et al., 2008). Multiple studies have demonstrated that the G gene is under stronger diversifying pressure than most other coding regions, supporting its role as a major antigenic determinant relative to more conserved loci such as F and N (Catelli et al., 2010; de Graaf et al., 2008). Comparative studies of field-derived datasets have likewise emphasized that the G protein accumulates substantially greater amino acid diversity than more conserved structural genes such as F (Catelli et al., 2010), including recent whole-genome analysis of contemporary US subtype B isolates (Alvarez-Narvaez et al., 2025). Our gene-wise SLAC analyses were consistent with that broader pattern: although no gene reached a global dN/dS ratio above 1, the G gene had the highest relative omega value in both subtypes, followed by SH and M2, indicating that the strongest evolutionary pressure was concentrated in the same loci highlighted by the phylogenetic and mutational analyses. A similar concentration of evolutionary pressure in G, SH, and M2 was reported for contemporary Georgia aMPV-B isolates (Alvarez-Narvaez et al., 2025).

The SH gene also appears to be significantly involved in aMPV evolution, paralleling the G gene in its importance. Although the exact function of the SH protein remains unknown, its surface localization alongside the G protein may make both proteins more accessible to host immunity and therefore more likely to experience immune-associated diversification (de Graaf et al., 2008). Prior field studies have also linked genetic divergence between circulating field strains and vaccine strains to reduced vaccine match, particularly when sequence change accumulates in surface glycoprotein genes (Catelli et al., 2010). In our dataset, SH consistently ranked among the genes with the highest gene-wise omega values, particularly in subtype B, while BUSTED also supported episodic diversifying selection in subtype B M2 and G. These results reinforce the interpretation that aMPV evolution is concentrated in a limited subset of genes rather than being uniformly distributed across the genome.

Subtype A Cluster 2, first recognized in North Carolina, is particularly notable. This group, detected on 30 turkey and chicken farms, was defined by a concentrated G-protein hotspot and, for the North Carolina outbreak genomes, harbored 10–11 nonsynonymous mutations in the G protein, a marked increase compared with the earlier US Cluster 1 strains detected during the first year of introduction, which showed greater than 99.6% nucleotide and amino acid identity within the G gene and only limited amino acid variation. Cluster 2 strains remained highly similar to one another across turkey and chicken hosts but shared only about 98.5% nucleotide identity and 97.1% amino acid identity in the G gene relative to Cluster 1. Their substitutions were concentrated within a ∼65 amino acid region (residues 209–275) of the G protein ectodomain and were substantially more numerous than those observed in other genes, including the L gene, which had only 2 amino acid changes despite encoding an approximately 4-fold longer nucleotide sequence.

The introduction of multiple proline substitutions (S→P, n = 3; L→P, n = 6) within this confined segment of the G protein ectodomain may affect local secondary structure and antigenic architecture. Proline, due to its cyclic side chain, imposes conformational rigidity on the polypeptide backbone and can introduce structural kinks in protein backbones (Morgan and Rubenstein, 2013). The clustering of several proline substitutions within a short region of the G ectodomain is therefore consistent with altered local conformation and potentially altered antigenic presentation, even though direct structural testing was beyond the scope of the present study. Missouri Cluster 3 strains show a similar pattern. Although Cluster 3 was recovered only from five turkey submissions, it remained internally cohesive while differing from Cluster 1 by nine G-protein amino acid substitutions and from Cluster 2 by an even lower G-protein amino acid identity (96.1%). Several Cluster 3 substitutions mapped to the same 209–275 segment altered in Cluster 2, including shared L248P and L272P changes, but Cluster 3 also contained unique substitutions such as Y275H, V147I, R345G, and an additional proline substitution at residue 292. These observations indicate that independent subtype A groups in the United States are accumulating change in overlapping regions of the same major surface glycoprotein, consistent with continued diversification after introduction. The heavy contribution of North Carolina submissions reflects diagnostic sampling density during a major regional outbreak rather than a structured prevalence survey, and the observed frequency of Cluster 2 in this dataset should not be interpreted as a formal estimate of statewide or national prevalence.

A notable prevalence of T-to-C transition mutations was observed in the G gene of both aMPV-A Cluster 2 and Cluster 3 and in the four divergent aMPV-B strains detected on Georgia chicken farms (Alvarez-Narvaez et al., 2025). This pattern aligns with the transition-biased single nucleotide polymorphism profile reported by Alvarez-Narvaez et al. for recent US aMPV-B genomes, in which substitutions within the same base class, including purine-to-purine and pyrimidine-to-pyrimidine changes, were overrepresented (Alvarez-Narvaez et al., 2025). Transition enrichment is also common in RNA-virus evolution and may reflect the combined effects of polymerase error, selection, and host-mediated RNA editing (Lyons and Lauring, 2017; Piontkivska et al., 2021). One biologically plausible source of excess U/T-to-C signal is adenosine deaminase acting on RNA (ADAR)-mediated A-to-I editing on double-stranded RNA replication intermediates; for negative-sense RNA viruses, editing of antigenomic templates can be detected in progeny genomes as U/T-to-C substitutions. ADAR-associated editing or hypermutation has been described in several RNA-virus systems (Suspene et al., 2008, 2011; Piontkivska et al., 2021). However, the present consensus-genome dataset cannot directly distinguish ADAR-linked editing from polymerase error, selective filtering, or other replication-associated processes. The repeated appearance of transition-rich G-gene change in divergent subtype A clusters and in divergent subtype B Georgia genomes should therefore be viewed as a hypothesis-generating pattern consistent with ongoing field replication and host-virus interaction, rather than as direct proof of a specific editing mechanism. The higher fraction of nonsynonymous substitutions in subtype A than in subtype B likewise suggests stronger diversifying pressure on the G protein in subtype A, particularly within the Cluster 2 hotspot, whereas subtype B appears to remain under stronger overall purifying constraint. This interpretation is supported by the site-wise analyses, which placed both positively selected subtype A FEL codons and two subtype A MEME codons within the 209–275 Cluster 2 hotspot, whereas subtype B showed a more diffuse pattern of candidate selected sites across the G gene. Recent sequencing of subtype B vaccine strains from Brazil also reported unusual C-to-T heterogeneity in the G gene of BR/1891/E2/19, indicating that transition-rich heterogeneity can occur in aMPV vaccine-related genomes as well (Kariithi et al., 2023).

TAS also distinguished field-derived from vaccine-derived aMPV genomes directly from clinical samples. In this dataset, vaccine-derived designation was supported by phylogenetic clustering with the vaccine backbone, retention of vaccine-defining markers, separation from the parental strain, and additional substitutions consistent with continued replication after vaccine administration rather than simple detection of unpassaged vaccine material (Catelli et al., 2006; Lupini et al., 2011; Selim et al., 2026b).

In addition to these genomic criteria, biological characterization of suspected vaccine-derived strains remains essential. The present data establish genomic relatedness and field detection, but they do not by themselves determine whether any post-vaccination substitutions alter attenuation, transmission, or tissue tropism. Direct-from-sample WGS therefore provides a starting point for future reverse-genetics or challenge studies aimed at testing the phenotypic significance of representative vaccine-derived genomes under controlled conditions.

We recovered the WGS of 12 vaccine-derived strains: six from aMPV-A vaccines, identified on turkey farms, and six from aMPV-B vaccines, detected on both chicken (n = 3) and turkey (n = 3) farms. The detection of vaccine-derived strains on both vaccinated and nonvaccinated farms is consistent with previous studies from Europe showing that live aMPV vaccine viruses can circulate beyond the flock in which they were first applied and may be detected on neighboring farms (Lupini et al., 2011, 2023). Our data extend those observations by showing, at whole-genome resolution, that the US vaccine-derived genomes retained the defining signatures of the imported vaccine strains while also accumulating additional lineage-specific substitutions after field use.

The field detection of genomes that cluster closely with licensed vaccines is a well-recognized feature of live aMPV vaccination programs (Cecchinato et al., 2014; Catelli et al., 2006; Franzo et al., 2020). However, many earlier molecular epidemiology studies and surveillance reconstructions were based primarily on partial G-gene datasets, which limited resolution of how closely vaccine-like detections matched the vaccine backbone across the rest of the genome (Franzo et al., 2020; Mescolini et al., 2021). In the present dataset, WGS improves confidence in distinguishing vaccine-derived genomes from circulating field strains.

Although several mechanisms have been proposed to explain the emergence of vaccine-derived aMPV strains, including vaccine strain subpopulations (Naylor and Jones, 1994) and uneven field application (Catelli et al., 2006), the present data are compatible with continued post-vaccination replication and short-range circulation under field conditions. Under farm conditions, vaccine-like viruses can be detected beyond the immediate post-vaccination window, particularly when mass application by spray or drinking water produces uneven flock-level exposure and facilitates bird-to-bird passage (Catelli et al., 2006; Cecchinato et al., 2010; Lupini et al., 2011, 2023). Such rolling replication provides an opportunity for vaccine virus to pass among birds, persist longer than expected, and accumulate additional substitutions. In our dataset, vaccine-derived detections clustered around 30–40 days post-vaccination, a pattern that is more compatible with continued field replication than with simple residual detection immediately after vaccine administration. From an epidemiological perspective, this reinforces the importance of consistent vaccine delivery and WGS-based review of vaccine-like genomes detected in clinical submissions.

The first year of vaccine use in US poultry also yielded several observations that should be framed within the limitations of the available field metadata. This study documents aMPV-A detection in North Carolina turkeys and chickens from a region where subtype B outbreaks had predominated in our diagnostic submissions. Importantly, the six subtype A vaccine-derived genomes were related to the commercial subtype A vaccine lineage rather than to the US subtype A field-strain lineage. These data should not be interpreted as a direct measure of homologous or heterologous vaccine protection, because vaccine timing, administration, flock coverage, and field exposure could not be evaluated in this study. Instead, the main implication is that vaccine-derived aMPV lineages can be detected in clinical submissions after field use of live vaccines, and that WGS-based surveillance is needed to distinguish these genomes from independently circulating field strains. Formal conclusions about vaccine efficacy, transmission fitness, or pathogenicity will require longitudinal field studies and controlled challenge experiments.

We acknowledge several limitations of this study. First, although we incorporated gene-wise and site-wise selection analyses, these results are exploratory because they were derived from consensus sequences with uneven temporal and geographic sampling and without functional validation of individual mutations. Second, although we performed subtype-specific Ct-based recovery modeling, the number of non-recovered samples was limited, particularly for subtype B, and therefore more complex multivariable models incorporating subtype, sample type, host, and submission period will require larger datasets. Third, the biological consequences of the additional substitutions observed in vaccine-derived genomes remain unknown and will require challenge studies or reverse-genetics approaches for direct phenotypic testing. Fourth, although subtype-specific TAS recovered both aMPV-A and aMPV-B genomes from one dual-positive clinical submission, consensus genome reconstruction from direct clinical samples was not designed to resolve minor within-subtype subpopulations; mixed-subtype results were therefore interpreted at the subtype level, and minor-strain composition would require isolate-level or deep variant analysis. Fifth, exploratory recombination screening did not provide robust evidence of biological recombination among the sequenced aMPV field strains, and no recombination event was supported or used for lineage interpretation. Continued monitoring for recombination will remain important as field and vaccine-derived lineages co-circulate. Sixth, the detection of the most divergent field strains and the novel aMPV-A clusters in a limited number of focal geographic regions may partly reflect sampling density, and broader surveillance across additional states and production systems will be needed to define their full distribution and evolutionary trajectory.

**Table 1:** Summary of whole-genome sequencing results for aMPV subtypes A and B from US poultry clinical samples.

Parameter	aMPV-A	aMPV-B
Total subtype-positive clinical samples analyzed	104	
Genomes included in comparative analyses	44	47
Final comparative dataset	87.5% (91/104)	
Turkey samples	42	37
Chicken samples	2	10
Maximum Ct for successful assembly	34.57	31.2
Recommended Ct cutoff	≤34	≤31
Number of US states represented	5	8
Field strains detected	38	41
Vaccine-derived strains detected	6	6

## 5 Conclusions

In conclusion, we developed and optimized targeted amplicon sequencing assays for aMPV subtypes A and B and applied them to 104 subtype-positive clinical samples collected across nine US states. The assays enabled direct whole-genome sequencing from clinical samples across a broad Ct range and supported recovery of 91 genomes for comparative analysis. This study documents divergent US field aMPV-A clusters, including Cluster 2 in turkey and chicken farms in North Carolina and Cluster 3 in turkey farms in Missouri, while showing that detected US aMPV-B field strains remained highly similar across our dataset and available GenBank comparisons. The data also document vaccine-derived genomes of aMPV subtypes A and B in diseased flocks after the introduction of imported live vaccines. These observations do not measure homologous or heterologous vaccine protection, but they highlight the need for continued whole-genome surveillance that can distinguish field strains from vaccine-derived lineages and track emerging G-gene diversity in US poultry.

## Data Availability

The whole-genome sequences generated in this study are deposited in NCBI GenBank. The aMPV-A genomes are available under accession numbers PZ290666-PZ290709, and the aMPV-B genomes are available under accession numbers PX994324-PX994329 and PZ290625-PZ290665. Analysis and figure-generation scripts are available at https://github.com/navduhan/ampv-targeted.

## Supplementary material

Supplementary Table 1 contains sample metadata, including Ct values, host species, age, clinical sample type, submission dates, vaccination status, geographic origin, assembly status, and GenBank accession numbers for recovered genomes, for all subtype-positive clinical samples analyzed in this study. Recovered study genomes are listed using the manuscript ADRDL identifiers, whereas non-recovered entries are retained as screening-only NR-A and NR-B records without recovered genome designations.

Supplementary Table 2 provides the subtype-specific tiled primer schemes, including primer names, pool assignments, genomic coordinates, and 5^′^-to-3^′^ sequences for the aMPV-A and aMPV-B panels.

Supplementary Table 3 provides primer- and amplicon-level coverage summaries documenting interval-level coverage patterns across the subtype A and subtype B tiled panels.

Supplementary Table 4 summarizes paper-defined subtype A vaccine-lineage marker positions and vaccine progenitor comparisons for the subtype A vaccine-derived genomes together with lineage-specific nucleotide and amino-acid differences relative to the subtype B vaccine references, with the VCO3/50 comparison anchored to the published VCO3/50 states reported by Sugiyama et al. (2010).

Supplementary Figure 1 shows the Ct-versus-recovery performance summary.

Supplementary Figure 2 shows the exploratory pooled recovery-model comparison.

Supplementary Figure 3 shows the non-G, non-WGS phylogenies for aMPV-A.

Supplementary Figure 4 shows the focused aMPV-A G-protein hotspot alignment for Cluster 2 and Cluster 3 relative to the Cluster 1 consensus.

Supplementary Figure 5 shows the non-G, non-WGS phylogenies for aMPV-B.

Supplementary Figure 6 shows the subtype A vaccine-marker state summary corresponding to **Supplementary Table 4**, and **Supplementary Figures 7**-**S9** show the additional FEL, MEME, and BUSTED selection-analysis summaries. Each supplementary file is named with its corresponding S-number, and the complete table descriptions and figure captions are provided in the accompanying Supplementary Captions document.
