# Supplementary material for: Genomic characterization of avian metapneumovirus subtypes A and B in United States poultry by targeted amplicon sequencing
Source: Front Cell Infect Microbiol. 2026 Aug 21;16:1882507. doi: 10.3389/fcimb.2026.1882507 (PMC13541459; doi:10.3389/fcimb.2026.1882507)
Supplement: Supplementary file 1 [file DataSheet1.pdf]

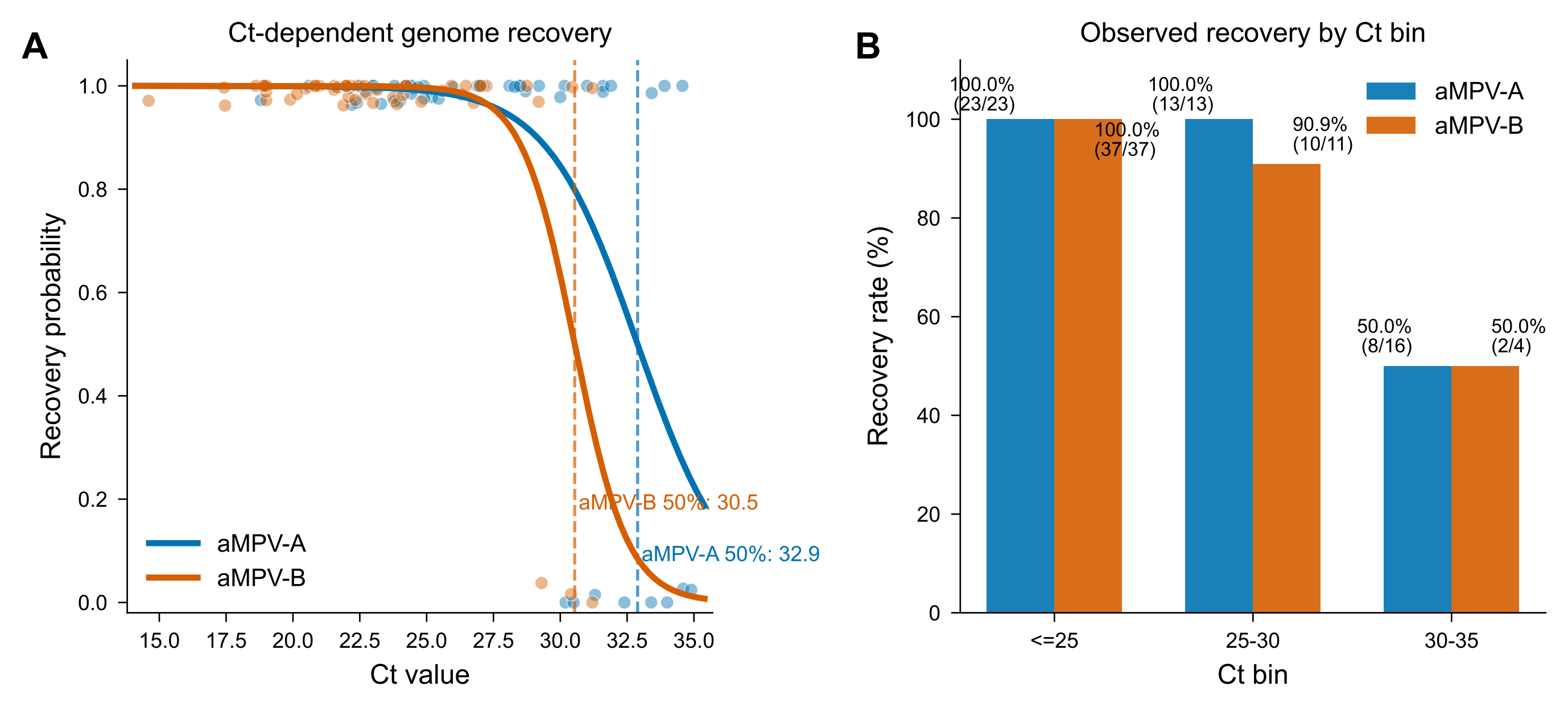
A
Ct-dependent genome recovery
Recovery probability
Ct value
aMPV-A
aMPV-B
aMPV-B 50%: 30.5
aMPV-A 50%: 32.9
1.0
0.8
0.6
0.4
0.2
0.0
15.0
17.5
20.0
22.5
25.0
27.5
30.0
32.5
35.0
B
Observed recovery by Ct bin
Recovery rate (%)
Ct bin
100.0% (23/23)
100.0% (37/37)
100.0% (13/13)
90.9% (10/11)
50.0% (8/16)
50.0% (2/4)
<=25
25-30
30-35
0
20
40
60
80
100
