# Supplementary material for: Genomic characterization of avian metapneumovirus subtypes A and B in United States poultry by targeted amplicon sequencing
Source: Front Cell Infect Microbiol. 2026 Aug 21;16:1882507. doi: 10.3389/fcimb.2026.1882507 (PMC13541459; doi:10.3389/fcimb.2026.1882507)
Supplement: Supplementary file 2 [file DataSheet10.pdf]

Supplementary Tables and Figure Captions

Genomic Characterization of Avian Metapneumovirus Subtypes A and B in United States Poultry by Targeted Amplicon Sequencing

Supplementary Tables

Supplementary Table S1. Sample metadata and sequencing metrics. De-identified sample-level metadata and sequencing summary metrics for all subtype-positive clinical samples analyzed in this study, including host species, sample type, state, submission date, vaccination history, Ct value, assembly status, and recovery metrics for successfully recovered genomes. Recovered study genomes are listed using manuscript ADRDL identifiers and mapped to their GenBank accession numbers; non-recovered entries are retained as screening-only NR-A and NR-B records. File: `Supplementary_Table_S1_Sample_Metadata.xlsx`.

Supplementary Table S2. Subtype-specific tiled primer schemes. Primer names, pool assignments, genomic coordinates, and 5′-to-3′ primer sequences used for targeted amplicon sequencing of aMPV-A and aMPV-B. File: `Supplementary_Table_S2_Primer_Schemes.xlsx`.

Supplementary Table S3. Primer- and amplicon-level coverage. Primer- and amplicon-level coverage summaries across recovered aMPV-A and aMPV-B genomes, reporting interval-level coverage patterns across the tiled panels. File: `Supplementary_Table_S3_Primer_Amplicon_Coverage.xlsx`.

Supplementary Table S4. Vaccine-marker and progenitor comparisons. The aMPV-A marker sheet summarizes paper-defined subtype A vaccine-lineage positions from Catelli et al. (2006), Lupini et al. (2011), and Selim et al. (2026), mapped to DQ666911, JF424833, and the six US vaccine-derived genomes. The aMPV-B sheets summarize lineage-specific nucleotide and amino-acid differences relative to the 1062 vaccine reference and to VCO3/50 states reconstructed from the VCO3/60616 parent sequence together with the states reported in Sugiyama et al. (2010), Table 3. File: `Supplementary_Table_S4_Vaccine_Marker_Summary.xlsx`.

Supplementary Figures

Supplementary Figure S1. Subtype-specific genome-recovery performance. Genome recovery across the full screening cohort in relation to Ct for aMPV-A and aMPV-B, including recovered and non-recovered samples, fitted subtype-specific logistic curves, and empirical recovery rates across Ct bins. File: `Supplementary_Figure_S1_Ct_Recovery_Performance.pdf`.

Supplementary Figure S2. Recovery-model comparison. Exploratory pooled receiver operating characteristic summaries comparing the Ct-only recovery model with expanded descriptive models that additionally include subtype, host, and sample-type terms. File: `Supplementary_Figure_S2_Recovery_Model_Comparison.pdf`.

Supplementary Figure S3. aMPV-A non-G, non-whole-genome phylogenies. Maximum-likelihood phylogenetic trees for aMPV-A coding regions other than the G gene and whole genome, showing study genomes relative to selected reference and vaccine-related sequences. File: `Supplementary_Figure_S3_aMPV-A_NonG_NonWGS_Phylogenies.pdf`.

Supplementary Figure S4. aMPV-A G-protein hotspot alignment. Focused amino-acid alignment of the G-protein ectodomain hotspot at residues 209–275, showing dominant and minor Cluster 2 patterns and the Cluster 3 Missouri pattern relative to the Cluster 1 consensus. File: `Supplementary_Figure_S4_aMPV-A_G_Hotspot_Alignment.pdf`.

Supplementary Figure S5. aMPV-B non-G, non-whole-genome phylogenies. Maximum-likelihood phylogenetic trees for aMPV-B coding regions other than the G gene and whole genome, showing study genomes relative to selected reference and vaccine-related sequences. File: `Supplementary_Figure_S5_aMPV-B_NonG_NonWGS_Phylogenies.pdf`.

Supplementary Figure S6. Subtype A vaccine-marker states. Genome-region map of paper-defined subtype A vaccine-lineage marker positions and corresponding nucleotide states in Vaccine 1, the Italy vaccine-derived sequence JF424833, the vaccine progenitor DQ666911, and six US subtype A vaccine-derived genomes. File: `Supplementary_Figure_S6_Vaccine_Marker_Genome_Map.pdf`.

Supplementary Figure S7. FEL G-gene selection analysis. Fixed Effects Likelihood site-wise selection results for the G gene of aMPV-A and aMPV-B, highlighting codons with evidence of non-neutral evolution under the applied significance threshold. File: `Supplementary_Figure_S7_FEL_G_Gene_Selection.pdf`.

Supplementary Figure S8. MEME G-gene selection analysis. Mixed Effects Model of Evolution site-wise results for the G gene of aMPV-A and aMPV-B, identifying codons with evidence of episodic diversifying selection. File: `Supplementary_Figure_S8_MEME_G_Gene_Selection.pdf`.

Supplementary Figure S9. BUSTED gene-wise selection analysis. Gene-wise BUSTED summaries across the principal coding regions of aMPV-A and aMPV-B, reporting gene-level tests for episodic diversifying selection. File: `Supplementary_Figure_S9_BUSTED_GeneWise_Selection.pdf`.
