# Supplementary material for: Genomic characterization of avian metapneumovirus subtypes A and B in United States poultry by targeted amplicon sequencing
Source: Front Cell Infect Microbiol. 2026 Aug 21;16:1882507. doi: 10.3389/fcimb.2026.1882507 (PMC13541459; doi:10.3389/fcimb.2026.1882507)
Supplement: Supplementary file 3 [file DataSheet2.pdf]

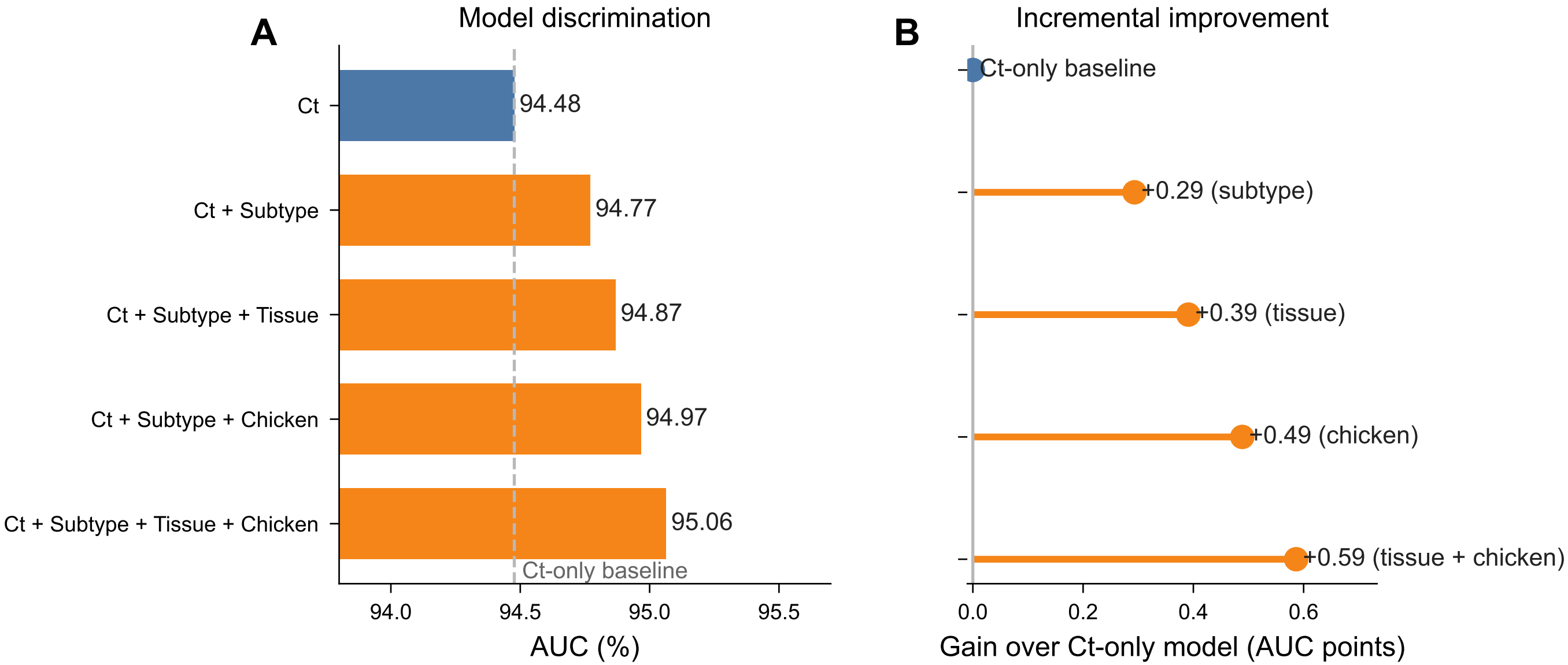
A
Model discrimination
Ct
Ct + Subtype
Ct + Subtype + Tissue
Ct + Subtype + Chicken
Ct + Subtype + Tissue + Chicken
94.48
94.77
94.87
94.97
95.06
Ct-only baseline
94.0
94.5
95.0
95.5
AUC (%)
B
Incremental improvement
Ct-only baseline
+0.29 (subtype)
+0.39 (tissue)
+0.49 (chicken)
+0.59 (tissue + chicken)
0.0
0.2
0.4
0.6
Gain over Ct-only model (AUC points)
