# Supplementary material for: Genomic characterization of avian metapneumovirus subtypes A and B in United States poultry by targeted amplicon sequencing
Source: Front Cell Infect Microbiol. 2026 Aug 21;16:1882507. doi: 10.3389/fcimb.2026.1882507 (PMC13541459; doi:10.3389/fcimb.2026.1882507)

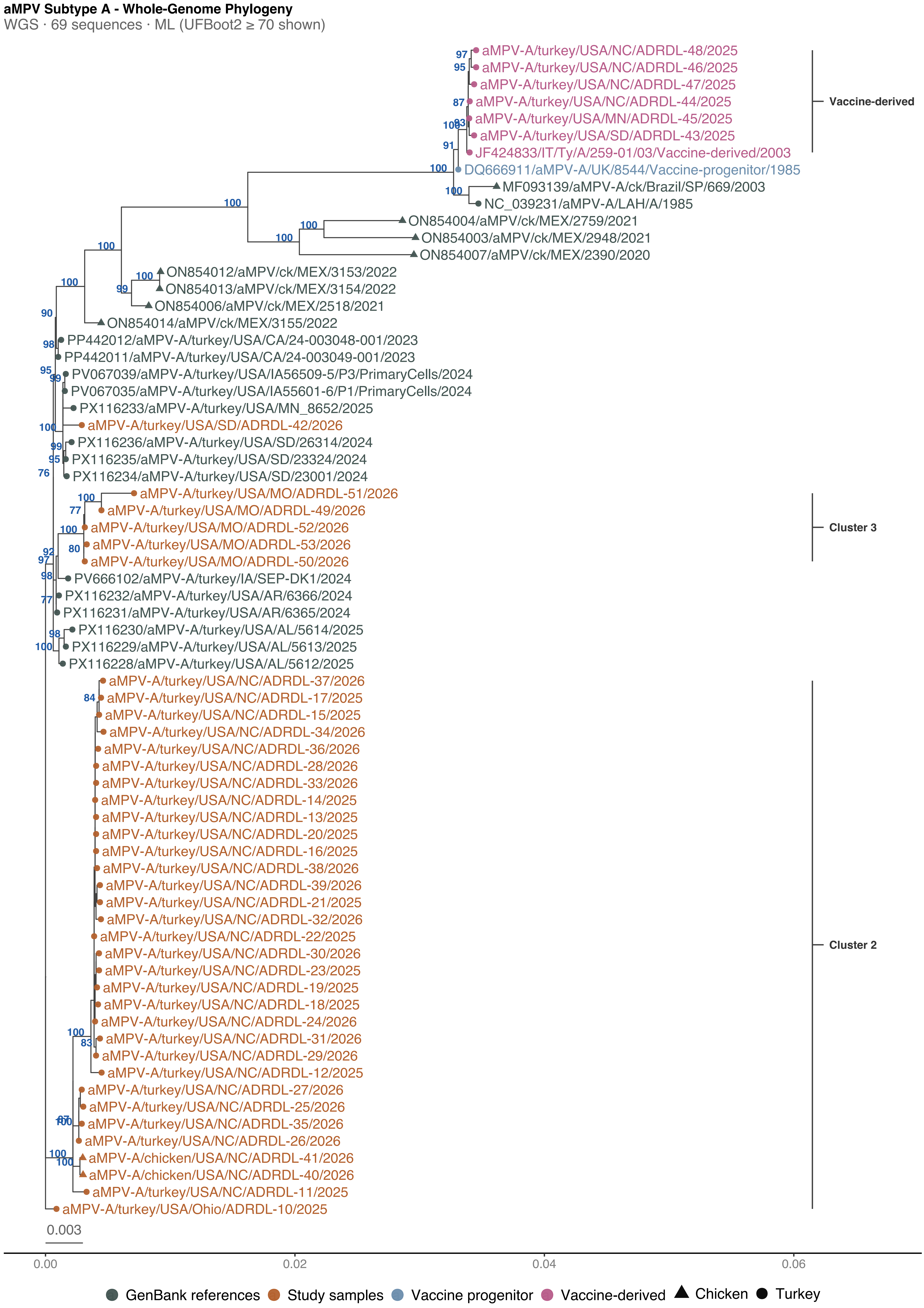

aMPV Subtype A - Whole-Genome Phylogeny
WGS · 69 sequences · ML (UFBoot2 ≥ 70 shown)
aMPV-A/turkey/USA/NC/ADRDL-48/2025
aMPV-A/turkey/USA/NC/ADRDL-46/2025
aMPV-A/turkey/USA/NC/ADRDL-47/2025
aMPV-A/turkey/USA/NC/ADRDL-44/2025
aMPV-A/turkey/USA/MN/ADRDL-45/2025
aMPV-A/turkey/USA/SD/ADRDL-43/2025
JF424833/IT/Ty/A/259-01/03/Vaccine-derived/2003
DQ666911/aMPV-A/UK/8544/Vaccine-progenitor/1985
MF093139/aMPV-A/ck/Brazil/SP/669/2003
NC_039231/aMPV-A/LAH/A/1985
ON854004/aMPV/ck/MEX/2759/2021
ON854003/aMPV/ck/MEX/2948/2021
ON854007/aMPV/ck/MEX/2390/2020
ON854012/aMPV/ck/MEX/3153/2022
ON854013/aMPV/ck/MEX/3154/2022
ON854006/aMPV/ck/MEX/2518/2021
ON854014/aMPV/ck/MEX/3155/2022
PP442012/aMPV-A/turkey/USA/CA/24-003048-001/2023
PP442011/aMPV-A/turkey/USA/CA/24-003049-001/2023
PV067039/aMPV-A/turkey/USA/IA56509-5/P3/PrimaryCells/2024
PV067035/aMPV-A/turkey/USA/IA55601-6/P1/PrimaryCells/2024
PX116233/aMPV-A/turkey/USA/MN_8652/2025
aMPV-A/turkey/USA/SD/ADRDL-42/2026
PX116236/aMPV-A/turkey/USA/SD/26314/2024
PX116235/aMPV-A/turkey/USA/SD/23324/2024
PX116234/aMPV-A/turkey/USA/SD/23001/2024
aMPV-A/turkey/USA/MO/ADRDL-51/2026
aMPV-A/turkey/USA/MO/ADRDL-49/2026
aMPV-A/turkey/USA/MO/ADRDL-52/2026
aMPV-A/turkey/USA/MO/ADRDL-53/2026
aMPV-A/turkey/USA/MO/ADRDL-50/2026
PV666102/aMPV-A/turkey/IA/SEP-DK1/2024
PX116232/aMPV-A/turkey/USA/AR/6366/2024
PX116231/aMPV-A/turkey/USA/AR/6365/2024
PX116230/aMPV-A/turkey/USA/AL/5614/2025
PX116229/aMPV-A/turkey/USA/AL/5613/2025
PX116228/aMPV-A/turkey/USA/AL/5612/2025
aMPV-A/turkey/USA/NC/ADRDL-37/2026
aMPV-A/turkey/USA/NC/ADRDL-17/2025
aMPV-A/turkey/USA/NC/ADRDL-15/2025
aMPV-A/turkey/USA/NC/ADRDL-34/2026
aMPV-A/turkey/USA/NC/ADRDL-36/2026
aMPV-A/turkey/USA/NC/ADRDL-28/2026
aMPV-A/turkey/USA/NC/ADRDL-33/2026
aMPV-A/turkey/USA/NC/ADRDL-14/2025
aMPV-A/turkey/USA/NC/ADRDL-13/2025
aMPV-A/turkey/USA/NC/ADRDL-20/2025
aMPV-A/turkey/USA/NC/ADRDL-16/2025
aMPV-A/turkey/USA/NC/ADRDL-38/2026
aMPV-A/turkey/USA/NC/ADRDL-39/2026
aMPV-A/turkey/USA/NC/ADRDL-21/2025
aMPV-A/turkey/USA/NC/ADRDL-32/2026
aMPV-A/turkey/USA/NC/ADRDL-22/2025
aMPV-A/turkey/USA/NC/ADRDL-30/2026
aMPV-A/turkey/USA/NC/ADRDL-23/2025
aMPV-A/turkey/USA/NC/ADRDL-19/2025
aMPV-A/turkey/USA/NC/ADRDL-18/2025
aMPV-A/turkey/USA/NC/ADRDL-24/2026
aMPV-A/turkey/USA/NC/ADRDL-31/2026
aMPV-A/turkey/USA/NC/ADRDL-29/2026
aMPV-A/turkey/USA/NC/ADRDL-12/2025
aMPV-A/turkey/USA/NC/ADRDL-27/2026
aMPV-A/turkey/USA/NC/ADRDL-25/2026
aMPV-A/turkey/USA/NC/ADRDL-35/2026
aMPV-A/turkey/USA/NC/ADRDL-26/2026
aMPV-A/chicken/USA/NC/ADRDL-41/2026
aMPV-A/chicken/USA/NC/ADRDL-40/2026
aMPV-A/turkey/USA/NC/ADRDL-11/2025
aMPV-A/turkey/USA/Ohio/ADRDL-10/2025
Vaccine-derived
Cluster 3
Cluster 2
0.003
0.00
0.02
0.04
0.06
GenBank references
Study samples
Vaccine progenitor
Vaccine-derived
Chicken
Turkey

aMPV Subtype A - G Gene Phylogeny

G gene · 69 sequences · ML (UFBoot2 ≥ 70 shown)

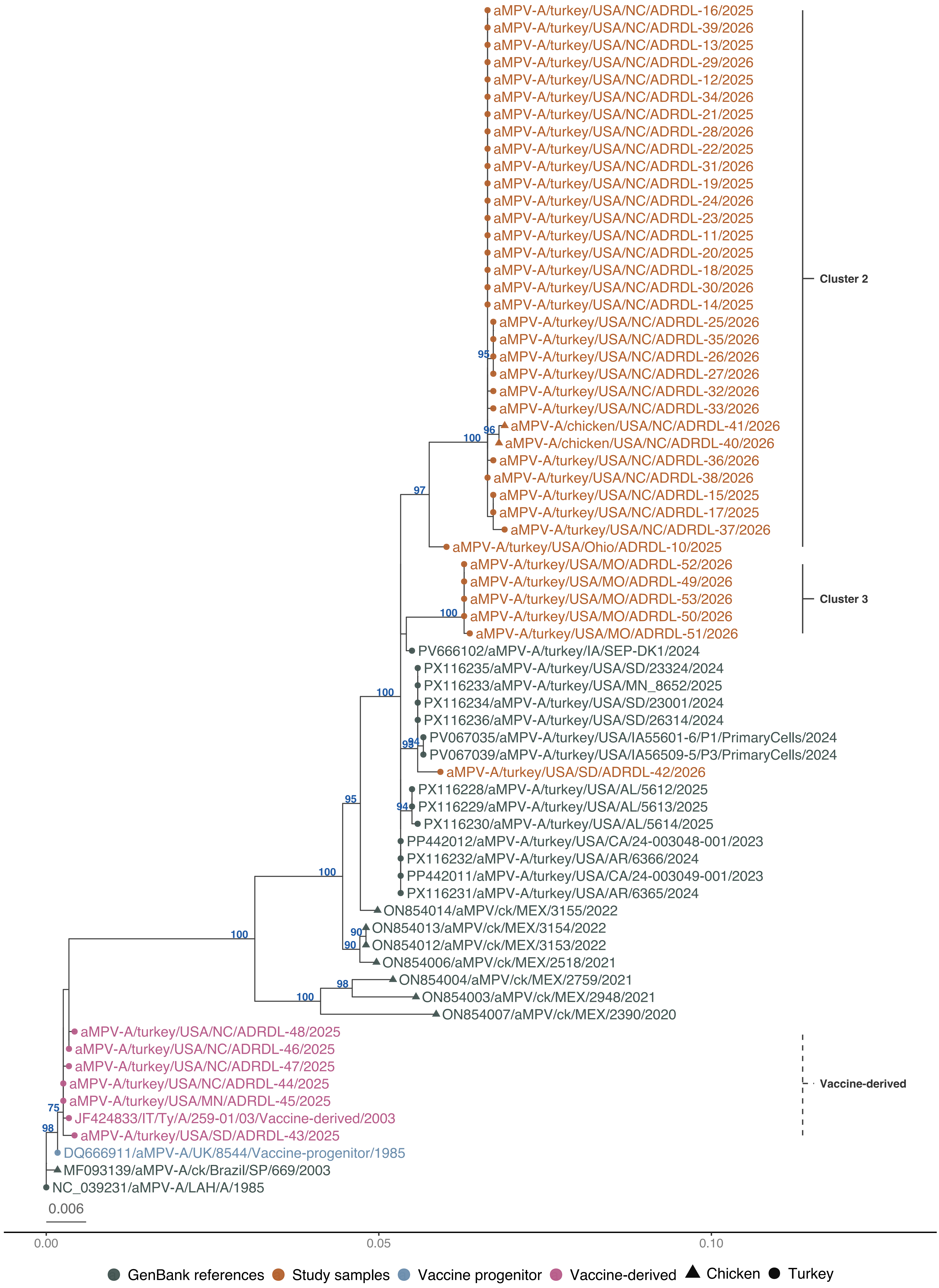

aMPV Subtype A - F Gene Phylogeny

F gene · 69 sequences · ML (UFBoot2 ≥ 70 shown)

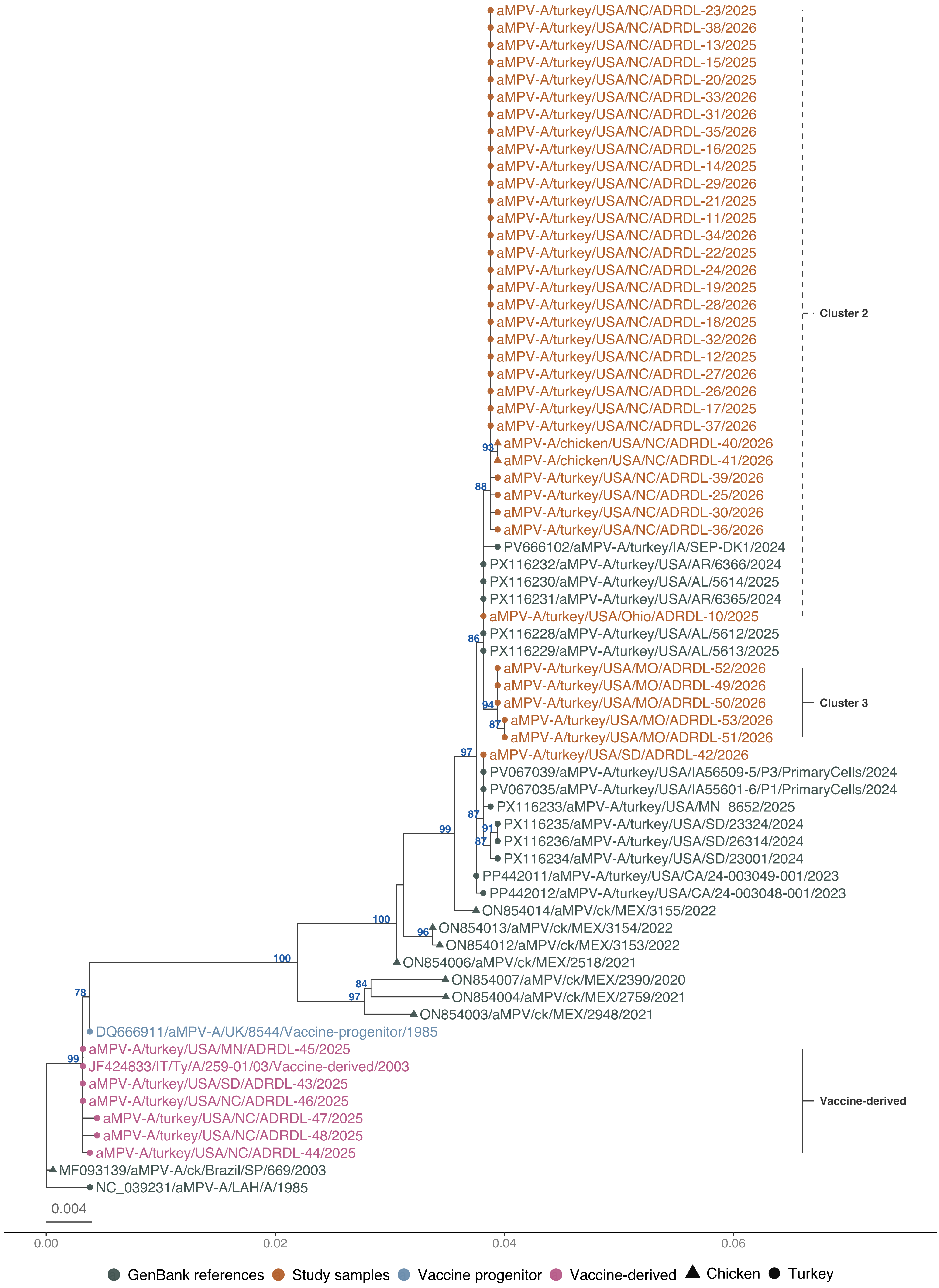

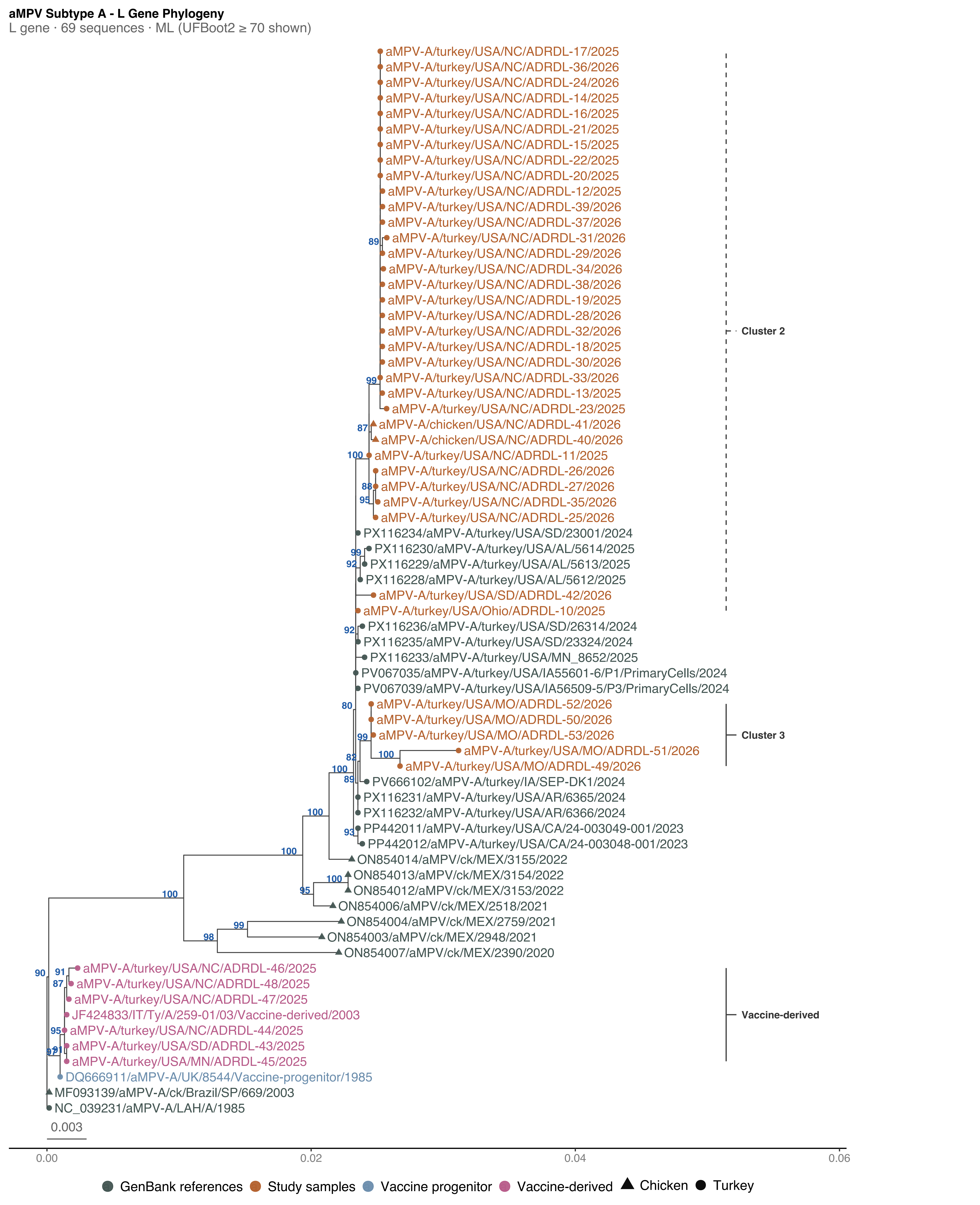
aMPV Subtype A - L Gene Phylogeny
L gene · 69 sequences · ML (UFBoot2 ≥ 70 shown)
aMPV-A/turkey/USA/NC/ADRDL-17/2025
aMPV-A/turkey/USA/NC/ADRDL-36/2026
aMPV-A/turkey/USA/NC/ADRDL-24/2026
aMPV-A/turkey/USA/NC/ADRDL-14/2025
aMPV-A/turkey/USA/NC/ADRDL-16/2025
aMPV-A/turkey/USA/NC/ADRDL-21/2025
aMPV-A/turkey/USA/NC/ADRDL-15/2025
aMPV-A/turkey/USA/NC/ADRDL-22/2025
aMPV-A/turkey/USA/NC/ADRDL-20/2025
aMPV-A/turkey/USA/NC/ADRDL-12/2025
aMPV-A/turkey/USA/NC/ADRDL-39/2026
aMPV-A/turkey/USA/NC/ADRDL-37/2026
aMPV-A/turkey/USA/NC/ADRDL-31/2026
aMPV-A/turkey/USA/NC/ADRDL-29/2026
aMPV-A/turkey/USA/NC/ADRDL-34/2026
aMPV-A/turkey/USA/NC/ADRDL-38/2026
aMPV-A/turkey/USA/NC/ADRDL-19/2025
aMPV-A/turkey/USA/NC/ADRDL-28/2026
aMPV-A/turkey/USA/NC/ADRDL-32/2026
aMPV-A/turkey/USA/NC/ADRDL-18/2025
aMPV-A/turkey/USA/NC/ADRDL-30/2026
aMPV-A/turkey/USA/NC/ADRDL-33/2026
aMPV-A/turkey/USA/NC/ADRDL-13/2025
aMPV-A/turkey/USA/NC/ADRDL-23/2025
aMPV-A/chicken/USA/NC/ADRDL-41/2026
aMPV-A/chicken/USA/NC/ADRDL-40/2026
aMPV-A/turkey/USA/NC/ADRDL-11/2025
aMPV-A/turkey/USA/NC/ADRDL-26/2026
aMPV-A/turkey/USA/NC/ADRDL-27/2026
aMPV-A/turkey/USA/NC/ADRDL-35/2026
aMPV-A/turkey/USA/NC/ADRDL-25/2026
PX116234/aMPV-A/turkey/USA/SD/23001/2024
PX116230/aMPV-A/turkey/USA/AL/5614/2025
PX116229/aMPV-A/turkey/USA/AL/5613/2025
PX116228/aMPV-A/turkey/USA/AL/5612/2025
aMPV-A/turkey/USA/SD/ADRDL-42/2026
aMPV-A/turkey/USA/Ohio/ADRDL-10/2025
PX116236/aMPV-A/turkey/USA/SD/26314/2024
PX116235/aMPV-A/turkey/USA/SD/23324/2024
PX116233/aMPV-A/turkey/USA/MN_8652/2025
PV067035/aMPV-A/turkey/USA/IA55601-6/P1/PrimaryCells/2024
PV067039/aMPV-A/turkey/USA/IA56509-5/P3/PrimaryCells/2024
aMPV-A/turkey/USA/MO/ADRDL-52/2026
aMPV-A/turkey/USA/MO/ADRDL-50/2026
aMPV-A/turkey/USA/MO/ADRDL-53/2026
aMPV-A/turkey/USA/MO/ADRDL-51/2026
aMPV-A/turkey/USA/MO/ADRDL-49/2026
PV666102/aMPV-A/turkey/IA/SEP-DK1/2024
PX116231/aMPV-A/turkey/USA/AR/6365/2024
PX116232/aMPV-A/turkey/USA/AR/6366/2024
PP442011/aMPV-A/turkey/USA/CA/24-003049-001/2023
PP442012/aMPV-A/turkey/USA/CA/24-003048-001/2023
ON854014/aMPV/ck/MEX/3155/2022
ON854013/aMPV/ck/MEX/3154/2022
ON854012/aMPV/ck/MEX/3153/2022
ON854006/aMPV/ck/MEX/2518/2021
ON854004/aMPV/ck/MEX/2759/2021
ON854003/aMPV/ck/MEX/2948/2021
ON854007/aMPV/ck/MEX/2390/2020
aMPV-A/turkey/USA/NC/ADRDL-46/2025
aMPV-A/turkey/USA/NC/ADRDL-48/2025
aMPV-A/turkey/USA/NC/ADRDL-47/2025
JF424833/IT/Ty/A/259-01/03/Vaccine-derived/2003
aMPV-A/turkey/USA/NC/ADRDL-44/2025
aMPV-A/turkey/USA/SD/ADRDL-43/2025
aMPV-A/turkey/USA/MN/ADRDL-45/2025
DQ666911/aMPV-A/UK/8544/Vaccine-progenitor/1985
MF093139/aMPV-A/ck/Brazil/SP/669/2003
NC_039231/aMPV-A/LAH/A/1985
Cluster 2
Cluster 3
Vaccine-derived
0.003
0.00
0.02
0.04
0.06
GenBank references
Study samples
Vaccine progenitor
Vaccine-derived
Chicken
Turkey

aMPV Subtype A - M Gene Phylogeny

M gene · 69 sequences · ML (UFBoot2 ≥ 70 shown)

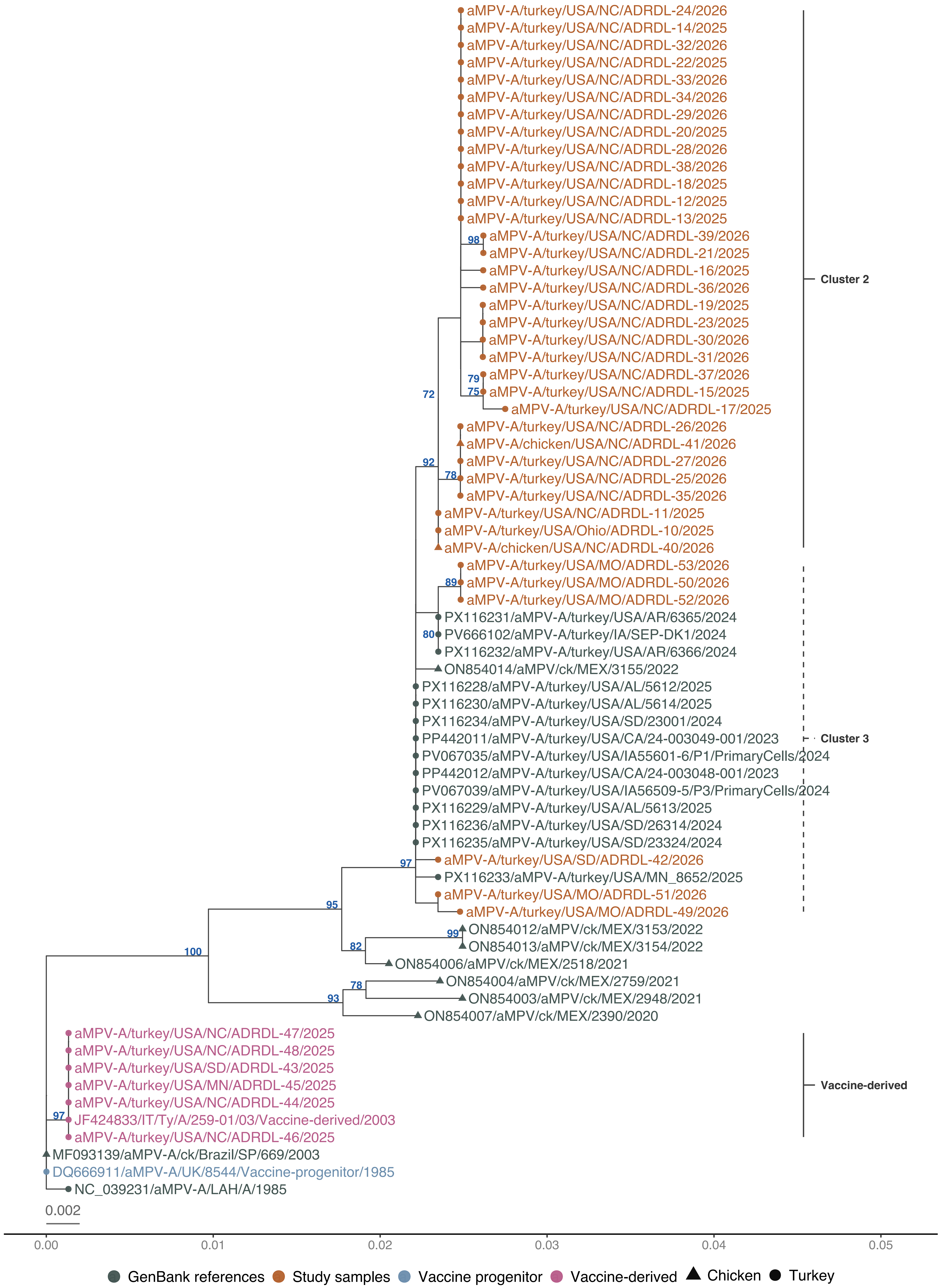

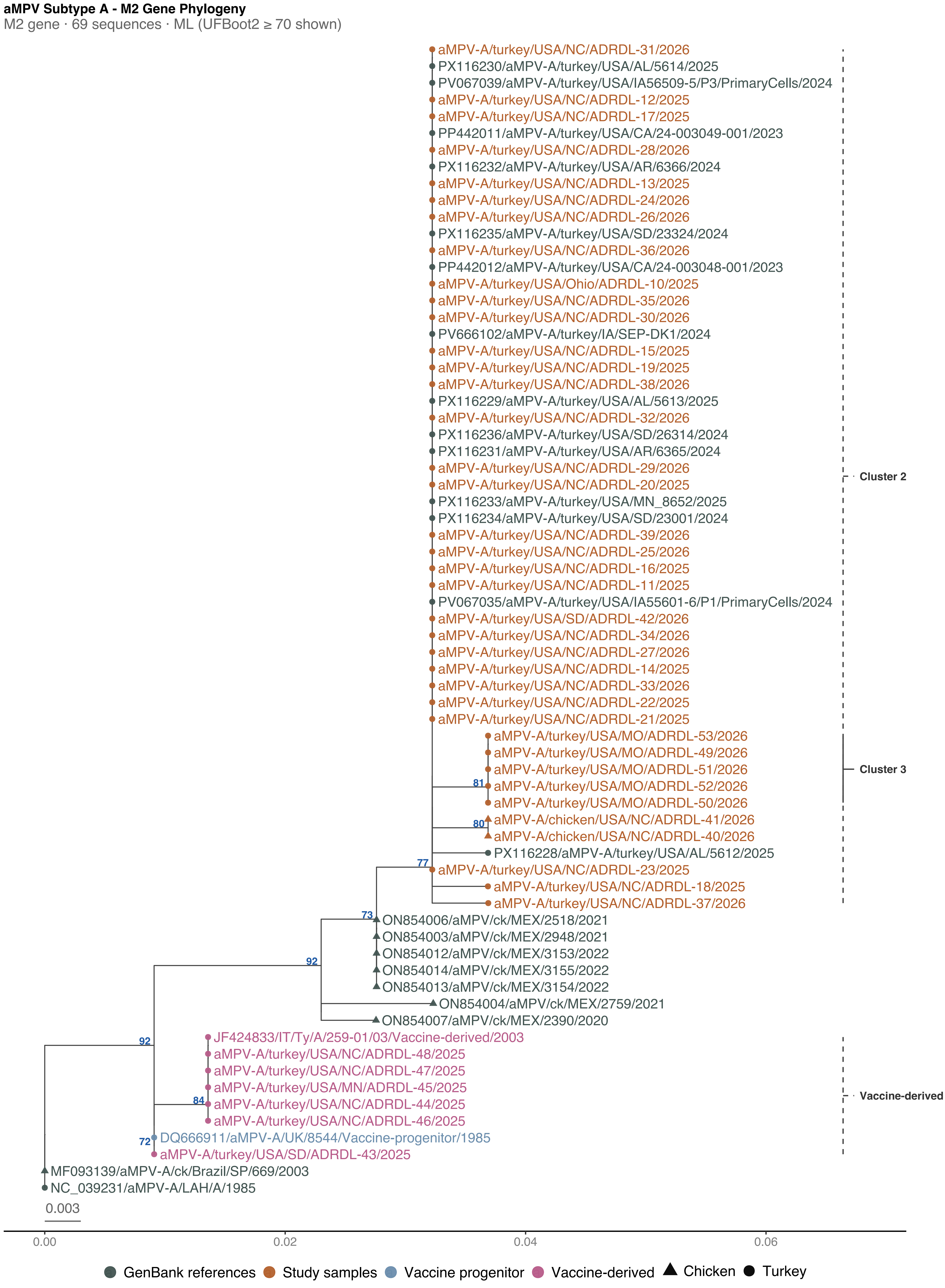
aMPV Subtype A - M2 Gene Phylogeny
M2 gene · 69 sequences · ML (UFBoot2 ≥ 70 shown)
aMPV-A/turkey/USA/NC/ADRDL-31/2026
PX116230/aMPV-A/turkey/USA/AL/5614/2025
PV067039/aMPV-A/turkey/USA/IA56509-5/P3/PrimaryCells/2024
aMPV-A/turkey/USA/NC/ADRDL-12/2025
aMPV-A/turkey/USA/NC/ADRDL-17/2025
PP442011/aMPV-A/turkey/USA/CA/24-003049-001/2023
aMPV-A/turkey/USA/NC/ADRDL-28/2026
PX116232/aMPV-A/turkey/USA/AR/6366/2024
aMPV-A/turkey/USA/NC/ADRDL-13/2025
aMPV-A/turkey/USA/NC/ADRDL-24/2026
aMPV-A/turkey/USA/NC/ADRDL-26/2026
PX116235/aMPV-A/turkey/USA/SD/23324/2024
aMPV-A/turkey/USA/NC/ADRDL-36/2026
PP442012/aMPV-A/turkey/USA/CA/24-003048-001/2023
aMPV-A/turkey/USA/Ohio/ADRDL-10/2025
aMPV-A/turkey/USA/NC/ADRDL-35/2026
aMPV-A/turkey/USA/NC/ADRDL-30/2026
PV666102/aMPV-A/turkey/IA/SEP-DK1/2024
aMPV-A/turkey/USA/NC/ADRDL-15/2025
aMPV-A/turkey/USA/NC/ADRDL-19/2025
aMPV-A/turkey/USA/NC/ADRDL-38/2026
PX116229/aMPV-A/turkey/USA/AL/5613/2025
aMPV-A/turkey/USA/NC/ADRDL-32/2026
PX116236/aMPV-A/turkey/USA/SD/26314/2024
PX116231/aMPV-A/turkey/USA/AR/6365/2024
aMPV-A/turkey/USA/NC/ADRDL-29/2026
aMPV-A/turkey/USA/NC/ADRDL-20/2025
PX116233/aMPV-A/turkey/USA/MN_8652/2025
PX116234/aMPV-A/turkey/USA/SD/23001/2024
aMPV-A/turkey/USA/NC/ADRDL-39/2026
aMPV-A/turkey/USA/NC/ADRDL-25/2026
aMPV-A/turkey/USA/NC/ADRDL-16/2025
aMPV-A/turkey/USA/NC/ADRDL-11/2025
PV067035/aMPV-A/turkey/USA/IA55601-6/P1/PrimaryCells/2024
aMPV-A/turkey/USA/SD/ADRDL-42/2026
aMPV-A/turkey/USA/NC/ADRDL-34/2026
aMPV-A/turkey/USA/NC/ADRDL-27/2026
aMPV-A/turkey/USA/NC/ADRDL-14/2025
aMPV-A/turkey/USA/NC/ADRDL-33/2026
aMPV-A/turkey/USA/NC/ADRDL-22/2025
aMPV-A/turkey/USA/NC/ADRDL-21/2025
aMPV-A/turkey/USA/MO/ADRDL-53/2026
aMPV-A/turkey/USA/MO/ADRDL-49/2026
aMPV-A/turkey/USA/MO/ADRDL-51/2026
81
aMPV-A/turkey/USA/MO/ADRDL-52/2026
aMPV-A/turkey/USA/MO/ADRDL-50/2026
80
aMPV-A/chicken/USA/NC/ADRDL-41/2026
aMPV-A/chicken/USA/NC/ADRDL-40/2026
PX116228/aMPV-A/turkey/USA/AL/5612/2025
77
aMPV-A/turkey/USA/NC/ADRDL-23/2025
aMPV-A/turkey/USA/NC/ADRDL-18/2025
aMPV-A/turkey/USA/NC/ADRDL-37/2026
73
ON854006/aMPV/ck/MEX/2518/2021
ON854003/aMPV/ck/MEX/2948/2021
92
ON854012/aMPV/ck/MEX/3153/2022
ON854014/aMPV/ck/MEX/3155/2022
ON854013/aMPV/ck/MEX/3154/2022
ON854004/aMPV/ck/MEX/2759/2021
ON854007/aMPV/ck/MEX/2390/2020
92
JF424833/IT/Ty/A/259-01/03/Vaccine-derived/2003
aMPV-A/turkey/USA/NC/ADRDL-48/2025
aMPV-A/turkey/USA/NC/ADRDL-47/2025
aMPV-A/turkey/USA/MN/ADRDL-45/2025
84
aMPV-A/turkey/USA/NC/ADRDL-44/2025
aMPV-A/turkey/USA/NC/ADRDL-46/2025
72
DQ666911/aMPV-A/UK/8544/Vaccine-progenitor/1985
aMPV-A/turkey/USA/SD/ADRDL-43/2025
MF093139/aMPV-A/ck/Brazil/SP/669/2003
NC_039231/aMPV-A/LAH/A/1985
Cluster 2
Cluster 3
Vaccine-derived
0.003
0.00
0.02
0.04
0.06
GenBank references
Study samples
Vaccine progenitor
Vaccine-derived
Chicken
Turkey

aMPV Subtype A - N Gene Phylogeny

N gene · 69 sequences · ML (UFBoot2 ≥ 70 shown)

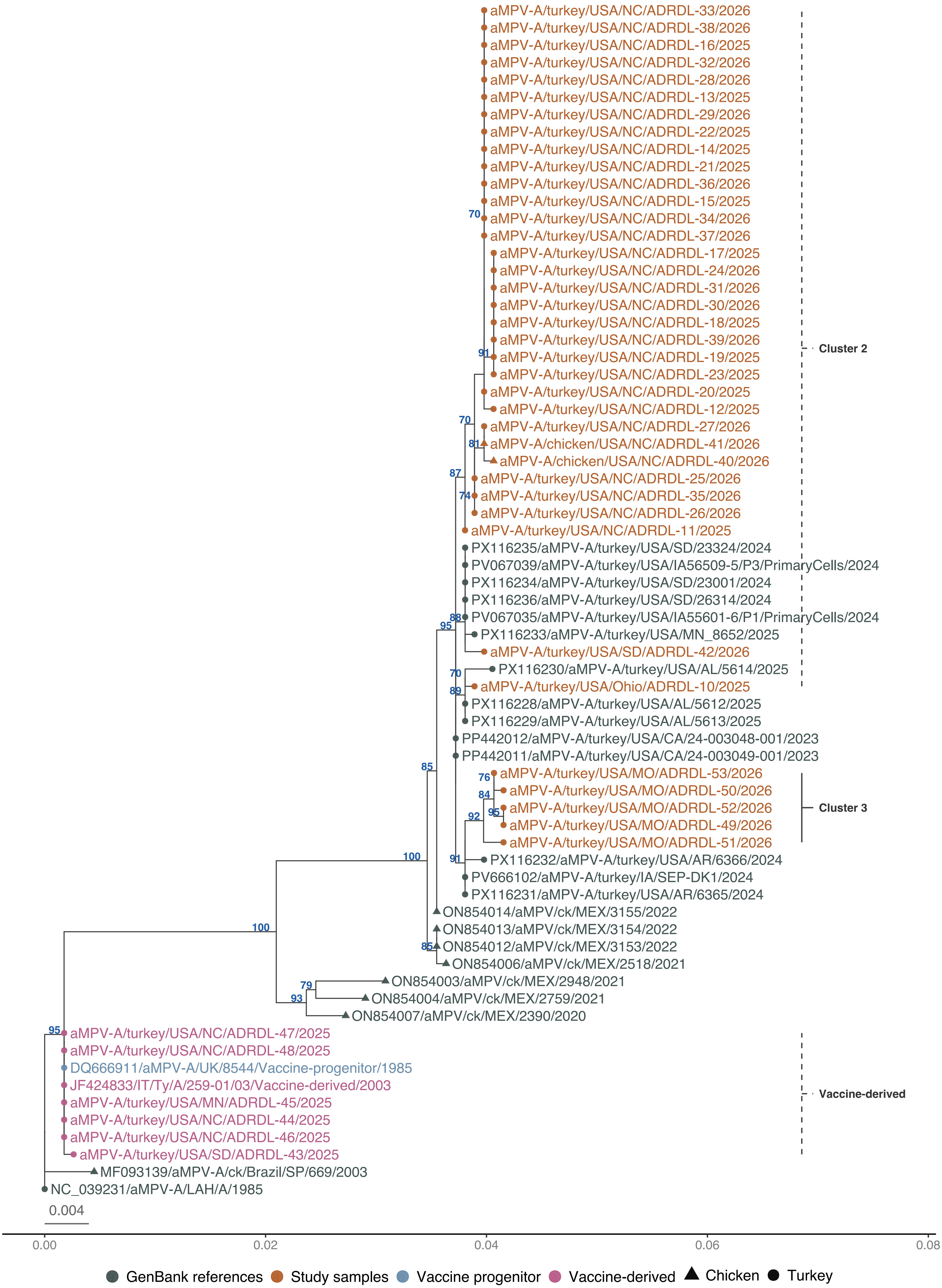

aMPV Subtype A - P Gene Phylogeny

P gene · 69 sequences · ML (UFBoot2 ≥ 70 shown)

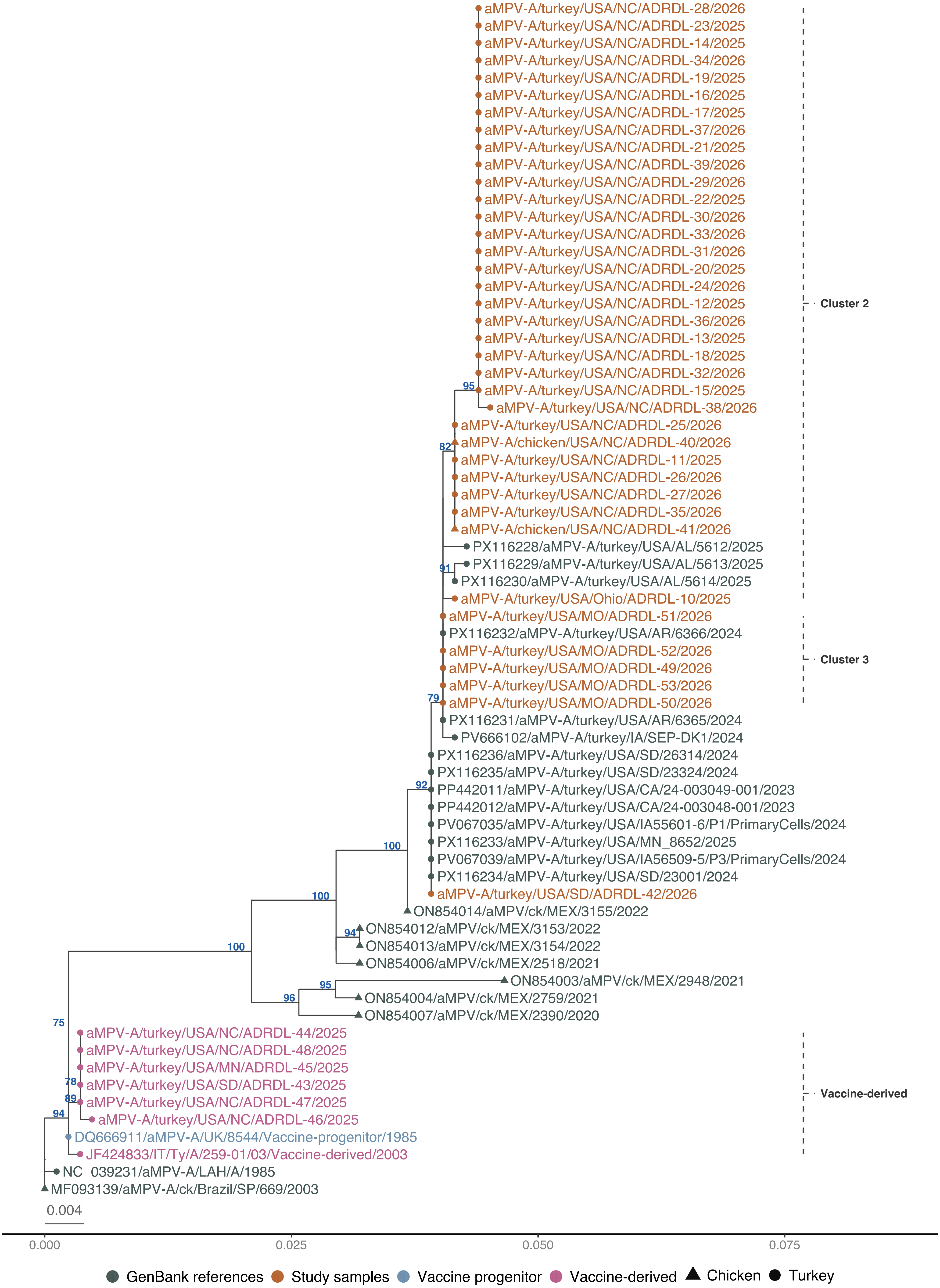

aMPV Subtype A - SH Gene Phylogeny

SH gene · 69 sequences · ML (UFBoot2 ≥ 70 shown)

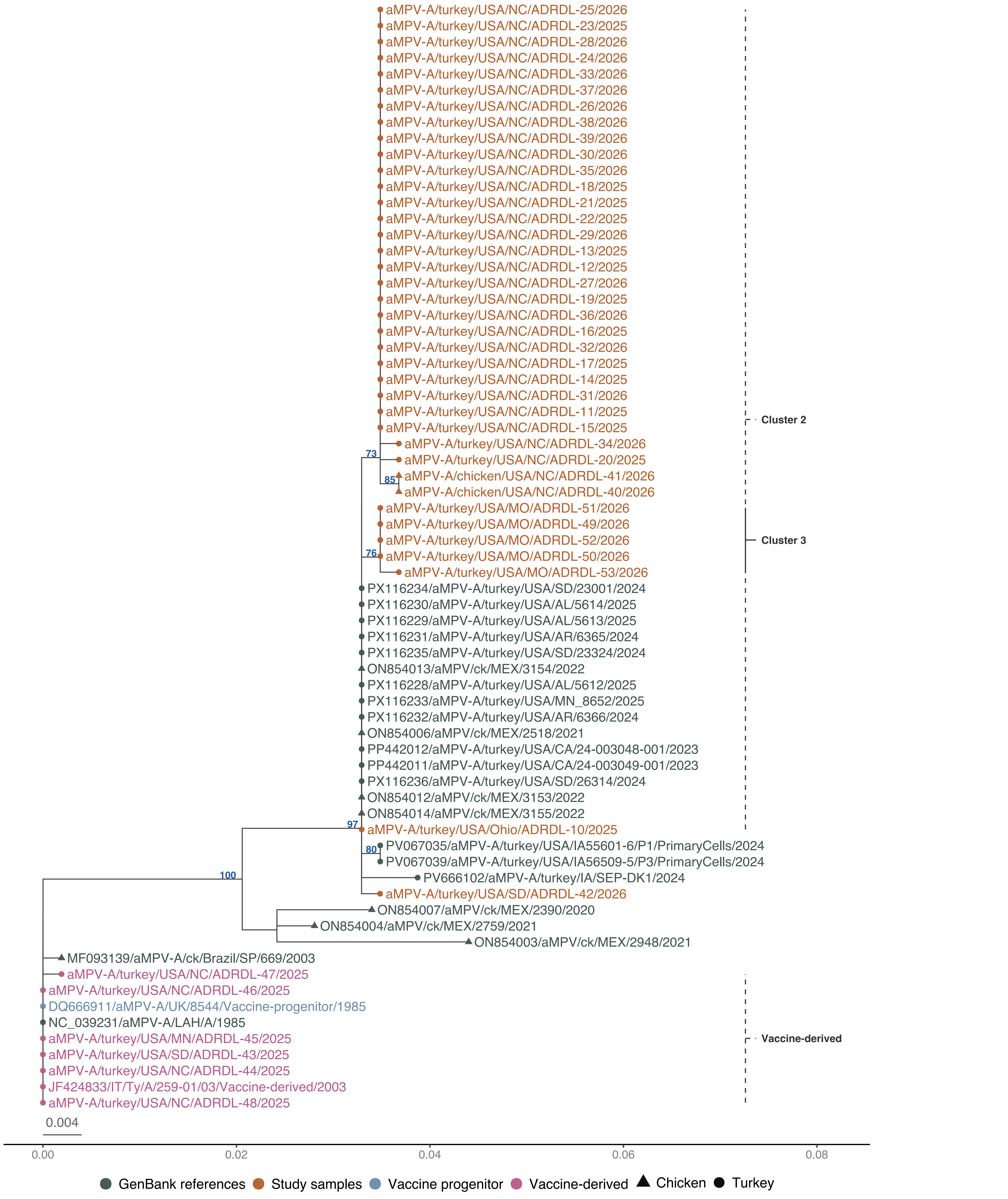

Supplement: Supplementary file 4 [file DataSheet3.pdf]
