# Supplementary material for: Genomic characterization of avian metapneumovirus subtypes A and B in United States poultry by targeted amplicon sequencing
Source: Front Cell Infect Microbiol. 2026 Aug 21;16:1882507. doi: 10.3389/fcimb.2026.1882507 (PMC13541459; doi:10.3389/fcimb.2026.1882507)
Supplement: Supplementary file 5 [file DataSheet4.pdf]

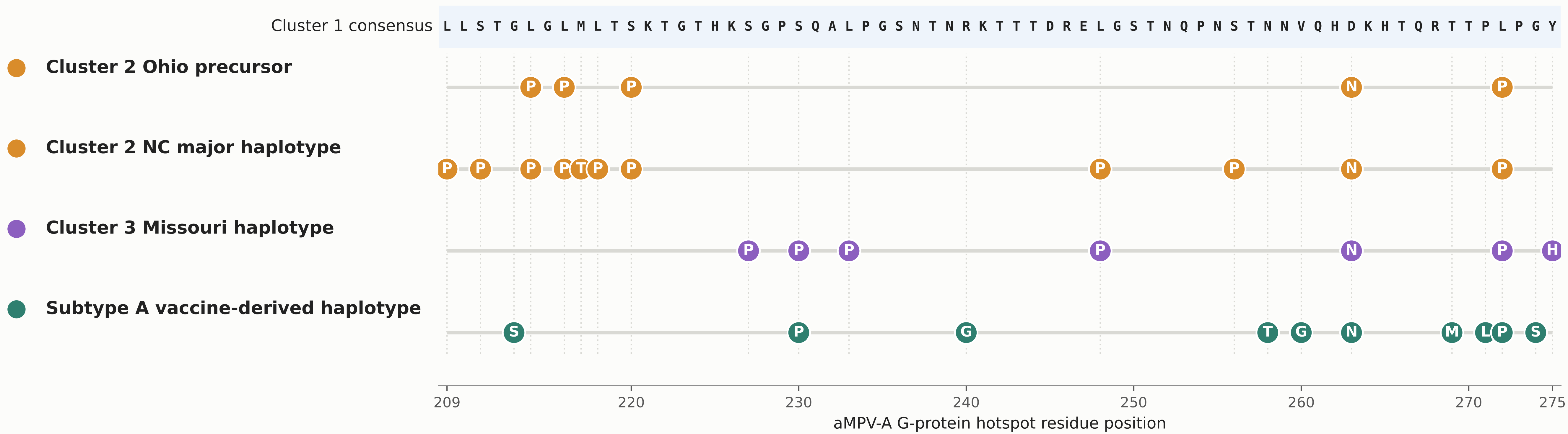

Cluster 1 consensus
L L S T G L G L M L T S K T G T H K S G P S Q A L P G S N T N R K T T T D R E L G S T N Q P N S T N N V Q H D K H T Q R T T P L P G Y
Cluster 2 Ohio precursor
Cluster 2 NC major haplotype
Cluster 3 Missouri haplotype
Subtype A vaccine-derived haplotype
209
220
230
240
250
260
270
275
aMPV-A G-protein hotspot residue position
