# Supplementary material for: Genomic characterization of avian metapneumovirus subtypes A and B in United States poultry by targeted amplicon sequencing
Source: Front Cell Infect Microbiol. 2026 Aug 21;16:1882507. doi: 10.3389/fcimb.2026.1882507 (PMC13541459; doi:10.3389/fcimb.2026.1882507)

aMPV Subtype B - Whole-Genome Phylogeny
WGS · 107 sequences · ML (UFBoot2 ≥ 70 shown)

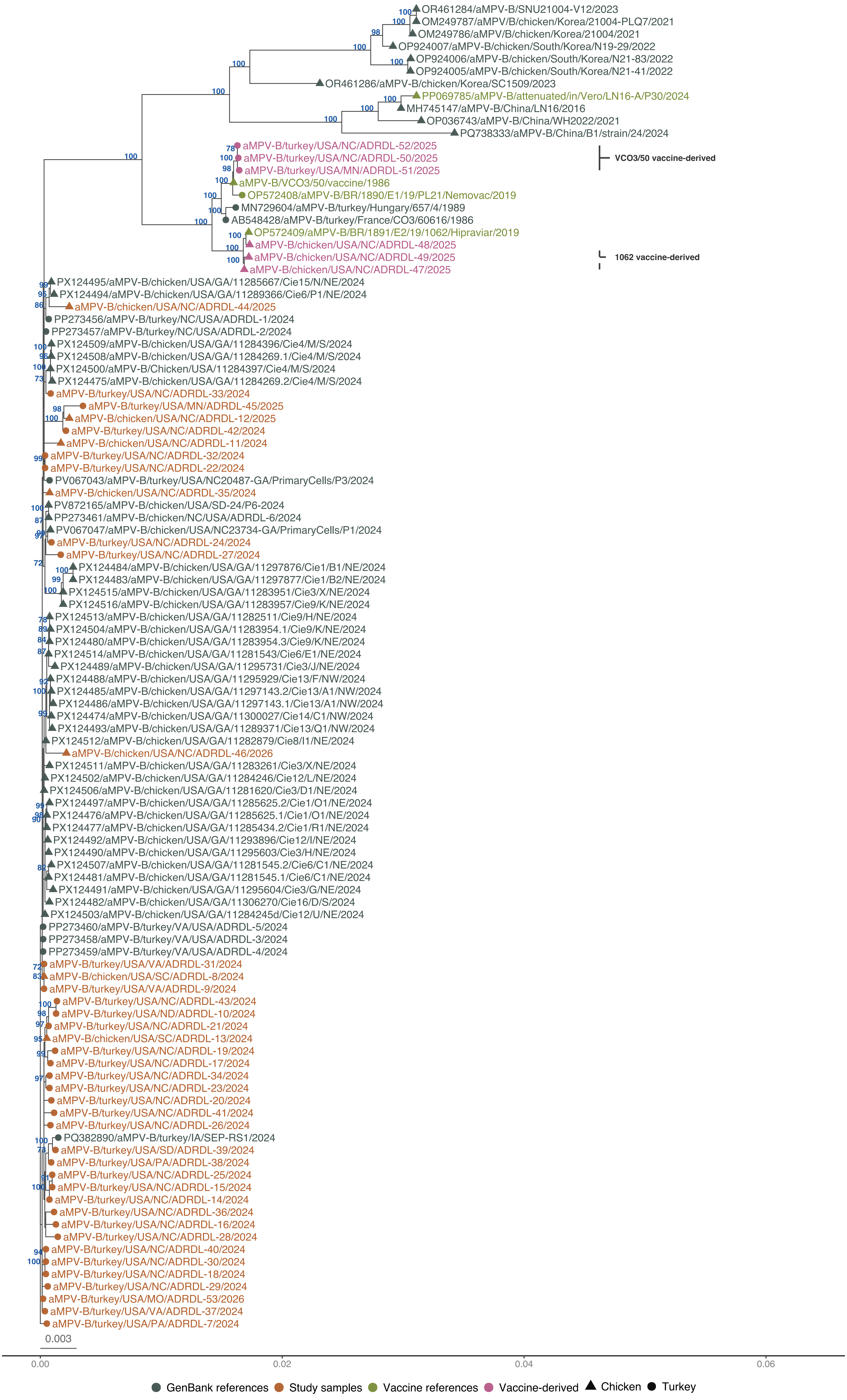

aMPV Subtype B - G Gene Phylogeny

G gene · 107 sequences · ML (UFBoot2 ≥ 70 shown)

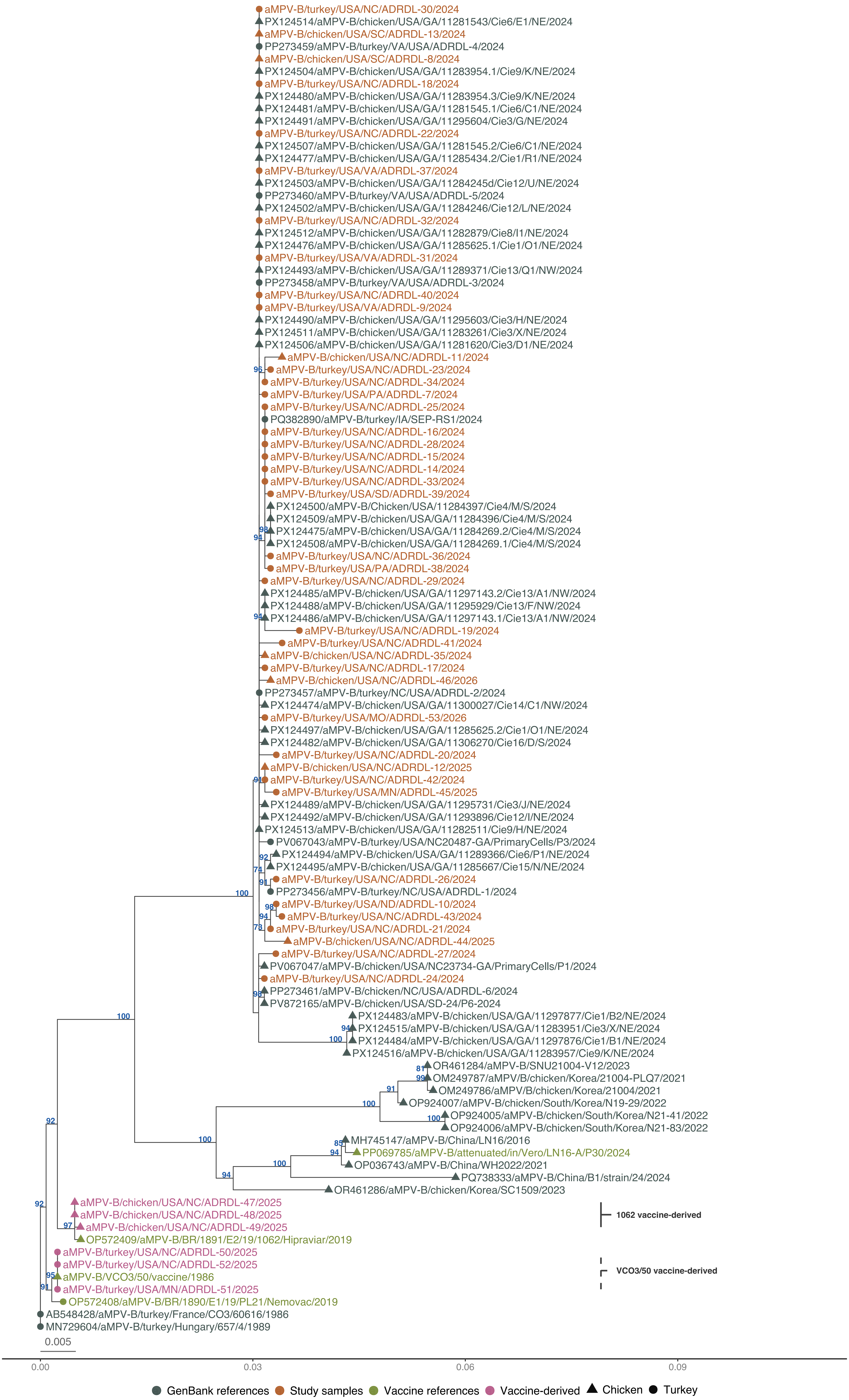

aMPV Subtype B - F Gene Phylogeny

F gene · 107 sequences · ML (UFBoot2 ≥ 70 shown)

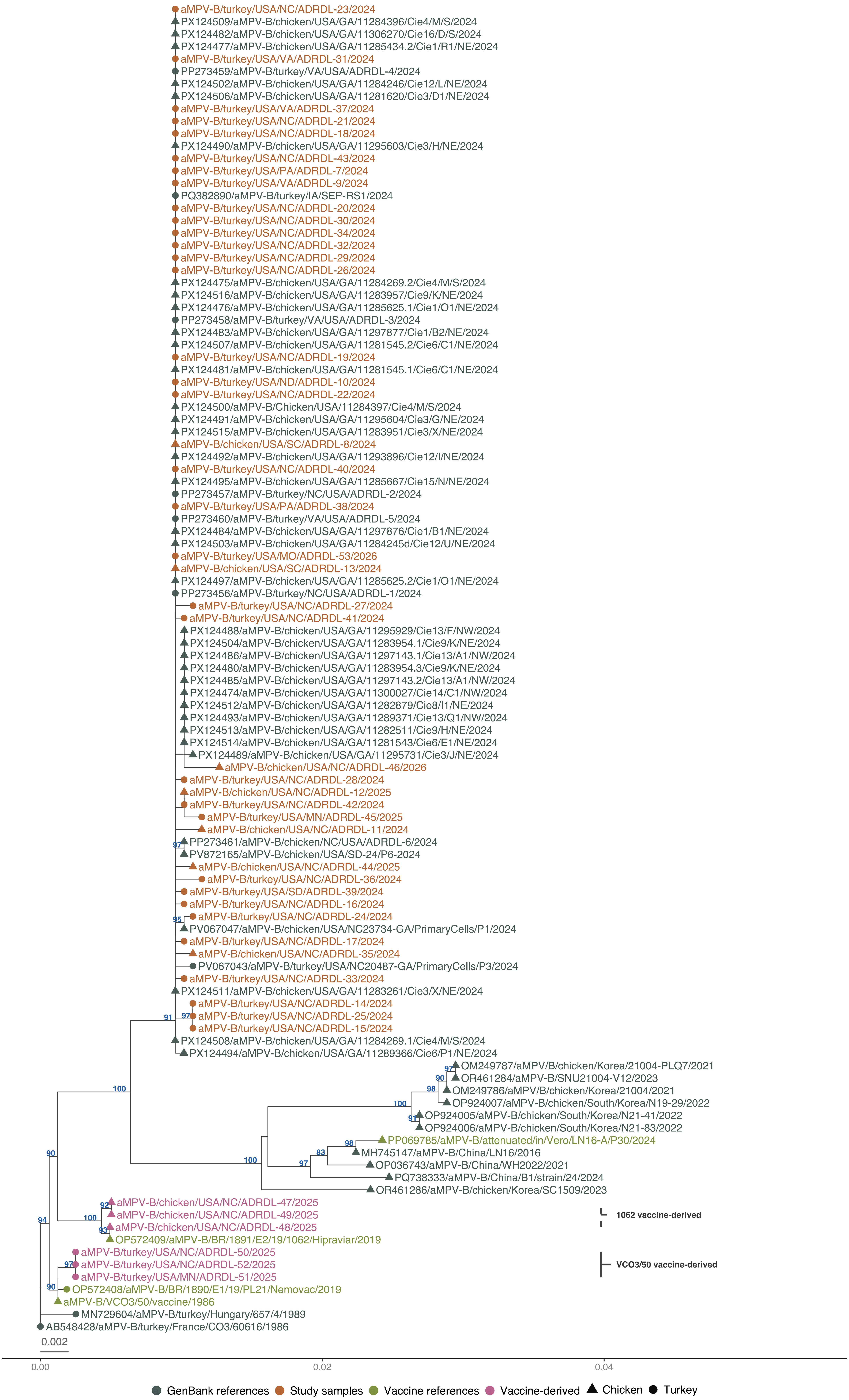

aMPV Subtype B - L Gene Phylogeny

L gene · 107 sequences · ML (UFBoot2 ≥ 70 shown)

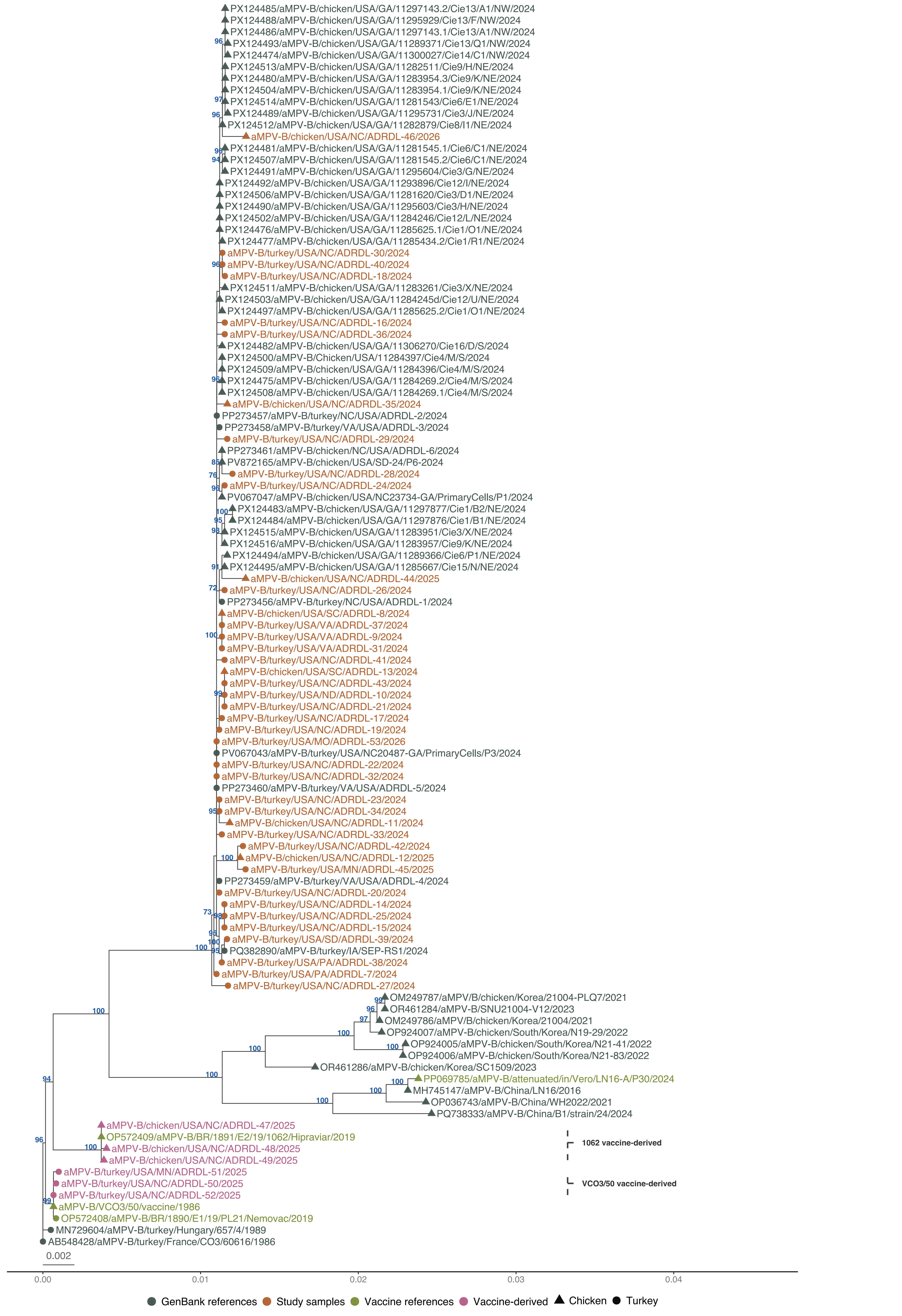

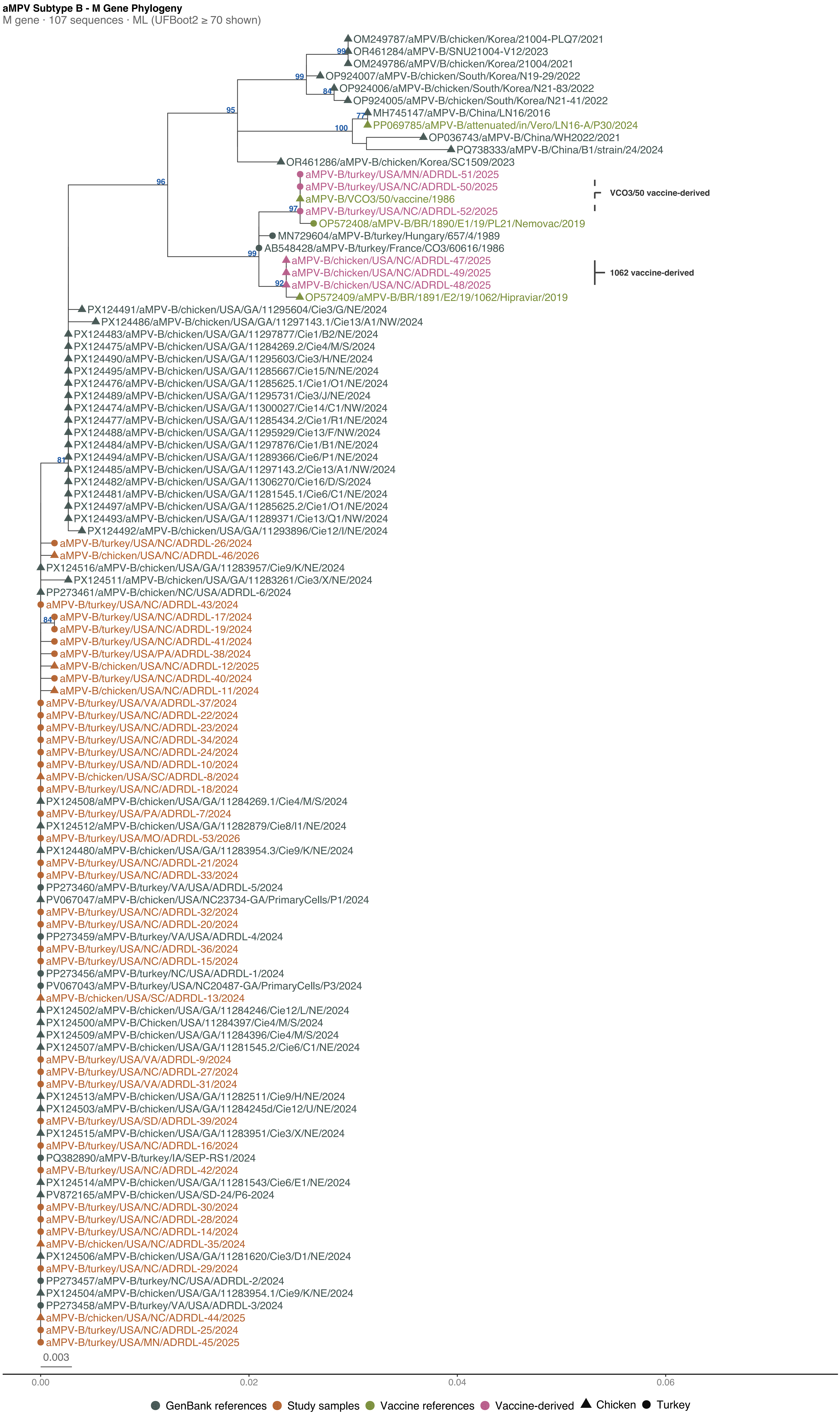

aMPV Subtype B - M Gene Phylogeny
M gene · 107 sequences · ML (UFBoot2 ≥ 70 shown)
OM249787/aMPV/B/chicken/Korea/21004-PLQ7/2021
99
OR461284/aMPV-B/SNU21004-V12/2023
OM249786/aMPV/B/chicken/Korea/21004/2021
99
OP924007/aMPV-B/chicken/South/Korea/N19-29/2022
84
OP924006/aMPV-B/chicken/South/Korea/N21-83/2022
OP924005/aMPV-B/chicken/South/Korea/N21-41/2022
95
77
MH745147/aMPV-B/China/LN16/2016
100
PP069785/aMPV-B/attenuated/in/Vero/LN16-A/P30/2024
OP036743/aMPV-B/China/WH2022/2021
PQ738333/aMPV-B/China/B1/strain/24/2024
OR461286/aMPV-B/chicken/Korea/SC1509/2023
96
aMPV-B/turkey/USA/MN/ADRDL-51/2025
aMPV-B/turkey/USA/NC/ADRDL-50/2025
VCO3/50 vaccine-derived
aMPV-B/VCO3/50/vaccine/1986
97
aMPV-B/turkey/USA/NC/ADRDL-52/2025
OP572408/aMPV-B/BR/1890/E1/19/PL21/Nemovac/2019
MN729604/aMPV-B/turkey/Hungary/657/4/1989
AB548428/aMPV-B/turkey/France/CO3/60616/1986
99
aMPV-B/chicken/USA/NC/ADRDL-47/2025
aMPV-B/chicken/USA/NC/ADRDL-49/2025
1062 vaccine-derived
92
aMPV-B/chicken/USA/NC/ADRDL-48/2025
OP572409/aMPV-B/BR/1891/E2/19/1062/Hipraviar/2019
PX124491/aMPV-B/chicken/USA/GA/11295604/Cie3/G/NE/2024
PX124486/aMPV-B/chicken/USA/GA/11297143.1/Cie13/A1/NW/2024
PX124483/aMPV-B/chicken/USA/GA/11297877/Cie1/B2/NE/2024
PX124475/aMPV-B/chicken/USA/GA/11284269.2/Cie4/M/S/2024
PX124490/aMPV-B/chicken/USA/GA/11295603/Cie3/H/NE/2024
PX124495/aMPV-B/chicken/USA/GA/11285667/Cie15/N/NE/2024
PX124476/aMPV-B/chicken/USA/GA/11285625.1/Cie1/O1/NE/2024
PX124489/aMPV-B/chicken/USA/GA/11295731/Cie3/J/NE/2024
PX124474/aMPV-B/chicken/USA/GA/11300027/Cie14/C1/NW/2024
PX124477/aMPV-B/chicken/USA/GA/11285434.2/Cie1/R1/NE/2024
PX124488/aMPV-B/chicken/USA/GA/11295929/Cie13/F/NW/2024
PX124484/aMPV-B/chicken/USA/GA/11297876/Cie1/B1/NE/2024
81
PX124494/aMPV-B/chicken/USA/GA/11289366/Cie6/P1/NE/2024
PX124485/aMPV-B/chicken/USA/GA/11297143.2/Cie13/A1/NW/2024
PX124482/aMPV-B/chicken/USA/GA/11306270/Cie16/D/S/2024
PX124481/aMPV-B/chicken/USA/GA/11281545.1/Cie6/C1/NE/2024
PX124497/aMPV-B/chicken/USA/GA/11285625.2/Cie1/O1/NE/2024
PX124493/aMPV-B/chicken/USA/GA/11289371/Cie13/Q1/NW/2024
PX124492/aMPV-B/chicken/USA/GA/11293896/Cie12/I/NE/2024
aMPV-B/turkey/USA/NC/ADRDL-26/2024
aMPV-B/chicken/USA/NC/ADRDL-46/2026
PX124516/aMPV-B/chicken/USA/GA/11283957/Cie9/K/NE/2024
PX124511/aMPV-B/chicken/USA/GA/11283261/Cie3/X/NE/2024
PP273461/aMPV-B/chicken/NC/USA/ADRDL-6/2024
aMPV-B/turkey/USA/NC/ADRDL-43/2024
84
aMPV-B/turkey/USA/NC/ADRDL-17/2024
aMPV-B/turkey/USA/NC/ADRDL-19/2024
aMPV-B/turkey/USA/NC/ADRDL-41/2024
aMPV-B/turkey/USA/PA/ADRDL-38/2024
aMPV-B/chicken/USA/NC/ADRDL-12/2025
aMPV-B/turkey/USA/NC/ADRDL-40/2024
aMPV-B/chicken/USA/NC/ADRDL-11/2024
aMPV-B/turkey/USA/VA/ADRDL-37/2024
aMPV-B/turkey/USA/NC/ADRDL-22/2024
aMPV-B/turkey/USA/NC/ADRDL-23/2024
aMPV-B/turkey/USA/NC/ADRDL-34/2024
aMPV-B/turkey/USA/NC/ADRDL-24/2024
aMPV-B/turkey/USA/ND/ADRDL-10/2024
aMPV-B/chicken/USA/SC/ADRDL-8/2024
aMPV-B/turkey/USA/NC/ADRDL-18/2024
PX124508/aMPV-B/chicken/USA/GA/11284269.1/Cie4/M/S/2024
aMPV-B/turkey/USA/PA/ADRDL-7/2024
PX124512/aMPV-B/chicken/USA/GA/11282879/Cie8/I1/NE/2024
aMPV-B/turkey/USA/MO/ADRDL-53/2026
PX124480/aMPV-B/chicken/USA/GA/11283954.3/Cie9/K/NE/2024
aMPV-B/turkey/USA/NC/ADRDL-21/2024
aMPV-B/turkey/USA/NC/ADRDL-33/2024
PP273460/aMPV-B/turkey/VA/USA/ADRDL-5/2024
PV067047/aMPV-B/chicken/USA/NC23734-GA/PrimaryCells/P1/2024
aMPV-B/turkey/USA/NC/ADRDL-32/2024
aMPV-B/turkey/USA/NC/ADRDL-20/2024
PP273459/aMPV-B/turkey/VA/USA/ADRDL-4/2024
aMPV-B/turkey/USA/NC/ADRDL-36/2024
aMPV-B/turkey/USA/NC/ADRDL-15/2024
PP273456/aMPV-B/turkey/NC/USA/ADRDL-1/2024
PV067043/aMPV-B/turkey/USA/NC20487-GA/PrimaryCells/P3/2024
aMPV-B/chicken/USA/SC/ADRDL-13/2024
PX124502/aMPV-B/chicken/USA/GA/11284246/Cie12/L/NE/2024
PX124500/aMPV-B/Chicken/USA/11284397/Cie4/M/S/2024
PX124509/aMPV-B/chicken/USA/GA/11284396/Cie4/M/S/2024
PX124507/aMPV-B/chicken/USA/GA/11281545.2/Cie6/C1/NE/2024
aMPV-B/turkey/USA/VA/ADRDL-9/2024
aMPV-B/turkey/USA/NC/ADRDL-27/2024
aMPV-B/turkey/USA/VA/ADRDL-31/2024
PX124513/aMPV-B/chicken/USA/GA/11282511/Cie9/H/NE/2024
PX124503/aMPV-B/chicken/USA/GA/11284245d/Cie12/U/NE/2024
aMPV-B/turkey/USA/SD/ADRDL-39/2024
PX124515/aMPV-B/chicken/USA/GA/11283951/Cie3/X/NE/2024
aMPV-B/turkey/USA/NC/ADRDL-16/2024
PQ382890/aMPV-B/turkey/IA/SEP-RS1/2024
aMPV-B/turkey/USA/NC/ADRDL-42/2024
PX124514/aMPV-B/chicken/USA/GA/11281543/Cie6/E1/NE/2024
PV872165/aMPV-B/chicken/USA/SD-24/P6-2024
aMPV-B/turkey/USA/NC/ADRDL-30/2024
aMPV-B/turkey/USA/NC/ADRDL-28/2024
aMPV-B/turkey/USA/NC/ADRDL-14/2024
aMPV-B/chicken/USA/NC/ADRDL-35/2024
PX124506/aMPV-B/chicken/USA/GA/11281620/Cie3/D1/NE/2024
aMPV-B/turkey/USA/NC/ADRDL-29/2024
PP273457/aMPV-B/turkey/NC/USA/ADRDL-2/2024
PX124504/aMPV-B/chicken/USA/GA/11283954.1/Cie9/K/NE/2024
PP273458/aMPV-B/turkey/VA/USA/ADRDL-3/2024
aMPV-B/chicken/USA/NC/ADRDL-44/2025
aMPV-B/turkey/USA/NC/ADRDL-25/2024
aMPV-B/turkey/USA/MN/ADRDL-45/2025
0.003
0.00
0.02
0.04
0.06
GenBank references
Study samples
Vaccine references
Vaccine-derived
Chicken
Turkey

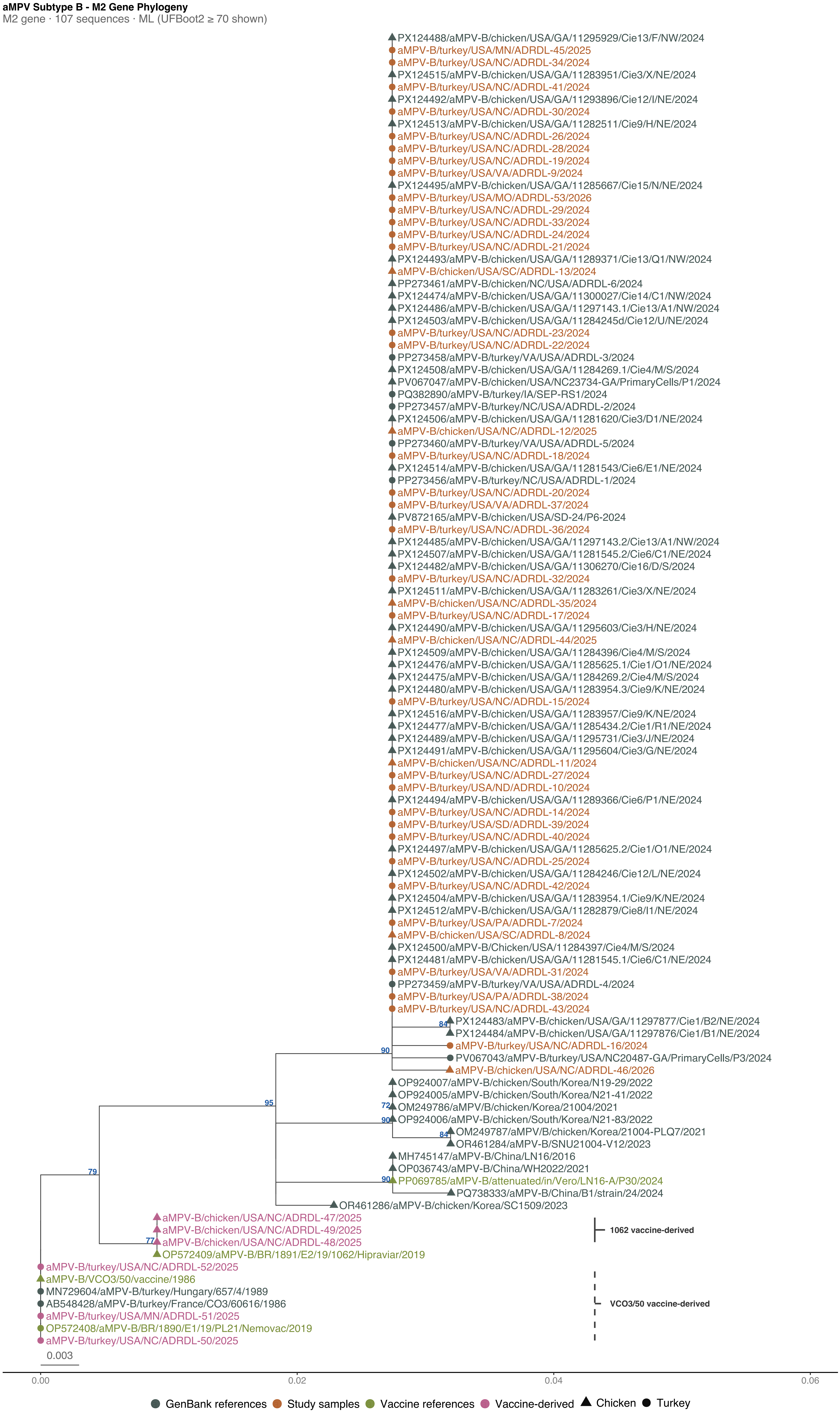

aMPV Subtype B - M2 Gene Phylogeny
M2 gene · 107 sequences · ML (UFBoot2 ≥ 70 shown)
PX124488/aMPV-B/chicken/USA/GA/11295929/Cie13/F/NW/2024
aMPV-B/turkey/USA/MN/ADRDL-45/2025
aMPV-B/turkey/USA/NC/ADRDL-34/2024
PX124515/aMPV-B/chicken/USA/GA/11283951/Cie3/X/NE/2024
aMPV-B/turkey/USA/NC/ADRDL-41/2024
PX124492/aMPV-B/chicken/USA/GA/11293896/Cie12/I/NE/2024
aMPV-B/turkey/USA/NC/ADRDL-30/2024
PX124513/aMPV-B/chicken/USA/GA/11282511/Cie9/H/NE/2024
aMPV-B/turkey/USA/NC/ADRDL-26/2024
aMPV-B/turkey/USA/NC/ADRDL-28/2024
aMPV-B/turkey/USA/NC/ADRDL-19/2024
aMPV-B/turkey/USA/VA/ADRDL-9/2024
PX124495/aMPV-B/chicken/USA/GA/11285667/Cie15/N/NE/2024
aMPV-B/turkey/USA/MO/ADRDL-53/2026
aMPV-B/turkey/USA/NC/ADRDL-29/2024
aMPV-B/turkey/USA/NC/ADRDL-33/2024
aMPV-B/turkey/USA/NC/ADRDL-24/2024
aMPV-B/turkey/USA/NC/ADRDL-21/2024
PX124493/aMPV-B/chicken/USA/GA/11289371/Cie13/Q1/NW/2024
aMPV-B/chicken/USA/SC/ADRDL-13/2024
PP273461/aMPV-B/chicken/NC/USA/ADRDL-6/2024
PX124474/aMPV-B/chicken/USA/GA/11300027/Cie14/C1/NW/2024
PX124486/aMPV-B/chicken/USA/GA/11297143.1/Cie13/A1/NW/2024
PX124503/aMPV-B/chicken/USA/GA/11284245d/Cie12/U/NE/2024
aMPV-B/turkey/USA/NC/ADRDL-23/2024
aMPV-B/turkey/USA/NC/ADRDL-22/2024
PP273458/aMPV-B/turkey/VA/USA/ADRDL-3/2024
PX124508/aMPV-B/chicken/USA/GA/11284269.1/Cie4/M/S/2024
PV067047/aMPV-B/chicken/USA/NC23734-GA/PrimaryCells/P1/2024
PQ382890/aMPV-B/turkey/IA/SEP-RS1/2024
PP273457/aMPV-B/turkey/NC/USA/ADRDL-2/2024
PX124506/aMPV-B/chicken/USA/GA/11281620/Cie3/D1/NE/2024
aMPV-B/chicken/USA/NC/ADRDL-12/2025
PP273460/aMPV-B/turkey/VA/USA/ADRDL-5/2024
aMPV-B/turkey/USA/NC/ADRDL-18/2024
PX124514/aMPV-B/chicken/USA/GA/11281543/Cie6/E1/NE/2024
PP273456/aMPV-B/turkey/NC/USA/ADRDL-1/2024
aMPV-B/turkey/USA/NC/ADRDL-20/2024
aMPV-B/turkey/USA/VA/ADRDL-37/2024
PV872165/aMPV-B/chicken/USA/SD-24/P6-2024
aMPV-B/turkey/USA/NC/ADRDL-36/2024
PX124485/aMPV-B/chicken/USA/GA/11297143.2/Cie13/A1/NW/2024
PX124507/aMPV-B/chicken/USA/GA/11281545.2/Cie6/C1/NE/2024
PX124482/aMPV-B/chicken/USA/GA/11306270/Cie16/D/S/2024
aMPV-B/turkey/USA/NC/ADRDL-32/2024
PX124511/aMPV-B/chicken/USA/GA/11283261/Cie3/X/NE/2024
aMPV-B/chicken/USA/NC/ADRDL-35/2024
aMPV-B/turkey/USA/NC/ADRDL-17/2024
PX124490/aMPV-B/chicken/USA/GA/11295603/Cie3/H/NE/2024
aMPV-B/chicken/USA/NC/ADRDL-44/2025
PX124509/aMPV-B/chicken/USA/GA/11284396/Cie4/M/S/2024
PX124476/aMPV-B/chicken/USA/GA/11285625.1/Cie1/O1/NE/2024
PX124475/aMPV-B/chicken/USA/GA/11284269.2/Cie4/M/S/2024
PX124480/aMPV-B/chicken/USA/GA/11283954.3/Cie9/K/NE/2024
aMPV-B/turkey/USA/NC/ADRDL-15/2024
PX124516/aMPV-B/chicken/USA/GA/11283957/Cie9/K/NE/2024
PX124477/aMPV-B/chicken/USA/GA/11285434.2/Cie1/R1/NE/2024
PX124489/aMPV-B/chicken/USA/GA/11295731/Cie3/J/NE/2024
PX124491/aMPV-B/chicken/USA/GA/11295604/Cie3/G/NE/2024
aMPV-B/chicken/USA/NC/ADRDL-11/2024
aMPV-B/turkey/USA/NC/ADRDL-27/2024
aMPV-B/turkey/USA/ND/ADRDL-10/2024
PX124494/aMPV-B/chicken/USA/GA/11289366/Cie6/P1/NE/2024
aMPV-B/turkey/USA/NC/ADRDL-14/2024
aMPV-B/turkey/USA/SD/ADRDL-39/2024
aMPV-B/turkey/USA/NC/ADRDL-40/2024
PX124497/aMPV-B/chicken/USA/GA/11285625.2/Cie1/O1/NE/2024
aMPV-B/turkey/USA/NC/ADRDL-25/2024
PX124502/aMPV-B/chicken/USA/GA/11284246/Cie12/L/NE/2024
aMPV-B/turkey/USA/NC/ADRDL-42/2024
PX124504/aMPV-B/chicken/USA/GA/11283954.1/Cie9/K/NE/2024
PX124512/aMPV-B/chicken/USA/GA/11282879/Cie8/I1/NE/2024
aMPV-B/turkey/USA/PA/ADRDL-7/2024
aMPV-B/chicken/USA/SC/ADRDL-8/2024
PX124500/aMPV-B/Chicken/USA/11284397/Cie4/M/S/2024
PX124481/aMPV-B/chicken/USA/GA/11281545.1/Cie6/C1/NE/2024
aMPV-B/turkey/USA/VA/ADRDL-31/2024
PP273459/aMPV-B/turkey/VA/USA/ADRDL-4/2024
aMPV-B/turkey/USA/PA/ADRDL-38/2024
aMPV-B/turkey/USA/NC/ADRDL-43/2024
84
PX124483/aMPV-B/chicken/USA/GA/11297877/Cie1/B2/NE/2024
PX124484/aMPV-B/chicken/USA/GA/11297876/Cie1/B1/NE/2024
90
aMPV-B/turkey/USA/NC/ADRDL-16/2024
PV067043/aMPV-B/turkey/USA/NC20487-GA/PrimaryCells/P3/2024
aMPV-B/chicken/USA/NC/ADRDL-46/2026
95
OP924007/aMPV-B/chicken/South/Korea/N19-29/2022
OP924005/aMPV-B/chicken/South/Korea/N21-41/2022
72
OM249786/aMPV/B/chicken/Korea/21004/2021
90
OP924006/aMPV-B/chicken/South/Korea/N21-83/2022
84
OM249787/aMPV/B/chicken/Korea/21004-PLQ7/2021
OR461284/aMPV-B/SNU21004-V12/2023
79
MH745147/aMPV-B/China/LN16/2016
OP036743/aMPV-B/China/WH2022/2021
90
PP069785/aMPV-B/attenuated/in/Vero/LN16-A/P30/2024
PQ738333/aMPV-B/China/B1/strain/24/2024
OR461286/aMPV-B/chicken/Korea/SC1509/2023
aMPV-B/chicken/USA/NC/ADRDL-47/2025
aMPV-B/chicken/USA/NC/ADRDL-49/2025
77
aMPV-B/chicken/USA/NC/ADRDL-48/2025
OP572409/aMPV-B/BR/1891/E2/19/1062/Hipraviar/2019
1062 vaccine-derived
aMPV-B/turkey/USA/NC/ADRDL-52/2025
aMPV-B/VCO3/50/vaccine/1986
MN729604/aMPV-B/turkey/Hungary/657/4/1989
AB548428/aMPV-B/turkey/France/CO3/60616/1986
aMPV-B/turkey/USA/MN/ADRDL-51/2025
OP572408/aMPV-B/BR/1890/E1/19/PL21/Nemovac/2019
aMPV-B/turkey/USA/NC/ADRDL-50/2025
VCO3/50 vaccine-derived
0.003
0.00
0.02
0.04
0.06
GenBank references
Study samples
Vaccine references
Vaccine-derived
Chicken
Turkey

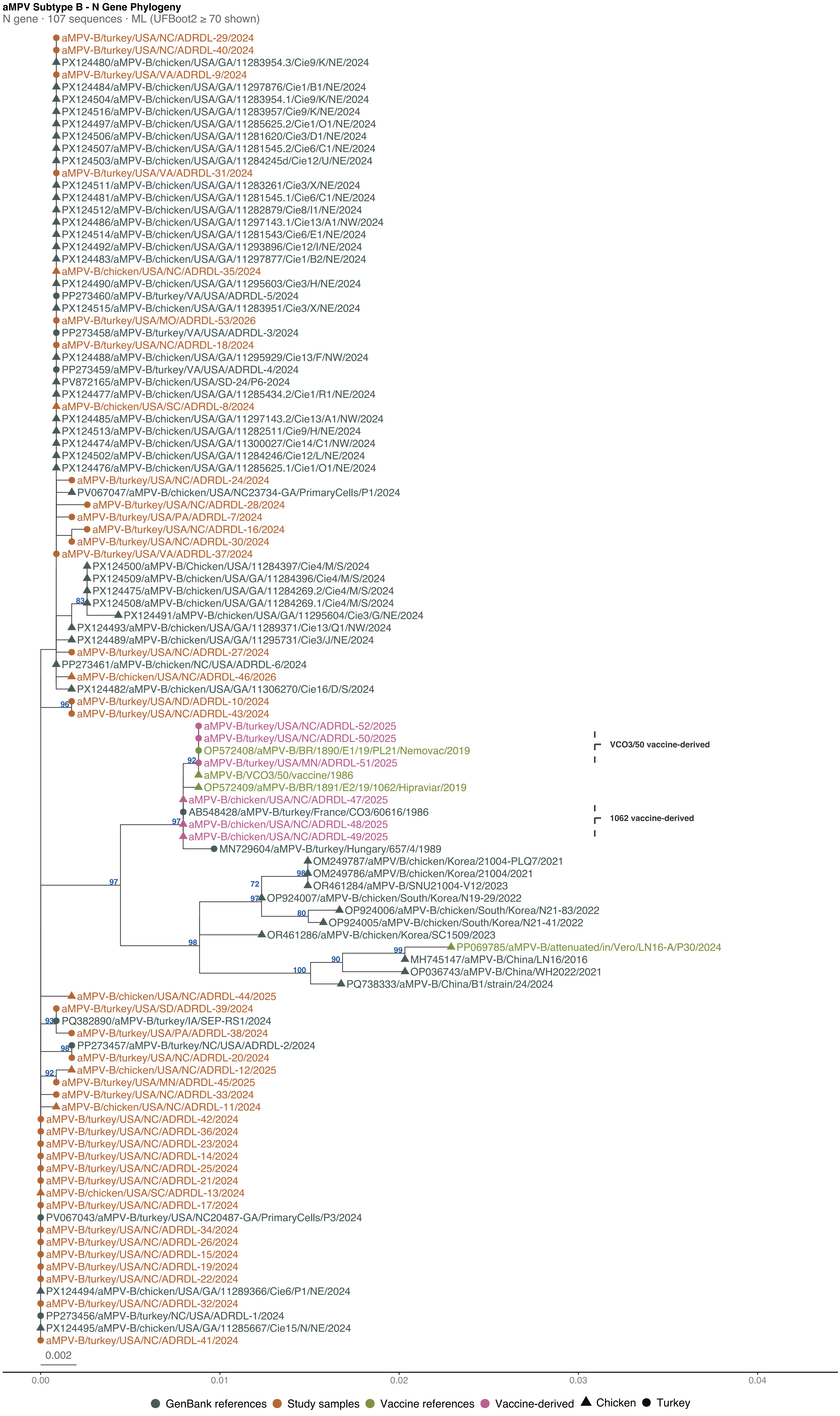
aMPV Subtype B - N Gene Phylogeny
N gene · 107 sequences · ML (UFBoot2 ≥ 70 shown)
aMPV-B/turkey/USA/NC/ADRDL-29/2024
aMPV-B/turkey/USA/NC/ADRDL-40/2024
PX124480/aMPV-B/chicken/USA/GA/11283954.3/Cie9/K/NE/2024
aMPV-B/turkey/USA/VA/ADRDL-9/2024
PX124484/aMPV-B/chicken/USA/GA/11297876/Cie1/B1/NE/2024
PX124504/aMPV-B/chicken/USA/GA/11283954.1/Cie9/K/NE/2024
PX124516/aMPV-B/chicken/USA/GA/11283957/Cie9/K/NE/2024
PX124497/aMPV-B/chicken/USA/GA/11285625.2/Cie1/O1/NE/2024
PX124506/aMPV-B/chicken/USA/GA/11281620/Cie3/D1/NE/2024
PX124507/aMPV-B/chicken/USA/GA/11281545.2/Cie6/C1/NE/2024
PX124503/aMPV-B/chicken/USA/GA/11284245d/Cie12/U/NE/2024
aMPV-B/turkey/USA/VA/ADRDL-31/2024
PX124511/aMPV-B/chicken/USA/GA/11283261/Cie3/X/NE/2024
PX124481/aMPV-B/chicken/USA/GA/11281545.1/Cie6/C1/NE/2024
PX124512/aMPV-B/chicken/USA/GA/11282879/Cie8/I1/NE/2024
PX124486/aMPV-B/chicken/USA/GA/11297143.1/Cie13/A1/NW/2024
PX124514/aMPV-B/chicken/USA/GA/11281543/Cie6/E1/NE/2024
PX124492/aMPV-B/chicken/USA/GA/11293896/Cie12/I/NE/2024
PX124483/aMPV-B/chicken/USA/GA/11297877/Cie1/B2/NE/2024
aMPV-B/chicken/USA/NC/ADRDL-35/2024
PX124490/aMPV-B/chicken/USA/GA/11295603/Cie3/H/NE/2024
PP273460/aMPV-B/turkey/VA/USA/ADRDL-5/2024
PX124515/aMPV-B/chicken/USA/GA/11283951/Cie3/X/NE/2024
aMPV-B/turkey/USA/MO/ADRDL-53/2026
PP273458/aMPV-B/turkey/VA/USA/ADRDL-3/2024
aMPV-B/turkey/USA/NC/ADRDL-18/2024
PX124488/aMPV-B/chicken/USA/GA/11295929/Cie13/F/NW/2024
PP273459/aMPV-B/turkey/VA/USA/ADRDL-4/2024
PV872165/aMPV-B/chicken/USA/SD-24/P6-2024
PX124477/aMPV-B/chicken/USA/GA/11285434.2/Cie1/R1/NE/2024
aMPV-B/chicken/USA/SC/ADRDL-8/2024
PX124485/aMPV-B/chicken/USA/GA/11297143.2/Cie13/A1/NW/2024
PX124513/aMPV-B/chicken/USA/GA/11282511/Cie9/H/NE/2024
PX124474/aMPV-B/chicken/USA/GA/11300027/Cie14/C1/NW/2024
PX124502/aMPV-B/chicken/USA/GA/11284246/Cie12/L/NE/2024
PX124476/aMPV-B/chicken/USA/GA/11285625.1/Cie1/O1/NE/2024
aMPV-B/turkey/USA/NC/ADRDL-24/2024
PV067047/aMPV-B/chicken/USA/NC23734-GA/PrimaryCells/P1/2024
aMPV-B/turkey/USA/NC/ADRDL-28/2024
aMPV-B/turkey/USA/PA/ADRDL-7/2024
aMPV-B/turkey/USA/NC/ADRDL-16/2024
aMPV-B/turkey/USA/NC/ADRDL-30/2024
aMPV-B/turkey/USA/VA/ADRDL-37/2024
PX124500/aMPV-B/Chicken/USA/11284397/Cie4/M/S/2024
PX124509/aMPV-B/chicken/USA/GA/11284396/Cie4/M/S/2024
PX124475/aMPV-B/chicken/USA/GA/11284269.2/Cie4/M/S/2024
83
PX124508/aMPV-B/chicken/USA/GA/11284269.1/Cie4/M/S/2024
PX124491/aMPV-B/chicken/USA/GA/11295604/Cie3/G/NE/2024
PX124493/aMPV-B/chicken/USA/GA/11289371/Cie13/Q1/NW/2024
PX124489/aMPV-B/chicken/USA/GA/11295731/Cie3/J/NE/2024
aMPV-B/turkey/USA/NC/ADRDL-27/2024
PP273461/aMPV-B/chicken/NC/USA/ADRDL-6/2024
aMPV-B/chicken/USA/NC/ADRDL-46/2026
PX124482/aMPV-B/chicken/USA/GA/11306270/Cie16/D/S/2024
96
aMPV-B/turkey/USA/ND/ADRDL-10/2024
aMPV-B/turkey/USA/NC/ADRDL-43/2024
aMPV-B/turkey/USA/NC/ADRDL-52/2025
aMPV-B/turkey/USA/NC/ADRDL-50/2025
OP572408/aMPV-B/BR/1890/E1/19/PL21/Nemovac/2019
VCO3/50 vaccine-derived
92
aMPV-B/turkey/USA/MN/ADRDL-51/2025
aMPV-B/VCO3/50/vaccine/1986
OP572409/aMPV-B/BR/1891/E2/19/1062/Hipraviar/2019
aMPV-B/chicken/USA/NC/ADRDL-47/2025
AB548428/aMPV-B/turkey/France/CO3/60616/1986
97
aMPV-B/chicken/USA/NC/ADRDL-48/2025
1062 vaccine-derived
aMPV-B/chicken/USA/NC/ADRDL-49/2025
MN729604/aMPV-B/turkey/Hungary/657/4/1989
97
OM249787/aMPV/B/chicken/Korea/21004-PLQ7/2021
98
OM249786/aMPV/B/chicken/Korea/21004/2021
72
OR461284/aMPV-B/SNU21004-V12/2023
97
OP924007/aMPV-B/chicken/South/Korea/N19-29/2022
80
OP924006/aMPV-B/chicken/South/Korea/N21-83/2022
OP924005/aMPV-B/chicken/South/Korea/N21-41/2022
98
OR461286/aMPV-B/chicken/Korea/SC1509/2023
99
PP069785/aMPV-B/attenuated/in/Vero/LN16-A/P30/2024
90
MH745147/aMPV-B/China/LN16/2016
100
OP036743/aMPV-B/China/WH2022/2021
PQ738333/aMPV-B/China/B1/strain/24/2024
aMPV-B/chicken/USA/NC/ADRDL-44/2025
aMPV-B/turkey/USA/SD/ADRDL-39/2024
93
PQ382890/aMPV-B/turkey/IA/SEP-RS1/2024
aMPV-B/turkey/USA/PA/ADRDL-38/2024
98
PP273457/aMPV-B/turkey/NC/USA/ADRDL-2/2024
aMPV-B/turkey/USA/NC/ADRDL-20/2024
aMPV-B/chicken/USA/NC/ADRDL-12/2025
92
aMPV-B/turkey/USA/MN/ADRDL-45/2025
aMPV-B/turkey/USA/NC/ADRDL-33/2024
aMPV-B/chicken/USA/NC/ADRDL-11/2024
aMPV-B/turkey/USA/NC/ADRDL-42/2024
aMPV-B/turkey/USA/NC/ADRDL-36/2024
aMPV-B/turkey/USA/NC/ADRDL-23/2024
aMPV-B/turkey/USA/NC/ADRDL-14/2024
aMPV-B/turkey/USA/NC/ADRDL-25/2024
aMPV-B/turkey/USA/NC/ADRDL-21/2024
aMPV-B/chicken/USA/SC/ADRDL-13/2024
aMPV-B/turkey/USA/NC/ADRDL-17/2024
PV067043/aMPV-B/turkey/USA/NC20487-GA/PrimaryCells/P3/2024
aMPV-B/turkey/USA/NC/ADRDL-34/2024
aMPV-B/turkey/USA/NC/ADRDL-26/2024
aMPV-B/turkey/USA/NC/ADRDL-15/2024
aMPV-B/turkey/USA/NC/ADRDL-19/2024
aMPV-B/turkey/USA/NC/ADRDL-22/2024
PX124494/aMPV-B/chicken/USA/GA/11289366/Cie6/P1/NE/2024
aMPV-B/turkey/USA/NC/ADRDL-32/2024
PP273456/aMPV-B/turkey/NC/USA/ADRDL-1/2024
PX124495/aMPV-B/chicken/USA/GA/11285667/Cie15/N/NE/2024
aMPV-B/turkey/USA/NC/ADRDL-41/2024
0.002
0.00
0.01
0.02
0.03
0.04
GenBank references
Study samples
Vaccine references
Vaccine-derived
Chicken
Turkey

aMPV Subtype B - P Gene Phylogeny

P gene · 107 sequences · ML (UFBoot2 ≥ 70 shown)

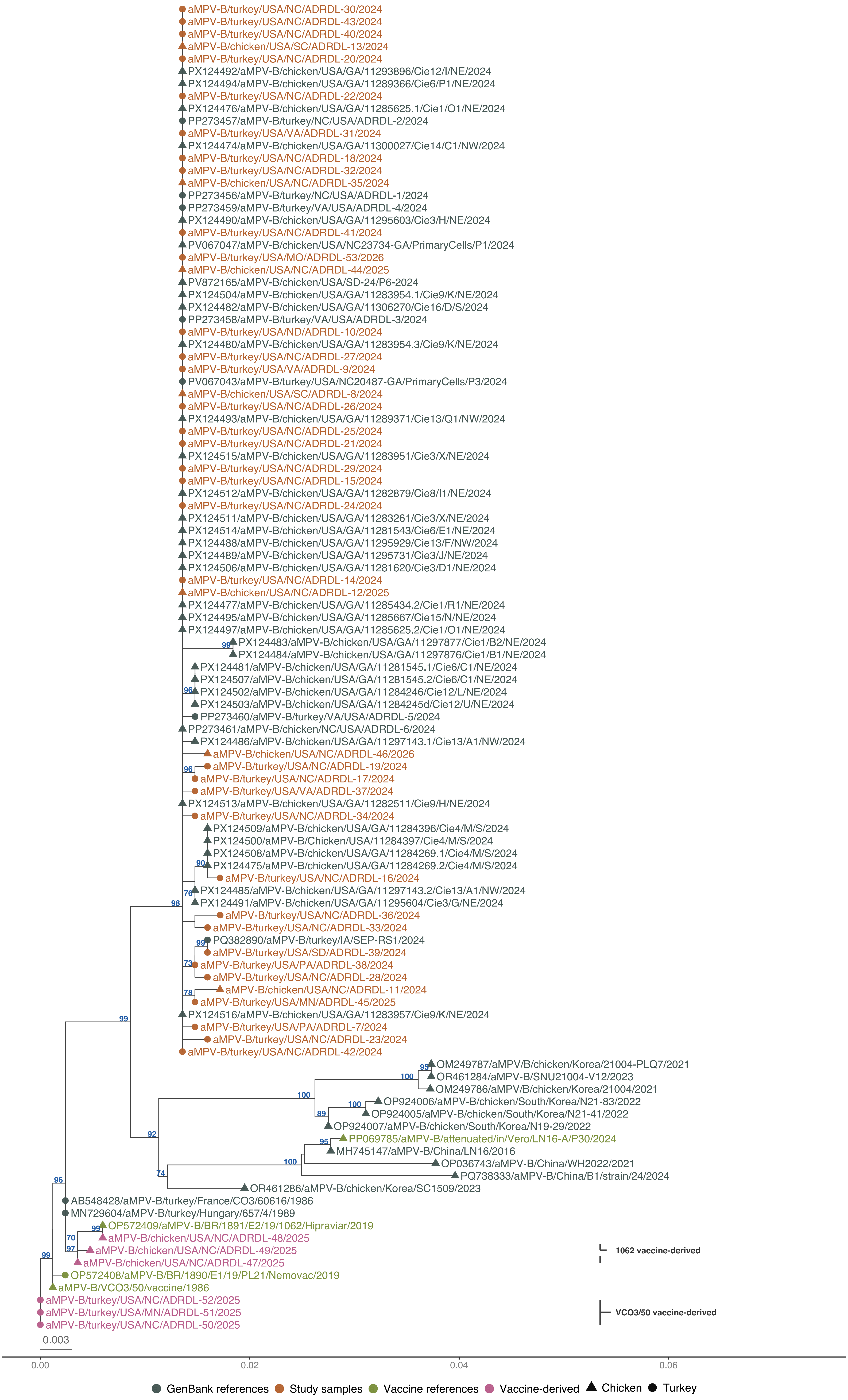

aMPV Subtype B - SH Gene Phylogeny

SH gene · 107 sequences · ML (UFBoot2 ≥ 70 shown)

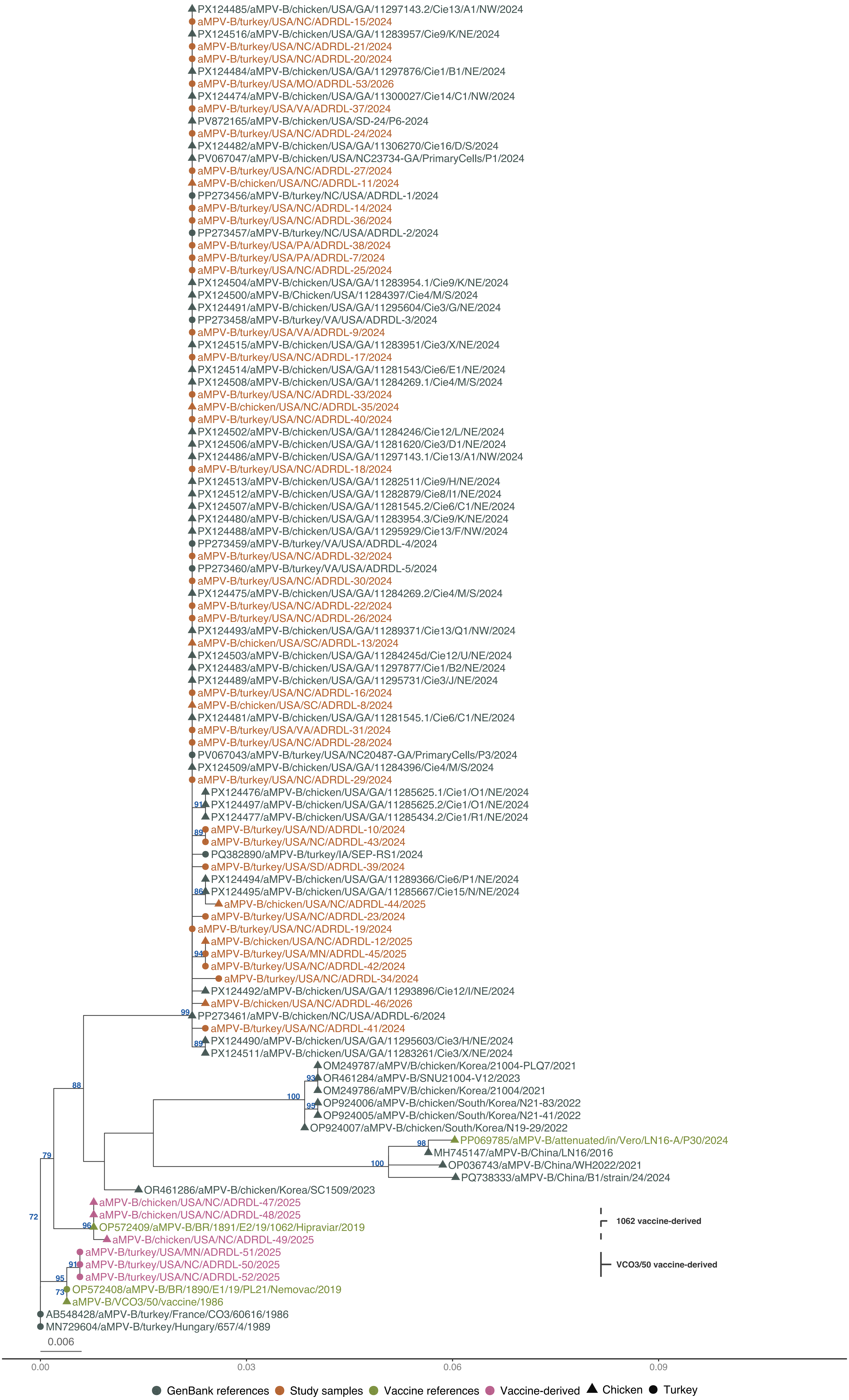

Supplement: Supplementary file 6 [file DataSheet5.pdf]
