# Supplementary material for: Genomic characterization of avian metapneumovirus subtypes A and B in United States poultry by targeted amplicon sequencing
Source: Front Cell Infect Microbiol. 2026 Aug 21;16:1882507. doi: 10.3389/fcimb.2026.1882507 (PMC13541459; doi:10.3389/fcimb.2026.1882507)
Supplement: Supplementary file 7 [file DataSheet6.pdf]

Subtype A Vaccine-Lineage Marker States

A Subtype A vaccine-lineage marker positions on the aMPV-A genome

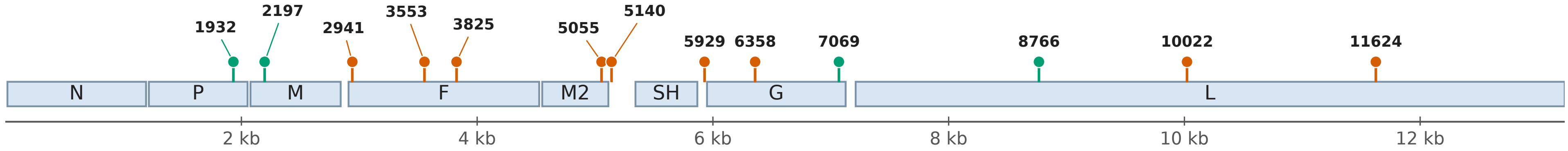

B Subtype A vaccine-lineage nucleotide states

	1932 P	2197 M	2941 M-F int.	3553 F	3825 F	5055 M2	5140 M2	5929 SH-G int.	6358 G	7069 G	8766 L	10022 L	11624 L
Vaccine 1	**C**	**C**	**A**	**C**	**A**	**G**	**C**	**G**	**C**	**T**	**C**	**G**	**C**
Italy vaccine-derived (JF424833)	**A**	**A**	**A**	**T**	**A**	**G**	**C**	**G**	**C**	**C**	**T**	**G**	**C**
Vaccine progenitor (DQ666911)	**C**	**C**	**G**	**T**	**G**	**A**	**T**	**A**	**T**	**T**	**C**	**T**	**T**
ADRDL-43	C	A	A	T	A	G	T	G	C	T	C	G	C
ADRDL-44	C	A	A	T	A	G	C	G	C	T	C	G	C
ADRDL-45	C	A	A	T	A	G	C	G	C	T	C	G	C
ADRDL-46	C	A	A	T	A	G	C	G	C	T	C	G	C
ADRDL-47	C	A	A	T	A	G	C	G	C	T	C	G	C
ADRDL-48	C	A	A	T	A	G	C	G	C	T	C	G	C

Matches Vaccine 1
Matches UK#8544 progenitor
Matches IT/259 lineage state
N/ambiguous
Vaccine-defining marker
IT/259-specific marker
