# Supplementary material for: Genomic characterization of avian metapneumovirus subtypes A and B in United States poultry by targeted amplicon sequencing
Source: Front Cell Infect Microbiol. 2026 Aug 21;16:1882507. doi: 10.3389/fcimb.2026.1882507 (PMC13541459; doi:10.3389/fcimb.2026.1882507)
Supplement: Supplementary file 8 [file DataSheet7.pdf]

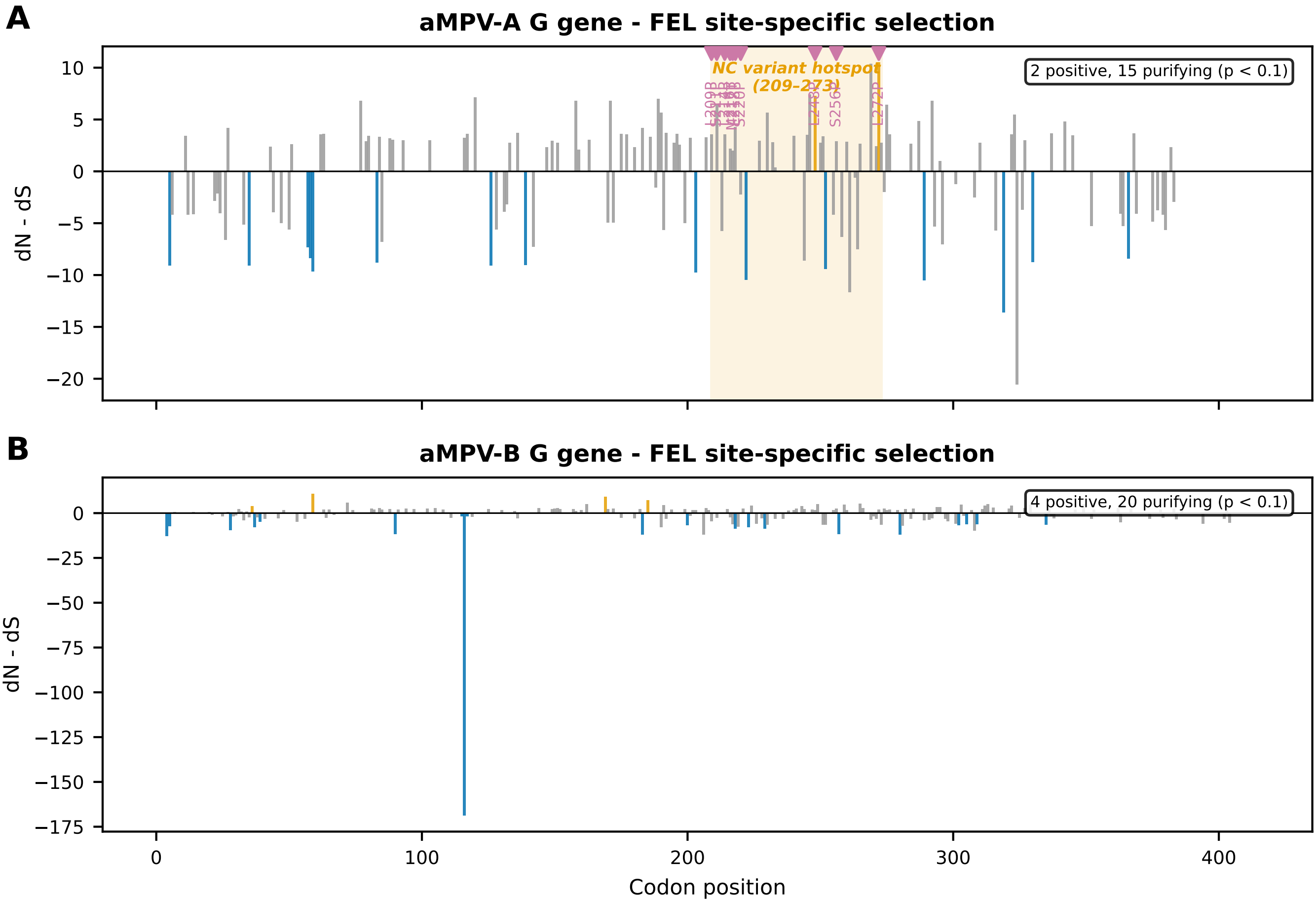
A
aMPV-A G gene - FEL site-specific selection
NC variant hotspot (209–273)
2 positive, 15 purifying (p < 0.1)
L248P
S256P
L272P
dN - dS
10
5
0
−5
−10
−15
−20
B
aMPV-B G gene - FEL site-specific selection
4 positive, 20 purifying (p < 0.1)
dN - dS
0
−25
−50
−75
−100
−125
−150
−175
0
100
200
300
400
Codon position
