# Supplementary material for: Genomic characterization of avian metapneumovirus subtypes A and B in United States poultry by targeted amplicon sequencing
Source: Front Cell Infect Microbiol. 2026 Aug 21;16:1882507. doi: 10.3389/fcimb.2026.1882507 (PMC13541459; doi:10.3389/fcimb.2026.1882507)
Supplement: Supplementary file 9 [file DataSheet8.pdf]

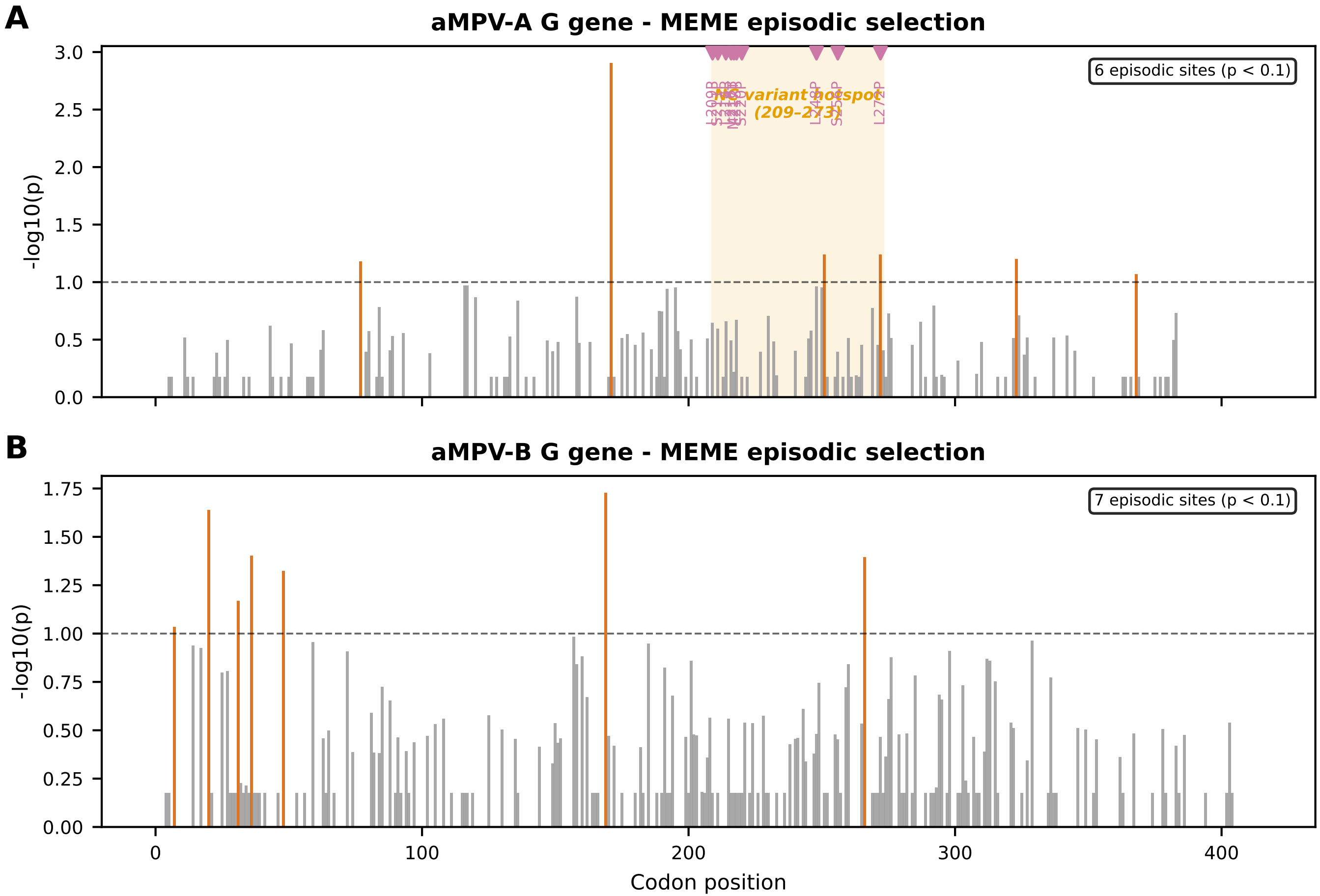
A
aMPV-A G gene - MEME episodic selection
6 episodic sites (p < 0.1)
variant hotspot
(209–273)
L209P
L248P
S256P
L272P
-log10(p)
B
aMPV-B G gene - MEME episodic selection
7 episodic sites (p < 0.1)
-log10(p)
Codon position
