# Supplementary material for: Genomic characterization of avian metapneumovirus subtypes A and B in United States poultry by targeted amplicon sequencing
Source: Front Cell Infect Microbiol. 2026 Aug 21;16:1882507. doi: 10.3389/fcimb.2026.1882507 (PMC13541459; doi:10.3389/fcimb.2026.1882507)
Supplement: Supplementary file 10 [file DataSheet9.pdf]

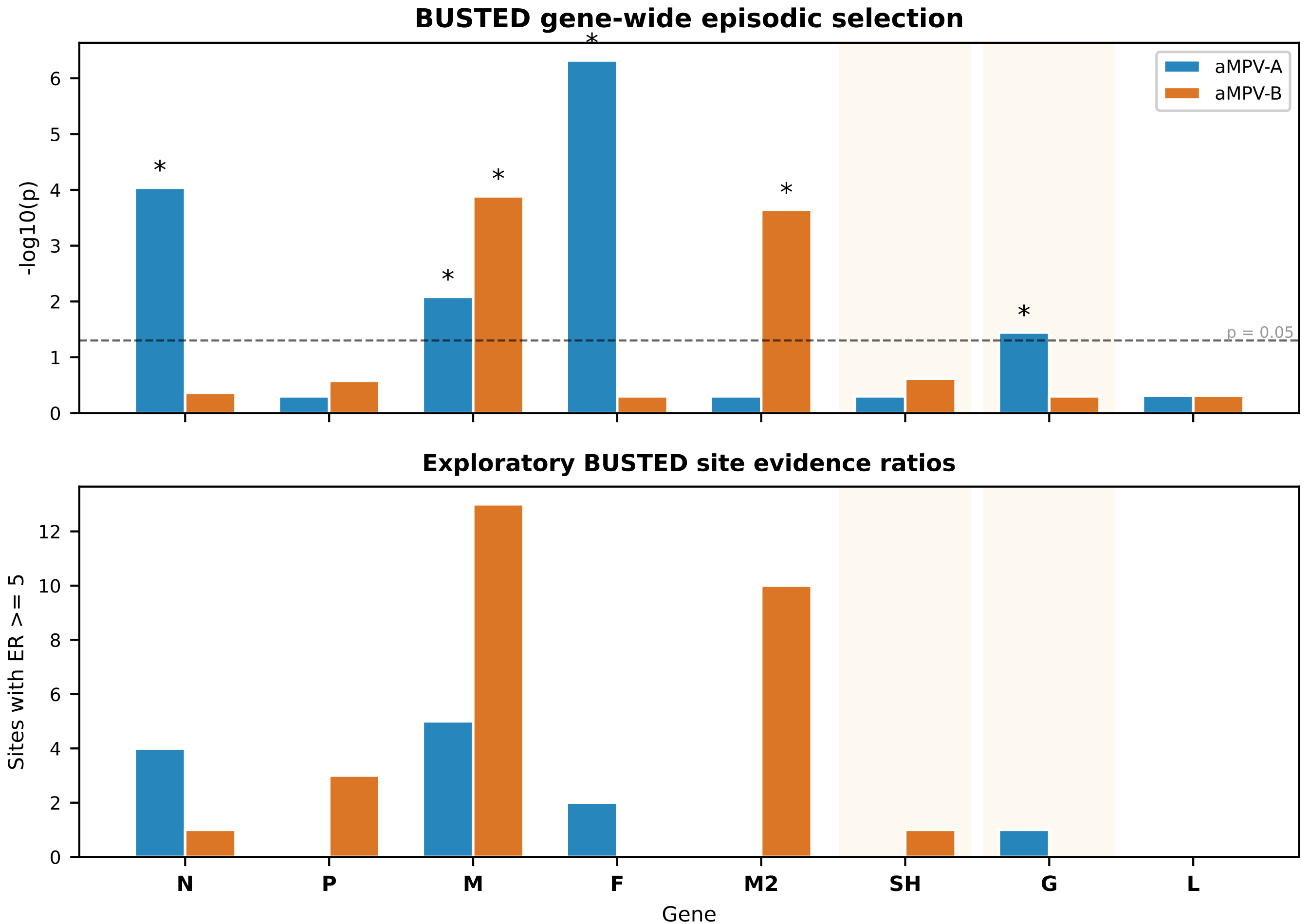
BUSTED gene-wide episodic selection
aMPV-A
aMPV-B
-log10(p)
p = 0.05
Exploratory BUSTED site evidence ratios
Sites with ER >= 5
N
P
M
F
M2
SH
G
L
Gene
